# Mitochondrial DNA alterations may influence the cisplatin responsiveness of oral squamous cell carcinoma

**DOI:** 10.1038/s41598-020-64664-3

**Published:** 2020-05-12

**Authors:** Amnani Aminuddin, Pei Yuen Ng, Chee-Onn Leong, Eng Wee Chua

**Affiliations:** 1 Drug and Herbal Research Centre, Faculty of Pharmacy, Universiti Kebangsaan Malaysia, Jalan Raja Muda Abdul Aziz, 50300 Kuala Lumpur Malaysia; 2 School of Pharmacy, International Medical University, Bukit Jalil, 57000 Kuala Lumpur Malaysia; 3 Centre for Cancer and Stem Cell Research, Institute for Research, Development and Innovation, International Medical University, Bukit Jalil, 57000 Kuala Lumpur Malaysia

**Keywords:** Predictive markers, Cancer genetics

## Abstract

Cisplatin is the first-line chemotherapeutic agent for the treatment of oral squamous cell carcinoma (OSCC). However, the intrinsic or acquired resistance against cisplatin remains a major obstacle to treatment efficacy in OSCC. Recently, mitochondrial DNA (mtDNA) alterations have been reported in a variety of cancers. However, the role of mtDNA alterations in OSCC has not been comprehensively studied. In this study, we evaluated the correlation between mtDNA alterations (mtDNA content, point mutations, large-scale deletions, and methylation status) and cisplatin sensitivity using two OSCC cell lines, namely SAS and H103, and stem cell-like tumour spheres derived from SAS. By microarray analysis, we found that the tumour spheres profited from aberrant lipid and glucose metabolism and became resistant to cisplatin. By qPCR analysis, we found that the cells with less mtDNA were less responsive to cisplatin (H103 and the tumour spheres). Based on the findings, we theorised that the metabolic changes in the tumour spheres probably resulted in mtDNA depletion, as the cells suppressed mitochondrial respiration and switched to an alternative mode of energy production, *i.e*. glycolysis. Then, to ascertain the origin of the variation in mtDNA content, we used MinION, a nanopore sequencer, to sequence the mitochondrial genomes of H103, SAS, and the tumour spheres. We found that the lower cisplatin sensitivity of H103 could have been caused by a constellation of genetic and epigenetic changes in its mitochondrial genome. Future work may look into how changes in mtDNA translate into an impact on cell function and therefore cisplatin response.

## Introduction

*Cis*-diamminedichloroplatinum (II), or cisplatin, is one of the most commonly used chemotherapy agents in the treatment of various solid tumours such as ovarian, colorectal, prostate, lung, and head and neck tumours^1–5^. To date, the intrinsic or acquired resistance of cancer cells to cisplatin remains a challenge in the chemotherapy of several cancers including oral squamous cell carcinoma (OSCC)^3,6^. OSCC, which affects the epithelial layer of the oral cavity, is a common malignant tumour of the head and neck with low survival rates and high risks of recurrence^7^.

The well-characterized mode of action of cisplatin is via causing the formation of DNA adducts upon its binding to the nucleophilic N7 sites of purines, which further leads to DNA damage responses and apoptosis^2,6,8^. Cisplatin resistance in general involves reduced DNA damage due to an increase in DNA adduct repair, reduced drug uptake, or increased drug inactivation^1,3,4,6^. Activation of these mechanisms depends on multiple factors including genetic changes, epigenetic alterations at both molecular and cellular levels, and heterogeneity among cancer cells^4,9,10^.

The recently proposed cancer stem cells (CSCs) model highlighted tumour heterogeneity as an important basis of treatment resistance and relapse in cancer. According to the model, CSCs comprise a tumourigenic subpopulation where they exhibit stem cell-like features including the abilities to self-renew and differentiate into different cell lineages, thus giving rise to tumour heterogeneity. Conventional chemotherapy effectively removes rapidly proliferating cancer cells in a bulk tumour but fails to eliminate CSCs, which are protected by mechanisms of therapeutic resistance. Subsequently, the surviving CSCs initiate new populations of cancer cells, which are more drug-resistant and aggressive^11–13^.

Furthermore, cisplatin primarily targets mitochondrial DNA (mtDNA) to induce apoptosis; its binding to nuclear DNA is limited^14,15^. Interestingly, mtDNA alterations have been implicated in the development of cancer and chemoresistance^14,16,17^. However, the potential effect of the modifications in the mitochondrial genome on OSCC treatment has not been comprehensively studied. High-throughput sequencing has enabled comprehensive surveys of cancer genomes and helped us to elucidate cisplatin resistance^1,18^. Currently available commercial second-generation sequencing platforms, such as MiSeq and Ion Torrent, can produce a large volume of sequencing data at a low cost. Nevertheless, the short read length and the use of PCR amplification in preparing sequencing libraries are the major limitations of these sequencers. This has prompted the invention of third-generation sequencing platforms^19^. The latest nanopore sequencer (MinION) devised by Oxford Nanopore Technologies circumvents some of the limitations of the older technologies. The device houses a dense array of buffer-submerged, nanosized pores through which single DNA strands are allowed to pass. The passage of DNA strands produces distinctive ionic signals that are converted into DNA sequences. Because nanopore sequencing needs only minimal pre-sequencing preparation, it can produce very long reads (sometimes > 50 kb), minimise potential nucleotide errors introduced by PCR amplification, and preserve epigenetic modifications such as DNA methylation^20^.

In this work, we derived tumour spheres from two OSCC cell lines with differing cisplatin sensitivity. We first characterized the stem cell-like features of the tumour spheres using flow cytometry, Western blotting, and microarray analysis. Then, we used MinION to determine the influence of a variety of mtDNA alterations on cisplatin responsiveness in OSCC. We also measured the levels of intracellular reactive oxygen species (ROS) to gauge the effect of cisplatin exposure on mitochondrial function. Finally, we pondered the relation between mitochondria and cisplatin response. Understanding the molecular markers of cisplatin responsiveness in OSCC may help us to counter cisplatin resistance in the clinical setting.

## Results and discussion

### Enhanced tumour sphere-forming capacity of OSCC SAS cells

**Figure 1:**
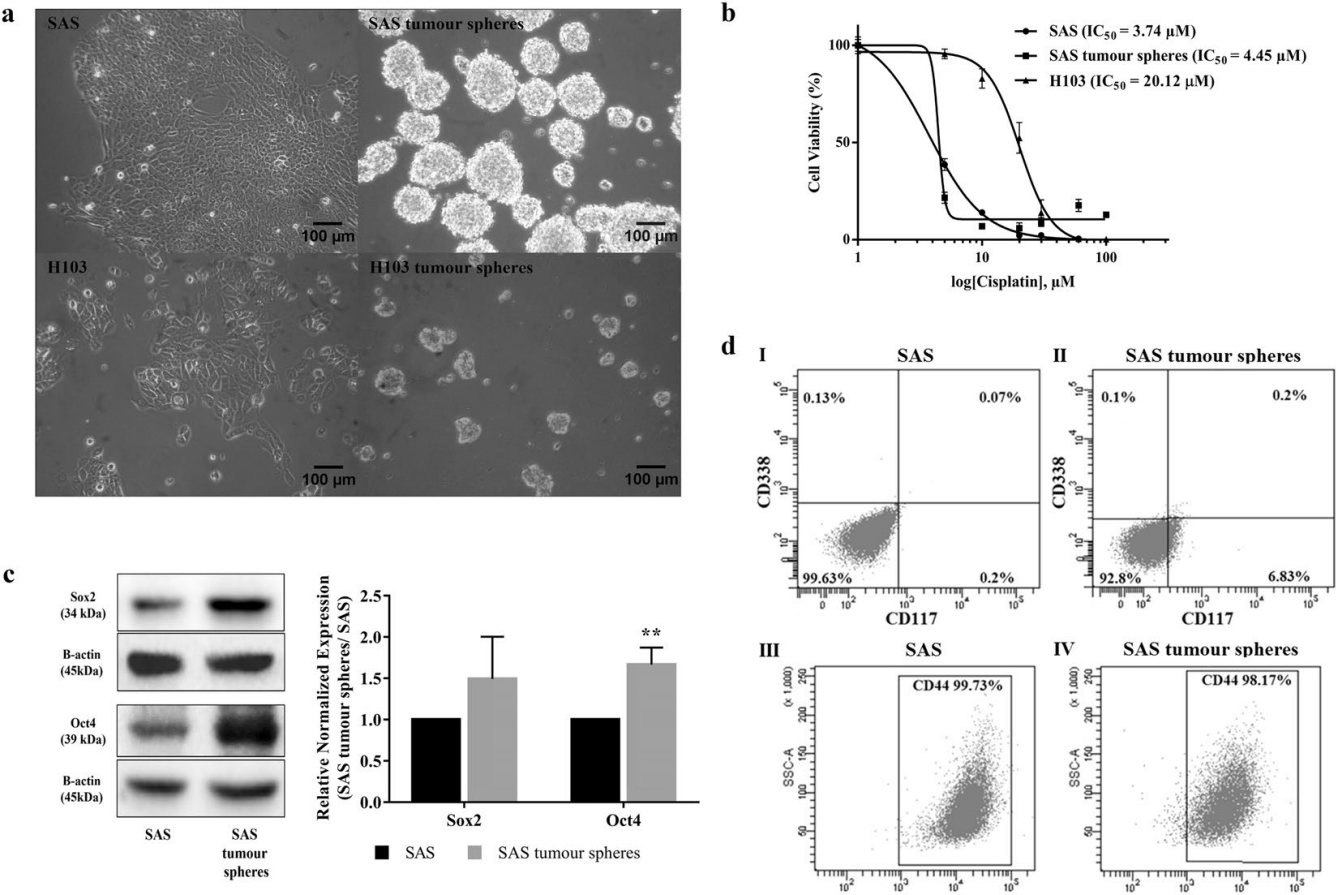
Derivation of cancer stem cells (CSCs) from OSCC cell lines via a sphere-forming assay and the characterization of their stem cell-like features. (**a**) The morphology of the parental SAS and H103 and their derived tumour spheres. SAS and H103 in normal culture media were observed as polygonal squamous epithelial cells with the adherent growth pattern. Within 7 d, tumour spheres, comprised of aggregated and suspended cells derived from SAS and H103, were formed in the specialized serum-free medium containing serum substitute, heparin, and growth factors and in a low attachment plate (100× magnification). The average diameters of the SAS and H103 tumour spheres were 133.4 ± 34.36 µm and 68.1 ± 13.37 µm, respectively. (**b**) Assessment of cell viability of SAS, SAS tumour spheres, and H103 after 72 h exposure to cisplatin. IC_50_ was defined as the concentration of cisplatin required to reduce cell viability by half. Higher IC_50_ values indicated lower sensitivity of the cells towards cisplatin and possibly cisplatin resistance. (**c**) Western blots of Sox2, Oct4 and β-actin and the relative expression levels of the Sox2 and Oct4 transcription factors normalized to the β-actin protein in SAS and SAS tumour spheres. The full-length blots are presented in Supplementary Figure S2. (**d**) Expression of CD338, CD117 and CD44 surface markers in both SAS and SAS tumour spheres, as analyzed by flow cytometry. Multi-staining flow cytometry was used to analyse the surface expression of CD338 and CD117 for (I) SAS and (II) SAS tumour spheres. Single-staining flow cytometry was used to analyse the surface expression of CD44 for (III) SAS and (IV) SAS tumour spheres. All the data are presented as mean ± SD. **P < 0.01, *n* = 3.

The tumour spheres formation assay has been reported to successfully enrich CSCs derived from cell lines of several solid tumours, namely breast cancer, lung cancer, ovarian cancer, hepatocellular carcinoma, osteosarcoma, fibrosarcoma, and OSCC^21–27^. The assay allows the enrichment of cells with stem cell traits without prior knowledge of their surface markers, in contrast to the side population method, which sorts and isolates CSCs based on specific and predefined surface markers^28^. This could be an advantage since the biomarker profiles of the CSCs of many cancers are still lacking. Furthermore, spherical models provide a three-dimensional microenvironment for the cells to grow, allowing them to mimic the *in vivo* behaviour of cancer cells more closely than when they are cultured in monolayers (the conventional two-dimensional model)^29^. We found that SAS formed tumour spheres more efficiently than H103 (Fig. 1a). H103 formed fewer and smaller spheres, possibly because they were less responsive to growth factors, their parental cells were innately less active, or they had decreased self-renewal capacity^30^. We could not obtain sufficient H103 tumour spheres for downstream analyses; therefore, they were excluded from this study.

### SAS tumour spheres exhibited OSCC stemness protein surface marker CD117

By flow cytometry, we investigated the surface expression of several stemness-related markers that are known to be present on CSCs derived from OSCC, namely CD117, CD338, and CD44. CD117 or c-Kit, a receptor tyrosine kinase protein, is a marker for hematopoietic stem and progenitor cells, ovarian cancer-initiating cells isolated from primary human tumours, cardiac CD117+ stem cells, and CSCs derived from OSCC^31^. CD338, also known as ABCG2, is a member of a family of ATP-binding cassette drug transporter proteins that expel drugs from cells. Overexpression of CD338 has been linked to chemoresistance of CSCs in OSCC^21,32,33^. In cancers, CD44 acts as a cell surface adhesion receptor and promotes the proliferation, survival, and metastasis of tumour cells^28,34–37^. We found that the expression of CD117 in SAS tumour spheres was significantly higher than that in SAS (P = 0.008; Fig. 1d); but, CD338 was only weakly expressed on the surfaces of both SAS and SAS tumour spheres (0.13% and 0.10% respectively), and the surface expression of CD44 did not differ significantly (P = 0.065) between them (Fig. 1d). We suggest that CD338 may not be a definitive marker for CSCs derived from OSCC. In breast and prostate cancers, both CD338-positive and -negative cells isolated by the side population technique were similarly tumourigenic, and the CD338-negative population also contained primitive stem-like cancer cells^38^. The link between CD44 and OSCC stemness is also unclear because CD44 exists as several alternatively spliced isoforms with varied relevance to cancer growth. It has been reported that a transcript isoform of CD44, CD44v3, is a more specific CSC surface marker for head and neck cancers, as the isoform is expressed preferentially on cancer cells for tumourigenesis^39,40^. The importance of CD44 to CSCs may be masked when the different isoforms are analysed indiscriminately. For instance, both CD44-positive and -negative cell populations in head and neck squamous carcinoma were reported in a study to possess CSC traits^41^.

### SAS tumour spheres demonstrated enhanced cisplatin resistance

We assessed cisplatin sensitivity based on the number of cells that survived after 72 h exposure to varied doses of cisplatin. From the cell viability evaluation, we found SAS tumour spheres (IC_50_ of 4.45 µM; P = 0.013) and H103 (IC_50_ of 20.12 µM; P = 0.0001) to be less sensitive towards cisplatin than SAS (IC_50_ of 3.74 µM; Fig. 1b). Accumulating evidence has shown that CSCs become resistant to DNA damage-induced cell death by altering their metabolism, heightening ROS-scavenging activity, and activating DNA repair and anti-apoptotic pathways that include Wnt, Notch, and PI3K signalling^42–46^.

### SAS tumour spheres with stem cell-like features showed increased expressions of metabolism-associated and pluripotency genes

**Figure 2:**
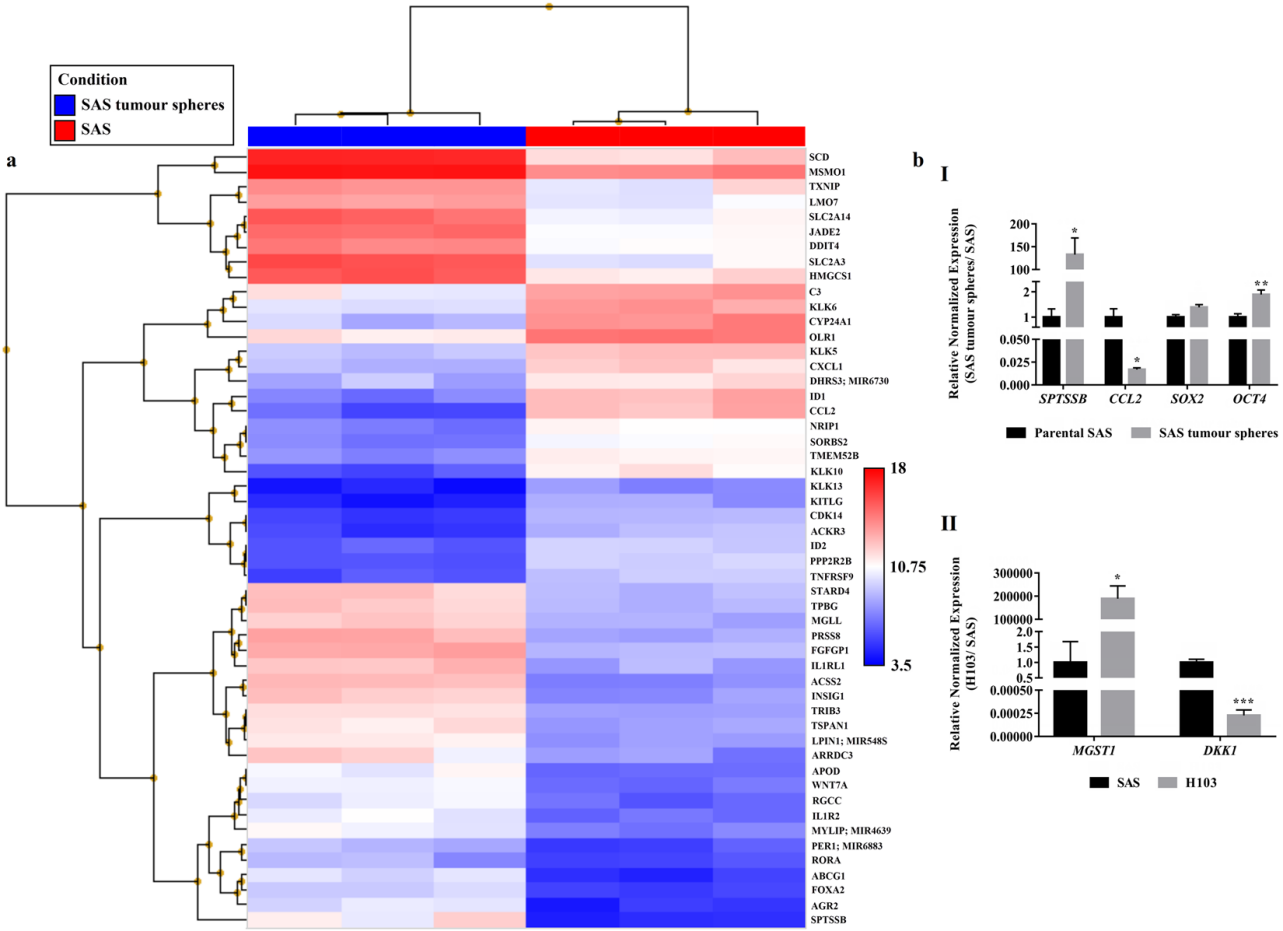
The transcriptomic profiles of SAS cells and their derived tumour spheres as analysed using the Affymetrix Clariom S arrays. (**a**) Heat map generated from the microarray data reflecting log2 normalised gene expression values using the Robust Multi-array Average method, where the p-value adjusted for the false discovery rate was less than 0.05 and the positive or negative fold change exceeded 10. Blue represents lower gene expression and red represents higher gene expression. *n* = 3. (**b**) Microarray validation through qPCR for the top up- or down-regulated genes in (I) SAS tumour spheres and (II) H103 relative to SAS. Expression of stemness-associated genes, *OCT4* and *SOX2*, were also measured by qPCR in SAS and SAS tumour spheres. The amplification levels of the genes were normalised against two reference genes, *ACTB* and *GAPDH*. Data are presented as mean ± SD. *P < 0.05, **P < 0.01, ***P < 0.001, *n* = 3.

Through microarray analysis, we found that the SAS tumour spheres expressed some genes differently than their parental cells. Of the 21488 genes interrogated, 32 were substantially up-regulated (fold change > 10) and 20 were down-regulated (fold change < −10) in the SAS tumour spheres (Fig. 2a). The list of differentially expressed genes (DEGs) is provided in Supplementary Table S1. Further pathway enrichment analysis of the DEGs revealed that the metabolic phenotype of the SAS tumour spheres was significantly altered (Table 1). We speculated that the metabolic changes were attendant upon the formation of the tumour spheres, which were rendered metabolically similar to CSCs. We observed significant increases in the expression of *SLC2A3* (P = 0.0012) and *SLC2A14* (P = 0.0021), both of which encode glucose transporters, in SAS tumour spheres compared to SAS. Overexpression of the genes suggests an increase in the glucose uptake activity in the tumour spheres and a shift to the glycolytic metabolism for energy production^47,48^. Moreover, the expression levels of some lipid metabolism-related genes, namely *SPTSSB*, *SCD*, *ABCG1, INSIG1, HMGCS1, STARD4, TRIB3, LPIN1, MGLL, RORA*, and *MSMO1*, were also significantly higher in the SAS tumour spheres than the parental SAS cells (Table 1 and Supplementary Table S1; P < 0.02). CSCs depend on aberrations in glucose and lipid metabolism for sustenance. They switch to glycolysis to evade damage that could result from the high levels of ROS inevitably produced during oxidative phosphorylation (OXPHOS). This enables CSCs to self-renew infinitely. Furthermore, many studies have shown that increased lipid synthesis helps to maintain CSCs. It is an important source of metabolic intermediates and energy needed for cell growth and stemness-related pathways^47,49–53^.

**Table 1:** Lists of the top five up- or down-regulated genes in SAS tumour spheres compared to SAS, and their associated functional pathways catalogued from the Reactome database. Genes associated with the regulation of the pluripotency of stem cells and whose expression was upregulated in SAS tumour spheres are also included in the table.

Gene	Encoded protein	Fold change	Associated functional pathways
**Up-regulated gene**			
*SPTSSB*	Serine palmitoyltransferase, small subunit B	83.61	Sphingolipids* de novo* biosynthesis (metabolism of lipids; metabolism)
*SLC2A3* (also known as *GLUT3*)	Solute carrier family 2 (facilitated glucose transporter), member 3	59.16	Cellular hexose transport (SLC-mediated transmembrane transport; transport of small molecules); Vitamin C (ascorbate) metabolism (metabolism of vitamins and cofactor; metabolism); Neutrophil degranulation (innate immune system; immune system); Transcriptional regulation by MECP2 (RNA polymerase II transcription; gene expression (transcription))
*ACSS2*	Acyl-CoA synthetase short-chain family member 2	50.18	Transcriptional activation of mitochondrial biogenesis (mitochondrial biogenesis; organelle biogenesis and maintenance); Ethanol oxidation (biological oxidations; metabolism)
*SCD*	Stearoyl-CoA desaturase (delta-9-desaturase)	38.94	Fatty acyl-CoA biosynthesis (metabolism of lipids; metabolism); Activation of gene expression by SREBF (SREBP) (metabolism of lipids; metabolism)
*PRSS8*	Protease, serine, 8	37.3	Formation of the cornified envelope (keratinization; developmental biology)
*KLF4*	Kruppel-like factor 4	2.4	Transcriptional regulation of pluripotent stem cells (developmental biology)
*POU5F1 (OCT4)*	POU class 5 homeobox 1	1.45	
*SALL4*	Spalt-like transcription factor 4	1.16	
*SOX2*	SRY box 2	1.03	
*LIN28A*	Lin-28 homolog A	1.01	
*ZSCAN10*	Zinc finger and SCAN domain containing 10	1.09	
**Down-regulated genes**			
*CCL2*	Chemokine (C-C motif) ligand 2	−151.6	Interleukin- 4, 10 and 13 signalling (cytokine signalling; immune system); ATF4 activates genes in response to endoplasmic reticulum stress (unfolded protein response; metabolism of proteins)
*KLK10*	Kallikrein related peptidase 10	−38.53	Collagen chain trimerization (collagen formation; extracellular matrix organization); Macrophage-stimulating protein-Recepteur d’origine nantais (MSP-RON) kinase signaling (signalling by MST1; receptor tyrosine kinases signalling; signal transduction)
*ID1*	Inhibitor of DNA binding 1, dominant negative helix-loop-helix protein	−36.73	Oncogene induced senescence (cellular responses to external stimuli)
*CYP24A1*	Cytochrome P450, family 24, subfamily A, polypeptide 1	−31.78	Vitamin D (calciferol) metabolism (metabolism of lipids, metabolism); Cytochrome P450 - arranged by substrate type (biological oxidations; metabolism); Defective CYP24A1 causes hypercalcemia, infantile (HCAI) (disease of metabolism; disease)
*KITLG*	KIT ligand	−17.11	Regulation of KIT signalling (SCF-KIT signalling; receptor tyrosine kinases signalling; signal transduction); RAF/MAP kinase cascade (FLT3 signalling; cytokine signalling; immune system); Other interleukin signalling (cytokine signalling; immune system); Constitutive signalling by aberrant PI3K in cancer (PI3K/AKT signalling in cancer; diseases of signal transduction); RAF/MAP kinase cascade (MAPK1/MAPK3 signalling; MAPK family signalling cascades, signal transduction); PI5P, PP2A and IER3 regulate PI3K/AKT signalling (negative regulation of the PI3K/AKT network; intracellular signalling by second messengers; signal transduction)

From the microarray data, we evaluated the expression levels of the genes that regulate the pluripotency or stemness of cancer cells. We found that the SAS tumour spheres expressed *KLF4*, *OCT4, SALL4, SOX2, LIN28A*, and *ZSCAN10* more strongly than SAS (Table 1). The increased expression levels of two of the genes, namely *OCT4* (P = 0.016) and *SOX2* (P = 0.052), and the proteins they encode (Oct4, P = 0.004; Sox2, P = 0.17) were confirmed by qPCR (Fig. 2b) and Western blotting (Fig. 1c). Klf4, Oct4, Sox2, Sall4, Lin28A, and Zscan10 are transcription factors that maintain the pluripotency and the self-renewal capacity of embryonic stem cells^54–58^ and CSCs of breast, laryngeal, gastrointestinal, brain, liver, and oral cancers^21,31,42,59–64^.

### MtDNA content was correlated with cisplatin resistance in OSCC

MtDNA content is tightly regulated by the energy requirement of a cell or tissue, which varies between cell and tissue types or developmental stages. For instance, low mtDNA content is often observed in cancer cells and pluripotent cells as they rely on glycolysis instead of OXPHOS for energy production^65^. In addition, some cancer cells transition into a pseudo-differentiated state that renders them unable to replicate their mtDNA and establish mtDNA set points^66^. Indeed, prior studies have shown that oxidative stress and several pathological conditions, including cancer, alter mtDNA content^67,68^. However, the reported relation between mtDNA content and cancer has been inconsistent. Increased mtDNA content has been linked to advanced stages of oesophageal squamous cell carcinoma and head and neck squamous cell carcinoma^69,70^. Reduced mtDNA content has been associated with invasive forms of lung cancer, ovarian carcinoma, and breast cancer^71–73^.

**Figure 3:**
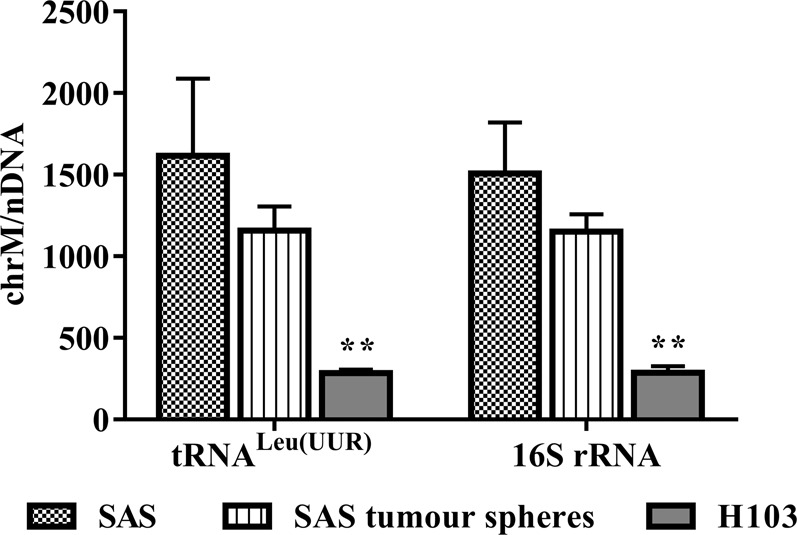
qPCR estimation of mtDNA content. The amplification levels of two mitochondrial genes, tRNA^Leu(UUR)^ and 16S rRNA, were normalised against that of a nuclear gene, β2-microglobulin. Data are presented as mean ± SD. **P < 0.01, *n* = 3.

In this study, we estimated mtDNA content by qPCR. Overall, we found that the cells with lower mtDNA content were less responsive to cisplatin. As shown in Fig. 3, H103, which was more cisplatin-resistant, had significantly lower mtDNA content than SAS (P < 0.01). Our observation coincides with that of another study, which showed that mtDNA content increased in the transition of head and neck squamous cell carcinoma from low to high histopathological grades^69^. Similarly, SAS tumour spheres had less mtDNA than SAS and were more cisplatin-resistant, though the difference was not significant (P > 0.10). Depleted mtDNA content was previously demonstrated in CSCs and treatment-resistant cancer cells^74–76^.

### MtDNA content was correlated with the extent of ROS production induced by cisplatin

**Figure 4:**
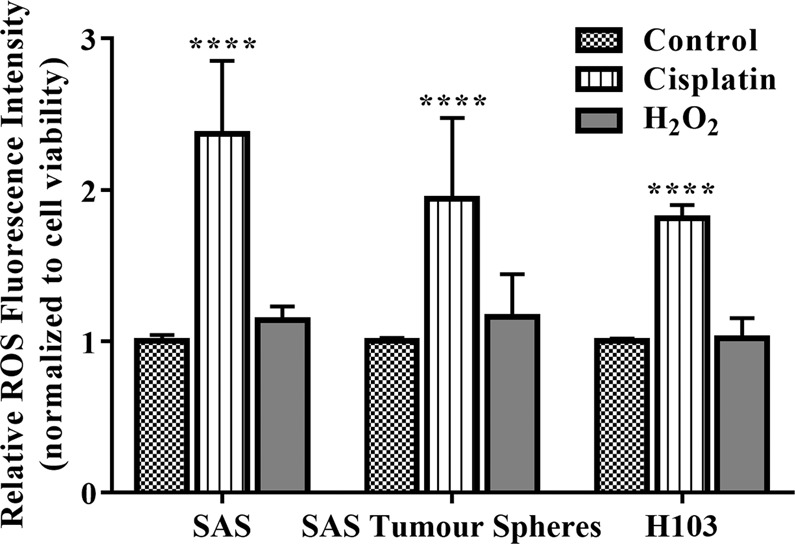
Measurement of the changes in intracellular ROS levels after treatment with cisplatin for 72 h. The data are presented in means ± SD of ROS levels relative to an untreated control group and normalised against the percentage of viable cells. ****P < 0.0001, against an untreated control group, *n* = 3.

Other works on hepatoma and small cell lung cancer reported that low mtDNA content reduced the sensitivity of cancer cells to ROS-induced cytotoxicity by: 1) causing a compensatory increase in the expression of antioxidant enzymes; 2) impairing mitochondrial respiration; and 3) increasing mitochondrial membrane potential (mitochondrial outer membrane permeability was decreased as a result)^77,78^. In general, apart from directly disrupting the structure of mtDNA, cisplatin also induces the formation of ROS inside cells, causing oxidative stress and further DNA damage. We found that the cells with lower mtDNA content were less sensitive to ROS-induced cytotoxicity, confirming prior findings that variation in mtDNA content marks the progress of malignant cells in their transformation into death-resistant tumours. Both the SAS tumour spheres and H103 had lower mtDNA content than SAS and produced correspondingly less intracellular ROS after cisplatin treatment (Fig. 4; P < 0.0001). Here, we suggest that the relation between mtDNA content and the extent of ROS production induced by cisplatin may indicate the ability of cancer cells to profit from mitochondrial dysfunction and evade death. Further investigations should look into how variation in mtDNA content translates into an impact on cell function and cisplatin sensitivity. Besides, the influence of mtDNA content variation on cisplatin sensitivity may also depend on tissue and/or tumour types. A study of laryngeal, nasopharyngeal, and lung cancers reported a contrasting observation where increased mtDNA content is a self-protective tactic used by tumour cells to evade apoptosis. A reduction in mtDNA content was found to inhibit antioxidant gene expression, increase intracellular ROS levels, and sensitise tumour cells to chemotherapy^79^.

### Overview of the MinION sequencing data

Six MinION sequencing runs were performed using two MinION SpotOn Flow Cells as described in Table 2. Each MinION sequencing run generated single-directional (1D) sequencing reads in which a single DNA strand was ‘read’ only once. The overall read and mapping statistics for each run are provided in Table 3. We observed large variation in the total sequencing output using the MinION flow cells, which may be attributed to the quality and performance of the flow cells. We also observed that the number of active pores declined substantially after use, decreasing the sequencing output. Nevertheless, we found that the quality of the raw data produced from a new or used flow cell was equally satisfactory, as more than 50% of the base-called reads had a minimum quality score of 5 (Data shown in Supplementary Table S2).

**Table 2:** Six sequencing runs performed successively using two SpotOn Flow Cells.

SpotOn Flow Cell	Sequencing Number	Sample	Sample processing
1	1	SAS	Long PCR-amplification and purification
	2	SAS	Linearisation and purification
	3	H103	Long PCR-amplification, purification, and limited barcoding PCR
2	4	SAS tumour spheres	Long PCR-amplification, purification, and limited barcoding PCR
	5	SAS tumour spheres	Linearisation and purification
	6	H103	Linearisation and purification

**Table 3:** An overview of the MinION sequencing data.

	SAS (PCR amplicon)	SAS tumour spheres (PCR amplicon)	H103 (PCR amplicon)	SAS (Native)	SAS tumour spheres (Native)	H103 (Native)
**Read Statistics**						
Total reads	25561	110442	3564	7361	16560	5110
Proportion of passed reads (%)	99.7 (25476/ 25561)	99.8 (110229/ 110442)	99.0 (3530/ 3564)	99.1 (7297/ 7361)	98.4 (16303/ 16560)	97.3 (4970/ 5110)
Total length (base)	79485220	237092104	11851813	28484148	58638164	17924412
Maximum length (base)	151048	180437	94896	122623	1414982	128173
Median read length	2043	1517	2214.5	2631	2146	2259
Mean read length	3120	2150.9	3357.45	3903.54	3596.77	3606.52
**Mapping Statistics**						
Proportion of reads aligned to ChrM (%)	49.2 (12546/ 25476)	22.9 (25282/ 110229)	10.3 (365/ 3530)	4.3 (314/ 7297)	2.4 (395/ 16303)	1.4 (68/4970)
Total alignment length (base)	234267	114847	27277	50959	57073	34527
Pairwise identity (%)	68.1	72.1	72.4	61.7	55.6	58.7
GC content (%)	45.4	44.5	44.3	46.4	45.6	46.4
Mean read length	2955.2	1192	1815.9	4763.6	3592.4	3994.9

The read statistics from the Albacore base-called reads were generated using NanoStat. The mapping statistics were based on the MinION reads aligned to the human mitochondrial genome (GRCh38) with a mapping quality score of at least 20.

When comparing the protocols for library preparation, we noted that native DNA libraries produced lower proportions of on-target reads than PCR amplicons (SAS: 4.3% vs. 49.2%; SAS tumour spheres: 2.4% vs. 22.9%; H103: 1.4% vs. 10.3%; Table 3). Nevertheless, using only the sequence reads from the native DNA libraries, we could still assemble a complete profile of the mitochondrial genome with adequate coverage (only for SAS and SAS tumour spheres with average depths of coverage of 60.4 and 55.3, respectively). This suggests that the method we used for mtDNA extraction^80^ was effective at enriching the mtDNA fraction. Being able to directly sequence mtDNA is important, as it preserves the methylation status of the samples.

### No difference in the mitochondrial genomes of SAS tumour spheres and their parental cells

We used MinION to sequence the mitochondrial genomes of our samples (SAS, SAS tumour spheres, and H103) with different sensitivity towards cisplatin. By cross-checking the variants identified by MinION with Sanger sequencing, we found that 95.7% of the variants were correctly called. The accuracy of the variant calling was similar to what was reported in other studies (Data shown in Supplementary Table S3)^81–83^.

We detected 50 mtDNA variants in total for SAS and SAS tumour spheres, and 24% of the variants were observed in the displacement loop of the mitochondrial genome (D-loop). The other variants were found across the coding regions of the mitochondrial genome. Overall, we found that the variants observed in SAS were also present in SAS tumour spheres, though several variants significantly differed in their allele fractions between the two samples (Table 4). However, we could not determine the functional impact of these variants, as there was limited data for computational approaches to train the prediction of the functional impact of mtDNA variants. A recent concept of CSC plasticity proposes that cancer cells can shift between non-CSC and CSC states, depending on external signals or their interaction with the neighbouring cells within a tumour niche. This suggests that DNA mutation is not a requisite for tumour cells to acquire stem-like traits^84,85^. Our findings seem to support this theory. The plasticity of SAS cells, rather than DNA mutation, drove their transformation into tumour spheres with CSC features.

**Table 4:** Lists of variants discovered in SAS, SAS tumour spheres, and H103.

Mitochondrial region	Base position	Reference base	Base alteration	Variant allele fraction		
				SAS	SAS tumour spheres	H103
D-loop	73	A	G	0.22	0.31	0.55^a,c^
D-loop	150	C	T			0.52
D-loop	260	G	A	0.30^a^	0.24	
D-loop	263	A	G	0.58	0.51^a^	0.57^a^
D-loop	282	T	C			0.45
D-loop	309	C	CCC	0.10^a^	0.12^a^	
D-loop	315	C	CC			0.40^a^
D-loop	489	T	C	0.41	0.36	
*MT-RNR1*	709	G	A	0.75	0.56^b^	
*MT-RNR1*	750	A	G	0.64	0.52	0.79^a^
*MT-RNR1*	1438	A	G	0.51	0.39	0.53^a^
*MT-RNR2*	1811	A	G			0.44^a^
*MT-RNR2*	2706	A	G	0.64	0.51	0.73^a^
*MT-ND1*	3738	C	T			0.91^a^
*MT-ND1*	4107	C	T	0.23^a^	0.16^a^	
*MT-ND2*	4505	C	T	0.67	0.54	
*MT-ND2*	4769	A	G	0.37	0.38	0.50^a^
*MT-ND2*	4833	A	G	0.42	0.35	
*MT-ND2*	5108	T	C	0.59	0.31^b^	
*MT-ND2*	5240	A	G			0.54^a^
*MT-TA*	5601	C	T	0.34	0.39	
*MT-CO1*	6392	T	C			0.17^a^
*MT-CO1*	6455	C	T			0.55^a^
*MT-CO1*	6737	A	G	0.62	0.54	
*MT-CO1*	7028	C	T	0.68	0.51	0.46^a^
*MT-CO1*	7055	A	G			0.46^a^
*MT-CO2*	7600	G	A	0.63	0.25^b^	
*MT-ATP6*	8701	A	G	0.63	0.57	
*MT-ATP6*	8860	A	G	0.58	0.48	0.68^a^
*MT-ATP6*	9165	T	C	0.75	0.71	
*MT-CO3*	9365	C	T			0.33^a^
*MT-CO3*	9377	A	G	0.58	0.63	
*MT-CO3*	9540	T	C	0.69	0.48^b^	
*MT-CO3*	9575	G	A	0.62	0.56	
*MT-CO3*	9698	T	C			0.67^a^
*MT-ND3*	10398	A	G	0.58	0.53	
*MT-ND3*	10400	C	T	0.63	0.62	
*MT-ND4L*	10733	C	T			0.60^a^
*MT-ND4*	10873	T	C	0.26^a^	0.09^a^	
*MT-ND4*	11465	T	C			0.55^a^
*MT-ND4*	11467	A	G			0.64^a^
*MT-ND4*	11719	G	A	0.73	0.72	0.67^a^
*MT-ND4*	11809	T	C	0.65	0.50	
*MT-TL2*	12308	A	G			0.50^a^
*MT-TL2*	12311	T	C	0.58	0.42	
*MT-ND5*	12372	G	A			0.30^a^
*MT-ND5*	12705	C	T	0.70	0.47^b^	
*MT-ND5*	13145	G	A			0.38^a^
*MT-ND5*	13247	T	C			0.31^a^
*MT-ND5*	13563	A	G	0.64	0.47	
*MT-ND5*	13677	C	T	0.36	0.39	
*MT-ND6*	14200	T	C	0.54	0.56	
*MT-ND6*	14281	C	T	0.31	0.54^a,b^	
*MT-ND6*	14569	G	A	0.59	0.59	
*MT-CYB*	14766	C	T	0.69	0.53	0.64^a^
*MT-CYB*	14783	T	C	0.59	0.42^b^	
*MT-CYB*	14798	T	C	0.51	0.31^b^	
*MT-CYB*	15043	G	A	0.67	0.57	
*MT-CYB*	15301	G	A	0.42	0.32	
*MT-CYB*	15326	A	G	0.76	0.57^b^	0.70
*MT-TT*	15924	A	G	0.60	0.37^b^	
D-loop	16146	A	G			0.27^a^
D-loop	16184	C	A	0.21^a^	0.26a	
D-loop	16186	C	T	0.13^a^	0.26^a,b^	
D-loop	16189	T	C	0.15^a^	0.14^a^	
D-loop	16223	C	T	0.67	0.60	
D-loop	16260	C	T			0.29^a^
D-loop	16269	A	G	0.64	0.37^b^	
D-loop	16278	C	T	0.74	0.65	
D-loop	16342	T	C			0.37
D-loop	16362	T	C	0.44	0.33	

The variant allele fraction was computed based on the fraction of the base-called reads that supported the variant, generated by Nanopolish, and the base statistics from Integrative Genomics Viewer version 2.3.97.

^a^Variant allele fraction was calculated from the base statistics from Integrative Genomics Viewer version 2.3.97, where the minimum allele coverage was set to nine and the minimum number of variant reads was set to three.

^b^Fisher’s exact test for the differences in the variant allele fractions between SAS and SAS tumour spheres. P < 0.05.

^c^Fisher’s exact test for the differences in the variant allele fractions between H103 and SAS. P < 0.05.

### MtDNA D-loop alteration was associated with the mtDNA content and cisplatin response

H103 harboured 32 variants, 24 of which were found in the coding region of the mitochondrial genome, and 8 mutations were found in the D-loop (Table 4). Comparing the mtDNA profiles of SAS and H103, we found a D-loop mutation that was only present in SAS. The mutation involved 1 C or 2 C insertions (C7→C8/C9) in the D310 mononucleotide sequences (position 303–309) of the D-loop. The D-loop is a non-coding region that contains the leading strands for the origin of mtDNA replication, and the promoters for the transcription of mitochondrial genes^86^. Therefore, we deduced that the D310 mutation could alter mtDNA content and thus cisplatin response. Lacking the D310 mutation, H103 was less efficient at replicating its mitochondrial genome than SAS, hence lowering its mtDNA content as shown by our qPCR analysis (Fig. 3). The D310 mutation was previously correlated with an increase in the mtDNA copy number in human laryngeal squamous cell carcinoma^87^. Other works have also reported the importance of the D310 mutation in breast, gallbladder, and colorectal tumourigenesis^88–90^.

### Common deletion of the mitochondrial genomes was not associated in cisplatin responsiveness in OSCC

Aside from point mutations, mtDNA alterations may also involve mtDNA deletions. To date, many mtDNA deletions have been catalogued in MITOMAP (https://www.mitomap.org), a human mitochondrial genome database, and associated with various pathological conditions. Among the reported mtDNA deletions, a common mtDNA deletion (4977-bp deletion between 8470–13459 bp) has been shown to promote the carcinogenesis of hepatocellular carcinoma and colorectal cancer^91,92^. The mtDNA deletion causes the loss of several genes that encode the OXPHOS proteins, namely ATP synthase F_o_ subunits 6 and 8, cytochrome c oxidase III, and NADH dehydrogenase subunits 3, 4, 4 L and 5. This will in turn lead to dysfunction of the cellular energy metabolism^93^. Here, we ascertained the presence of mtDNA deletions in our samples by analysing the nanopore sequences, whose length allows the detection of structural DNA variants. From the analysis, all the samples were found to have intact mtDNA. To confirm this, we then used a PCR-based assay to detect the 4977-bp mtDNA deletion in the SAS and H103 cells, after they had been treated repeatedly with cisplatin. Consistently, the cisplatin treatments were not found to induce mtDNA deletions (Supplementary Figure S1). Prior work has similarly shown that mtDNA was resistant to chemically induced deletions, as damaged mtDNA was presumably excluded during replication^94^. In addition, it has been reported that mtDNA deletions were less common in cancerous tissues than their benign counterparts in breast, gastric, and head and neck cancers^95–97^. Hence, we suggest that large-scale mtDNA deletion is not required for oral cancer development and therefore is not a crucial factor affecting the cisplatin response.

### Enhanced cisplatin resistance in stem cell-like tumour spheres was not influenced by methylation status of the mitochondrial genome

One of the most studied epigenetic modifications in cancer is DNA methylation, which involves the addition of a methyl group to a cytosine base (commonly at cytosine-guanine dinucleotide, CpG) and promotes changes in gene expression without changing the DNA sequences. Much of prior research has focused on the relation between epigenetic changes in nuclear DNA and the development of cancer^98^. Methylation of mtDNA was thought to be absent. However, this has been disproved; methylation of the mitochondrial genomes actually exists, albeit at a low level as compared to the nuclear genomes^99–102^, and it regulates mtDNA replication and transcription^103–105^. Thus, we hypothesised that the epigenetic modifications of mtDNA may influence mtDNA replication, altering mitochondrial function and the cellular response of OSCC to cisplatin. In this study, we used MinION to evaluate the methylation status of the CpG sites in the mitochondrial genomes of SAS, SAS tumour spheres, and H103. The methylation-calling pipeline is described in detail in the Supplementary Notes. In brief, the CpG methylation frequency was computed by Nanopolish and the CpG sites that were differentially methylated between the samples were obtained from MOABS (Model-based Analysis of Bisulphite Sequencing Data). We found that no CpG sites were differentially methylated between SAS and SAS tumour spheres.

### The relation between mtDNA alterations, gene expression profiles, and cisplatin sensitivity

Overall, cisplatin sensitivity exists on a continuum, and a melange of factors may contribute to its variation between tumours. H103 was substantially more cisplatin-resistant than SAS and the tumour spheres. By a series of analyses, we found that the difference probably arose from a constellation of genetic or epigenetic changes in their nuclear and mitochondrial genomes.

Cancer cells may alter mtDNA content to fit their energy demand^66,106^, shifting the expression of mitochondrial genes and thus mitochondrial activities^67,107^. We found that SAS tumour spheres, with considerably less mtDNA, had a lower expression level of *MT-CO2* than SAS (P = 0.0323). Similarly, the expression levels of most of the other mitochondrial genes were slightly lower in SAS tumour spheres than the parent SAS cells (Supplementary Table S4). As described earlier, the microarray analysis revealed that the tumour spheres underwent metabolic reprogramming and preferentially used glycolytic metabolism as an energy source. In cancer, glycolysis is augmented at the expense of mitochondrial activity, resulting in low mtDNA content^67,107^. Such precise control of mitochondrial activity by the nucleus is well known and serves to maintain cellular homeostasis^100,108^. The replication and the transcription of mtDNA are regulated by an assembly of nucleus-encoded proteins at the mitochondrial D-loop, namely DNA polymerase γ, Twinkle DNA helicase, mitochondrial single-stranded DNA binding protein (mtSSB), mitochondrial RNA polymerase (POLRMT), mitochondrial transcription factor A (TFAM), and mitochondrial transcription factors B1 (TFB1M) and B2 (TFB2M)^105,109,110^. The link between metabolic reprogramming of cancer cells and drug resistance has been described in several prior studies. Increased glycolysis was observed in drug-resistant lung cancer, multiple myeloma, and ovarian cancer. Interestingly, blockade of glycolysis killed the drug-resistant cancer cells^111–114^. In this study, it is likely that the metabolic reprogramming of the tumour spheres, in tandem with the resultant effect on the mitochondrial genome, reduced their cisplatin responsiveness.

The cisplatin resistance we observed for H103 was likely caused by a similar relationship between reduced mitochondrial function and aberrant cellular adaptation. We expected H103 to have uniformly lower expression levels of its mitochondrial genes when compared with SAS. However, the microarray analysis showed that only the expression of *MT-ND2* was significantly lower in H103 than SAS (Supplementary Tables S5; P = 0.0163). This suggests that the mitochondrial genes contribute unequally to cisplatin resistance, or *MT-ND2* is most responsive to external signals. The first hypothesis seems most plausible. *MT-ND2* encodes NADH dehydrogenase 2, which is a subunit of mitochondrial complex I and a major gatekeeper of ROS production^115^. Hence, the lower expression level of *MT-ND2* in the H103 cells may explain their reduced capacity to produce ROS, rendering them less sensitive to cisplatin.

Then, we tried to reconcile our findings with the large body of evidence for the complex interplay between DNA methylation, mtDNA replication and transcription, and cisplatin response^116–118^. Recent studies have shown that mtDNA replication and transcription are regulated by methylation at the mitochondrial D-loop^105,109^. However, in this study, we found no differentially methylated CpGs in the D-loops of SAS tumour spheres and H103, when we compared them with SAS. This suggests that the mitochondrial D-loop methylation was not responsible for the variation of mtDNA content and mitochondrial gene expression, and other regulatory mechanisms might be at play. Prior studies have shown that mtDNA replication is epigenetically controlled by the nuclear genome. In particular, global DNA methylation reduces mtDNA content by suppressing the expression of the nucleus-encoded proteins that drive mtDNA replication and transcription^65,116,119^.

Some studies have pointed to a potential role of gene body methylation within mtDNA in regulating the expression of mitochondrial genes, though this is still incompletely elucidated^99,116^. In general, the methylation at promoter sites silences gene expression, but gene body methylation produces an opposite effect and activates gene transcription^98,120–122^. A study on glioblastoma found no direct correlation between gene body mtDNA methylation and gene transcription and suggested that the mtDNA methylation may have an indirect and widespread effect on post-transcriptional events. In the study, the overall mtDNA gene expression was down-regulated although only certain mtDNA gene regions became less methylated following treatments with DNA demethylating agents^116^. In this study, we found that three CpG sites in the mitochondrial *COX1* and *CYTB* genes (*MT-CO1* and *MT-CYB* respectively) of H103 were hypermethylated when compared with both SAS and SAS tumour spheres (Table 5). Through the microarray analysis, we found that H103 had marginally higher expression levels of most of the mitochondrial genes, including *MT-CO1* and *MT-CYB*, than SAS, though the differences were not statistically meaningful (Supplementary Table S5). Hence, we suggest that the mtDNA methylation in the gene bodies promoted the expression of the genes, presumably by affecting the post-transcriptional modifications of polycistronic mitochondrial mRNAs^116,123^. However, the transcription-enhancing effect of the hypermethylation could have been offset by low mtDNA content, curbing any potential increases in gene expression. In addition, we may suggest that the mtDNA methylation differences between H103 and SAS could have arisen from their distinct mitochondrial and nuclear genotypes. The findings of a prior study support this possibility. Changes in mtDNA methylation patterns were observed in the tumour models of glioblastoma and osteosarcoma when their mtDNA or nuclear genotypes were varied^105^.

**Table 5:** Differences in the methylation of the CpG sites in the mitochondrial genomes of SAS and H103, as analysed by MOABS.

CpG position	Gene region	SAS		H103		Credible methylation difference (CDIF)
		Total called sites	Methylated frequency	Total called sites	Methylated frequency	
7160	COX1	16	0.125	8	0.75	0.264
7332	COX1	33	0.0909	9	0.667	0.246
15698	CYTB	30	0.233	6	0.833	0.204

A CpG site was considered differentially methylated between two samples when the credible methylation difference exceeded 0.2.

## Conclusions

In this study, we derived CSCs from SAS OSCC cells using a sphere-forming assay. A combination of flow cytometry, qPCR, Western blot, and microarray analyses showed that the tumour spheres exhibited marked stemness features, namely increased expression levels of stemness genes and proteins, common CSCs surface markers, and genes involved in glucose and lipid metabolism. We found that SAS tumour spheres were more cisplatin-resistant than their parental cells and that they had less mtDNA, which is the therapeutic target of cisplatin. Consistently, we found that mtDNA content was also reduced in another cell line that was similarly cisplatin-resistant to the tumour spheres, namely H103.

Using a novel nanopore sequencer, MinION, we then sequenced their mtDNA. We found that SAS and SAS tumour spheres harboured a D-loop mutation that was absent in H103. The mutation could have altered mtDNA content and therefore cisplatin response. We also found that all the cells had intact mtDNA, suggesting that mtDNA deletion is not one of the factors affecting cisplatin sensitivity. An analysis of mtDNA methylation detected three hypermethylated CpG sites in the *COX1* and *CYTB* genes of H103. We inferred that the reduced cisplatin sensitivity in H103 could have been caused by a variety of converging genetic mechanisms, of which mtDNA alterations are key drivers (low mtDNA content, point mutations and methylation changes), that disrupt mitochondrial function, apoptosis, and cisplatin response. However, how the differences in mtDNA variants between H103 and SAS could have altered protein functions and cisplatin sensitivity has yet to be confirmed. The recent approaches to precise genome editing, using transcription activator-like effector nucleases (TALENs), zinc-finger nucleases (ZFNs), or the CRISPR-Cas9 system, provide new opportunities for understanding the effects of the changes in a cancer genome^124^.

We did not find significant differences in the mtDNA profiles of SAS and the tumour spheres that could have been culpable of the difference in their responses to cisplatin. A possible explanation may lie in the metabolic reprogramming exhibited by CSCs and the interaction between the mitochondria and the nucleus in regulating mtDNA content.

Overall, we suggest that mtDNA alterations might serve as markers of cisplatin responsiveness in OSCC. Future work may aim to investigate the mechanisms that underpin variation in mtDNA and therefore cisplatin response.

## Methods

### Cell lines

Human OSCC cell lines used in this study, namely SAS (poorly differentiated; stage II; Japanese Cell Bank Research) and H103 (well differentiated; stage I; European Collection of Authenticated Cell Cultures), were generous gifts from Professor Leong Chee-Onn, (International Medical University, Malaysia) and Professor Ian Charles Paterson (University of Malaya, Malaysia). SAS was cultured in Dulbecco’s Modified Eagle’s Medium/Ham’s Nutrient Mixture F12 (DMEM/Ham’s F12; Nacalai Tesque Inc., Japan), supplemented with 10% fetal calf serum (FCS; GE Healthcare Life Sciences, USA) and 1% penicillin/streptomycin (Nacalai Tesque Inc., Japan). H103 was cultured in DMEM/Ham’s F12, supplemented with 10% FCS, 1% penicillin/streptomycin, and 0.5 µg/ml sodium hydrocortisone succinate (Sigma-Aldrich, USA). The cells were maintained at 37 °C in 5% CO_2_ humidified air.

### Tumour sphere-forming assay

CSCs were derived from SAS and H103 using a sphere-forming assay. Cells were cultured at a density of 2.5 × 10^4^ cells/ml in a low-attachment 6-well plate (Corning Inc., USA) as tumour spheres in serum-free DMEM/Ham’s F12, supplemented with 1× N-2 supplement (Thermo Fisher Scientific, USA), 1% penicillin/streptomycin, 10 µg/ml heparin sodium salt (Sigma-Aldrich, USA), 20 ng/ml human recombinant basic fibroblast growth factor, and 20 ng/ml epidermal growth factor (Gold Biotechnology Inc., USA) for 7 d. The medium was replenished every other day. The microscopic images of the tumour spheres were taken, and the diameter of each sphere was measured using ImageJ version 1.50i (National Institutes of Health, USA).

### Flow cytometry

The expression of common surface markers for CSCs, namely CD338, CD117, and CD44, was measured via flow cytometry. For the multi-staining flow cytometric assay, single-cell suspensions (1 × 10^6^ cells/100 µl) were incubated with 5 µl of monoclonal phycoerythrin (PE)-conjugated mouse anti-human CD338 (Catalog No. 561180; BD Biosciences, USA) and BB515-conjugated mouse anti-human CD117 (Catalog No. 559925; BD Biosciences, USA) for 30 min on ice. A single-staining flow cytometric assay was performed to analyse the surface expression of CD44, after the cells were incubated with 5 µl of monoclonal PE-Cy7-conjugated mouse anti-human CD44 (Catalog No. 560533; BD Biosciences, USA). 7-amino-actinomycin D (7-AAD; Catalog No. 561180; BD Biosciences, USA) was added to exclude non-viable cells in both assessments. Flow cytometric analyses were carried out using BD FACSCanto II Cell Analyzer and BD FACSDiva Software version 6.1.3 (BD Biosciences, USA).

### Western blotting

The expression levels of stemness proteins were measured by Western blotting. Total proteins were extracted on ice using a RIPA lysis buffer (EMD Millipore, USA), which contained a cocktail of protease inhibitors (Thermo Fisher Scientific, USA), for 30 min. 30 µg of proteins were separated electrophoretically in a 10% sodium dodecyl sulphate–polyacrylamide gel at 150 V for 100 min and were transferred to a nitrocellulose membrane (GE Healthcare Life Sciences, USA) at 100 V for 75 min. The membrane was blocked with 5% non-fat skim milk (Nacalai Tesque Inc., Japan) and incubated overnight at room temperature with primary antibodies against the mouse monoclonal Sox2 antibody (1:1000; Catalog No. GTX627404; GeneTex Inc., USA), the mouse monoclonal Oct4 antibody (1:1000; Catalog No. GTX627419; GeneTex Inc., USA), and the mouse monoclonal beta-actin loading control antibody (1:3000; Catalog No. MA5–15793; Thermo Fisher Scientific, USA). On the next day, the membrane was further incubated with a corresponding horseradish peroxidase-conjugated goat anti-mouse IgG polyclonal secondary antibody (1:3000; Catalog No. GTX213111-01; GeneTex Inc., USA; and Catalog No. 31430; Thermo Fisher Scientific, USA) for 2 h at room temperature. The protein bands were visualised via ChemiDoc XRS + System (Bio-Rad Laboratories Inc., USA) after the addition of an enhanced chemiluminescence substrate containing 0.1 M Tris pH 8.5 (First Base Laboratories Sdn Bhd, Malaysia), 1.25 mM luminol (Sigma-Aldrich, USA), 1.15 mM coumeric acid (Sigma-Aldrich, USA), and 0.192% hydrogen peroxide (Bio Basic Inc., Canada). The data were analysed using Image Lab Software version 5.2.1 (Bio-rad Laboratories Inc., USA). The background-subtracted intensities of the bands were used to quantify the expression levels of the target proteins.

### Cisplatin sensitivity testing

Single-cell suspensions of SAS and H103 were plated in a 96-well normal-attachment plate while SAS tumour spheres were plated in a 96-well low-attachment plate, at a density of 5×10^3^ cells/well and incubated overnight at 37 °C in 5% CO_2_ humidified air. The cells were treated with varied concentrations of cisplatin (5, 10, 20, 30, 60 and 100 µM; TCI America, USA) for 72 h. The viability of the cells following the drug treatment was assessed using CellTiter 96 AQueous Non-Radioactive Cell Proliferation Assay (MTS assay; Promega Inc., USA), according to the manufacturer’s instructions. The absorbance was measured at a wavelength of 490 nm with Infinite 200 PRO microplate reader (Tecan Group Ltd., Switzerland). The control group consisted of untreated cells. The results were expressed as percentages of cell viability compared to the control group. IC_50_ was defined as the concentration of cisplatin required to inhibit cell viability by half.

### Microarray analysis

Total RNA was isolated using innuPREP RNA Mini Kit (Analytik Jena, Germany). The contaminating genomic DNA in the RNA samples was removed by DNase 1 treatment using RapidOut DNA Removal Kit (Thermo Scientific Inc., USA). The concentrations of the RNA samples and their quality, assessed based on the A_260_/A_280_ ratio, were determined via an OPTIZEN NanoQ Microvolume UV/Visible Spectrophotometer (Mecasys Co. Ltd, Korea). The integrity of RNA was evaluated using Agilent 2100 Bioanalyzer (Agilent Technologies Inc., USA). An A_260_/A_280_ ratio of 1.8–2.1 and an RNA integrity number (RIN) greater than 7.0 indicated RNA of acceptable quality for microarray analysis. The purified RNA samples were then submitted to Research Instruments Sdn. Bhd. Malaysia for microarray analysis. In brief, 100 ng of purified RNA from each sample was used to generate amplified and biotinylated sense strand cDNA using GeneChip WT PLUS Reagent Kit (Thermo Fisher Scientific Inc., USA). Hybridization-ready targets also were prepared using the same kit prior to insertion into Clariom S arrays (Affymetric Inc., USA), which contained over 211300 probes for more than 337100 transcripts of >20000 well-annotated human genes. Hybridization, washing, staining and scanning were performed as described by the manufacturer’s protocol using GeneChip Hybridization Oven 645, GeneChip Fluidic Station 450, and GeneChip Scanner 3000 7 G (Thermo Fisher Scientific Inc., USA). Quality control checks and normalization of the raw gene expression data with the Robust Multi-array Average (RMA) algorithm were performed by using a set of R and Bioconductor modules provided in Transcriptome Analysis Console 4.0 software (Affymetric Inc., USA). Differences in gene expression between paired samples were determined by one-way analysis of variance (ANOVA). A gene was considered to be differentially expressed between two samples when the positive or negative fold change exceeded 2 and the p-value adjusted for the false discovery rate was less than 0.05. The lists of differentially expressed genes with a higher fold change cut-off < −10 or >10 were selected for pathway enrichment analysis using Reactome^125^.

### Real-time quantitative polymerase chain reaction (qPCR)

qPCR was performed to validate the microarray data and evaluate the expression of stemness genes in the derived tumour spheres. The same RNA samples used in the microarray assay were converted into cDNA using SensiFAST cDNA Synthesis Kit (Bioline, Australia). qPCR was performed on a CFX Connect Real-Time PCR Detection System (Bio-rad Laboratories Inc., USA) using SensiFAST SYBR No-ROX Kit (Bioline, Australia) with a three-step thermal cycling protocol, which consisted of an initial denaturation step of 95 °C for 2 min, followed by 40 cycles of 95 °C for 5 sec, 59 °C for 10 sec, and 72 °C for 20 sec. Post-amplification melting curves were analysed to evaluate the reaction specificity and the presence of primer-dimers. The gene expression levels of six genes, namely serine palmitoyltransferase small subunit B (*SPTSSB*), C-C motif chemokine ligand 2 (*CCL2*), microsomal glutathione S-transferase 1 (*MGST1*), Dickkopf WNT signaling pathway inhibitor 1 (*DKK1*), sex-determining region Y-box 2 (*SOX2*), and octamer-binding transcription factor 4 (*OCT4*), were normalised to those of two reference genes, namely glyceraldehyde 3-phosphate dehydrogenase (*GAPDH*) and β-actin (*ACTB*). No-template (NTC) and no-reverse transcriptase (NRT) controls were included in every qPCR run. The sequences of the primers (Integrated DNA Technologies Inc., USA) are provided in Supplementary Table S6.

### MtDNA gene-specific qPCR

qPCR was performed to determine intersample differences in mtDNA content according to a published protocol^126^. Briefly, total DNA from confluent cultured cells was extracted using DNeasy Blood & Tissue kit (QIAGEN, Germany) according to the manufacturer’s instructions. The DNA concentrations and their purity based on the ratio of absorbance at 260 nm and 280 nm were determined via the OPTIZEN NanoQ Microvolume UV/Visible Spectrophotometer (Mecasys Co. Ltd, Korea). The A_260_/A_280_ ratios varied from 1.8 to 2.1, indicating that the samples were pure. qPCR was performed to amplify two mitochondrial genes, tRNA^Leu(UUR)^ and 16 S rRNA, and a nuclear gene, β2-microglobulin (β2M). The cycling protocol consisted of an initial denaturation step of 95 °C for 3 min, followed by 35 cycles of 95 °C for 5 sec, 62 °C for 10 sec, and 72 °C for 20 sec and concluded with melting curve analysis. The qPCR was performed on the CFX Connect Real-Time PCR Detection System (Bio-rad Laboratories Inc., USA) using SensiFAST SYBR No-ROX Kit (Bioline, Australia). The mtDNA content was calculated using the Eq. (1), where ∆Cq is the difference in Cq values between mtDNA (tRNA^Leu(UUR)^ or 16 S rRNA) and β2M genes. The sequences of the primers (Integrated DNA Technologies Inc., USA) are provided in Supplementary Table S7.

1 $$MtDNA\,content=2\times {2}^{-\Delta Cq}$$

### MtDNA sequencing

MtDNA was extracted from 10–15 millions of cells using QIAprep Miniprep Kit (QIAGEN, Germany) according to a published protocol^80^. Further DNA purification was performed using a solid-phase reversible immobilization paramagnetic bead technique with Agencourt AMPure XP (Beckman Coulter Inc., USA). The DNA concentrations and their purity based on the ratio of absorbance at 260 nm and 280 nm were determined via OPTIZEN NanoQ Microvolume UV/Visible Spectrophotometer (Mecasys Co. Ltd, Korea). The A_260_/A_280_ ratios varied from 1.8 to 2.1, indicating that the samples were pure.

Six MinION sequencing runs were performed using two MinION SpotOn Flow Cells version R9.5 (FLO-MIN107; Oxford Nanopore Technologies, UK). Both PCR amplicons and native DNA of each sample were used as the sequencing input. The details of the sequencing runs are described in Table 2. Two ~8-kb products, which span the entire mitochondrial genome (~16 kb), were amplified with the following cycling protocol: an initial denaturation step of 94 °C for 2 min, 35 cycles of 94 °C for 12 sec, 62 °C for 30 sec, and 68 °C for 9 min, and final extension for 7 min at 68 °C. The long PCR was performed using an AtMax Taq DNA Polymerase (Vivantis Technologies Sdn. Bhd., Malaysia) on an Arktik Thermal Cycler (Thermo Scientific Inc., USA). For barcoded PCR samples, the DNA samples were amplified using a previously described protocol using the same two sets of primers with universal sequences. All PCR primers were synthesised by Integrated DNA Technologies Inc., USA and are listed in Supplementary Table S8. The native DNA samples were digested by *Bam*HI (Vivantis Technologies Sdn. Bhd., Malaysia) to linearise the circular mitochondrial genome.

The DNA libraries were prepared using the Ligation Sequencing Kit 1D (SQK-LSK108; Oxford Nanopore Technologies, UK) according to the manufacturer’s instructions. For barcoded PCR samples, the sequencing libraries were prepared using the Ligation Sequencing Kit 1D and the PCR Barcoding Kit (EXP-PBC001; Oxford Nanopore Technologies, UK). Briefly, the end-repair, dA-tailing, sequencing adapter ligation, and final purification of DNA libraries were performed according to the manufacturer’s instructions. The MinION SpotON Flow Cell was primed and the DNA library was loaded according to the manufacturer’s instructions. The duration of all sequencing runs was set to 48 h.

### MinION sequencing output processing

HDF5 raw data were acquired by MinKNOW version 1.7 (Oxford Nanopore Technologies, UK), and local base-calling with demultiplexing setting was performed using Albacore version 2.3.4 (Oxford Nanopore Technologies, UK). The quality of the sequencing raw data was assessed using NanoStat^127^. The data was indexed using Nanopolish^128^ to link each sequencing read with its signal-level data in the HDF5 files. The base-called and indexed sequencing reads were aligned to the human reference genome assembly GRCh38 using BWA-MEM^129^ with the ont2d mode. SAMtools^130^ was used to sort and index the aligned sequencing reads prior to variant- and methylation-calling via Nanopolish. The pipelines for the variant- and methylation-calling are described in more detail in the Supplementary Notes. The functional effect of the variants were predicted using open-source algorithms including PolyPhen-2^131^, PANTHER^132^, Envision^133^, MutationAssessor^134^, MutPred2^135^ and SNPs&GO^136^. The mtDNA deletion was also determined via MitoDel^137^ and eKLIPse^138^. MitoDel is a tool for detecting and quantifying mtDNA deletions even at low heteroplasmy levels via the BLAT split read mapping method. The eKLIPse pipeline uses soft clipping alignment analysis of sequencing reads to predict mtDNA deletions.

### Sanger sequencing

The mutations identified by MinION were cross-checked with Sanger sequencing. 24 pairs of primers (Supplementary Table S9) were used to amplify overlapping PCR products that spanned the whole mitochondrial genome, using MyTaq Mix (Bioline, Australia). The products varied in size from 767 to 1079 bases. Touchdown PCR was performed on the CFX Connect Real-Time PCR Detection System (Bio-rad Laboratories Inc., USA) to obtain PCR products with high specificity. A two-phase cycling protocol was used. The first PCR phase consisted of an initial denaturation step of 95 °C for 3 min, followed by 10 cycles of gradually decreasing the annealing temperature from 68 °C to a target temperature of 58 °C; the second phase continued with 25 cycles of 95 °C for 5 sec, 58 °C for 10 sec, and 72 °C for 1 min. The PCR products were then Sanger-sequenced using the BigDye Terminator v3.1 Cycle Sequencing Kit chemistry (Thermo Scientific Inc., USA) on an Applied Biosystems Genetic Analyzer (Thermo Scientific Inc., USA; outsourced to First BASE Laboratories Sdn. Bhd. Malaysia). The resultant mtDNA sequences were visualized and analysed using Geneious version 10.2.3 (Biomatters Ltd., Auckland, New Zealand).

### Nested-PCR

A common 4977-bp mtDNA deletion was detected using a previously reported nested PCR assay^91,92^. We chose nested PCR because it can detect low-abundance mtDNA deletion by two successive rounds of amplification. The reaction mix for the first round of PCR consisted of 5 ng/µl of total DNA, 0.2 µM each of the forward and reverse PCR primers, and 1× MyTaq Mix (Bioline, Australia). The cycling protocol consisted of initial denaturation at 95 °C for 1 min, followed by 15 cycles of 95 °C for 15 sec, 62 °C for 15 sec, and 72 °C for 10 sec. A second round of PCR was performed using the first PCR product (diluted 1:100) and a different set of primers, amplifying a smaller fragment in 25 cycles of PCR. The PCR protocol was otherwise identical to what we used for the first round of PCR. A synthetic 595-bp DNA fragment was used as a positive control. The DNA fragment comprised the sequences that flank the 4977-bp deletion, which is known to occur between two 13-bp repeats in the mitochondrial genome, namely 8470–8482 bp and 13447–13459 bp. The sequences of the primers and the DNA fragment (Integrated DNA Technologies Inc., USA) are provided in Supplementary Table S10. The presence of the mtDNA deletion would be indicated by the amplification of a 358-bp product. When the mtDNA deletion was absent, no products would be obtained, as the short extension time would not allow the PCR to amplify the large interposing region between the 13-bp repeats (>5 kb).

### Intracellular reactive oxygen species assay

The generation of intracellular reactive oxygen species (ROS) in cells treated with cisplatin was measured via fluorescence microplate-based analysis following staining with 2′, 7′-dichlorodihydrofluorescein diacetate (H_2_DCFDA; Sigma Aldrich Inc., USA). Briefly, cells were seeded in 12-well plates, at a density of 5 × 10^3^ cells/well, and incubated overnight at 37 °C in 5% CO_2_ humidified air. Cells were then treated with their respective IC_50_ doses of cisplatin for 72 h and 100 µM of H_2_O_2_ for 1 h. The cells were harvested by trypsinization and washed using serum-free media. In parallel, the cells were subjected to incubation with 10 µM H_2_DCFDA for 30 min at 37 °C for ROS staining and cell viability determination. After H_2_DCFDA staining, the excess dye was removed via centrifugation. The cells were then transferred into a black 96-well plate, and the fluorescence intensity (excitation: 504 nm; emission: 529 nm) was measured using Varioskan Flash (Thermo Scientific Inc., USA). Changes in intracellular ROS production relative to a control were obtained after the fluorescence intensity (F) was normalized to the absorbance (Abs) values from the cell viability assay using the following Eq. (2);

2 $$Relative\,Fluorescence\,Intensity=\frac{({F}_{Treatment}-{F}_{Blank})/(Ab{s}_{Treatment}-Ab{s}_{Blank})}{({F}_{Control}-{F}_{Blank})/(Ab{s}_{Control}-Ab{s}_{Blank})}$$

where treatment, control, and blank represent cisplatin- or H_2_O_2_-treated cells, untreated cells, and solvent alone, respectively.

### Statistical analysis

All data are presented as means and standard deviations of three independent experiments and were statistically analysed using the Student’s t-test (Western blotting, flow cytometry, IC_50_ value, and qPCR), Fisher’s exact test (variant allele fraction), and one-way ANOVA (mtDNA gene-specific qPCR and intracellular ROS assay). All the statistical analyses were performed using GraphPad Prism version 7 (GraphPad Software, Inc., USA). Differences between groups were considered statistically significant when P < 0.05.

## Supplementary information

Supplementary Information.

Dataset S1.

Dataset S2.

Dataset S3.

Dataset S4.

Dataset S5.

Dataset S6.

Dataset S7.

Dataset S8.

## Data Availability

The raw sequencing data generated by MinKNOW were deposited in Sequence Read Archive (SRA; Accession No.: PRJNA712949). The raw data from the microarray analysis were deposited in Gene Expression Omnibus (GEO; Accession No.: GSE168424). The raw data for the other analyses are provided as supplementary datasets.
