# Author Correction: Mitochondrial DNA alterations may influence the cisplatin responsiveness of oral squamous cell carcinoma

**DOI:** 10.1038/s41598-021-93444-w

**Published:** 2021-07-05

**Authors:** Amnani Aminuddin, Pei Yuen Ng, Chee-Onn Leong, Eng Wee Chua

**Affiliations:** 1 Drug and Herbal Research Centre, Faculty of Pharmacy, Universiti Kebangsaan Malaysia, Jalan Raja Muda Abdul Aziz, 50300 Kuala Lumpur, Malaysia; 2 School of Pharmacy, International Medical University, Bukit Jalil, 57000 Kuala Lumpur, Malaysia; 3 Centre for Cancer and Stem Cell Research, Institute for Research, Development and Innovation, International Medical University, Bukit Jalil, 57000 Kuala Lumpur, Malaysia

Correction to *Scientific Reports* 10.1038/s41598-020-64664-3, published online 12 May 2020

The original version of this Article contained errors in the qPCR analysis of the gene expression levels and mitochondrial DNA content. The calculations were based on the incorrect assumption that the amplification efficiency was 100%. The Authors have now reanalysed the data using the actual values for amplification efficiency (estimated using the serial dilution method), and found that the p-values marked by asterisks in panel (b) of Figure 2 and Figure 3 and described in the figure legends were incorrect.

The description of the statistical significance annotations in these figures has been corrected in the figure legends, where in the legend of Figure 2,

“Data are presented as mean ± SD. *P < 0.05, ***P < 0.001, ****P < 0.0001, n = 3.”

now reads:

“Data are presented as mean ± SD. *P < 0.05, **P < 0.01, ***P < 0.001, n = 3.”

In the legend of Figure 3,

“Data are presented as mean ± SD. **P < 0.01, ***P < 0.001, n = 3.”

now reads:

“Data are presented as mean ± SD. **P < 0.01, n = 3.”

Figures 2 and 3 and their accompanying legends have been corrected in the published Article, the original versions appear below for reference.

**Figure 2:**
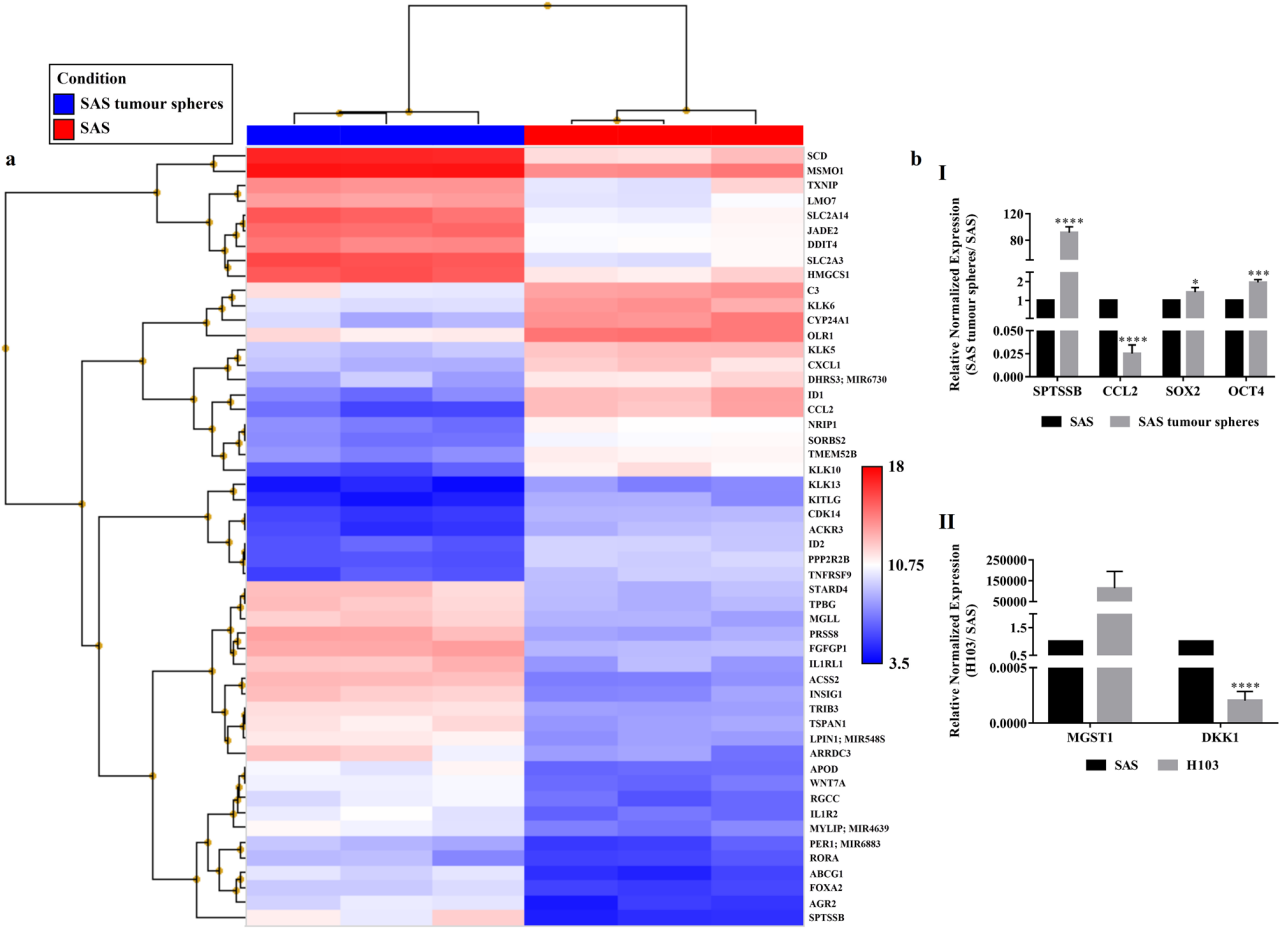
The transcriptomic profiles of SAS cells and their derived tumour spheres as analysed using the Affymetrix Clariom S arrays. (**a**) Heat map generated from the microarray data reflecting log2 normalised gene expression values using the Robust Multi-array Average method, where the p-value adjusted for the false discovery rate was less than 0.05 and the positive or negative fold change exceeded 10. Blue represents lower gene expression and red represents higher gene expression. *n* = 3. (**b**) Microarray validation through qPCR for the top up- or down-regulated genes in (I) SAS tumour spheres and (II) H103 relative to SAS. Expression of stemness-associated genes, *OCT4* and *SOX2*, were also measured by qPCR in SAS and SAS tumour spheres. The amplification levels of the genes were normalised against two reference genes, *ACTB* and *GAPDH*. Data are presented as mean ± SD. *P < 0.05, ***P < 0.001, ****P < 0.0001, *n* = 3.

**Figure 3:**
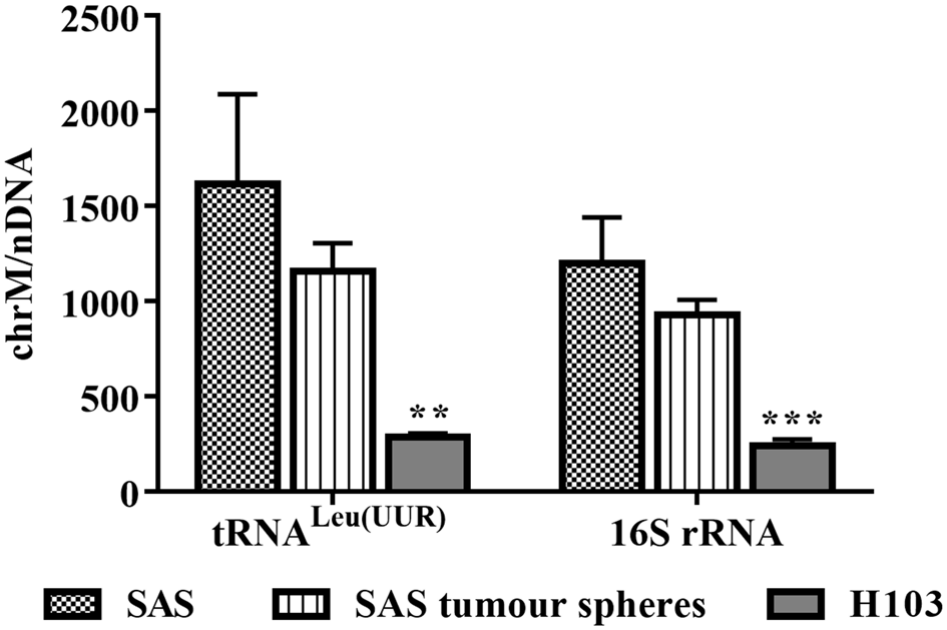
qPCR estimation of mtDNA content. The amplification levels of two mitochondrial genes, tRNA^Leu(UUR)^ and 16S rRNA, were normalised against that of a nuclear gene, β2-microglobulin. Data are presented as mean ± SD. **P < 0.01, ***P < 0.001, *n* = 3.

Additionally, in the Results and discussion section under the subheading ‘SAS tumour spheres with stem cell-like features showed increased expressions of metabolism-associated and pluripotency genes.’,

“The increased expression levels of two of the genes, namely *OCT4* (P = 0.03) and *SOX2* (P = 0.0004), and the proteins they encode (Oct4, P = 0.004; Sox2, P = 0.17) were confirmed by qPCR (Fig. 2b) and Western blotting (Fig. 1c).”

now reads:

“The increased expression levels of two of the genes, namely *OCT4* (P = 0.016) and *SOX2* (P = 0.052), and the proteins they encode (Oct4, P = 0.004; Sox2, P = 0.17) were confirmed by qPCR (Fig. 2b) and Western blotting (Fig. 1c).”

Furthermore, the Data availability statement was incomplete, where

“Some of the raw data has been provided as supplementary datasets.”

now reads:

“The raw sequencing data generated by MinKNOW were deposited in Sequence Read Archive (SRA; Accession No.: PRJNA712949). The raw data from the microarray analysis were deposited in Gene Expression Omnibus (GEO; Accession No.: GSE168424). The raw data for the other analyses are provided as supplementary datasets.”

Finally, Supplementary Tables S6 and S7 and Supplementary Datasets S4 and S6 have been corrected to include the values for amplification efficiency.

The original Supplementary Information file which includes Tables S6 and S7, and the original Supplementary Datasets S4 and S6 are provided below.

The original Article has been corrected.

## Supplementary Information

Supplementary Information.

Dataset S4.

Dataset S6.

