# Supplementary material for: Mitochondrial DNA alterations may influence the cisplatin responsiveness of oral squamous cell carcinoma
Source: Sci Rep. 2020 May 12;10:7885. doi: 10.1038/s41598-020-64664-3 (PMC7217862; doi:10.1038/s41598-020-64664-3)
Supplement: Supplementary file 1 — Supplementary Information. [file 41598_2020_64664_MOESM1_ESM.pdf]

Mitochondrial DNA alterations may influence the cisplatin responsiveness of oral squamous cell carcinoma

Amnani Aminuddin[1], Pei Yuen Ng[1], Chee-Onn Leong[2,3], & Eng Wee Chua[1,*]

[1]*Drug and Herbal Research Centre, Faculty of Pharmacy, Universiti Kebangsaan Malaysia, Kuala Lumpur, 50300, Kuala Lumpur, Malaysia*

[2]*School of Pharmacy, International Medical University, Bukit Jalil, 57000, Kuala Lumpur, Malaysia*

[3]*Centre for Cancer and Stem Cell Research, Institute for Research, Development and Innovation, International Medical University, Bukit Jalil, 57000, Kuala Lumpur, Malaysia*

[*] Correspondence to: Eng Wee Chua; cew85911@ukm.edu.my

Supplementary Notes

Variant-calling pipeline

The pipeline included read pre-processing, alignment, post-processing and filtering, variant calling, and hard-filtering. We evaluated the accuracy of the pipeline by cross-checking variants called by MinION with Sanger sequencing. The MinION sequencing data was processed using Albacore, BWA-MEM, SAMTools, and Nanopolish as described in the Method section. Albacore allows all reads to be base-called regardless of the final quality score of the reads, enabling us to evaluate the quality of the raw data[1]. We first examined the effect of using different threshold mapping quality scores on variant-calling accuracy. Mapping quality scores are typically used to filter out sequence reads that are misaligned to a reference genome. By default, variants were called from the aligned reads by Nanopolish when the depth of variant was at least 20 and the variant frequency was at least 0.2. Also, we applied several hard-filters to the call sets. Variants with an allele depth score <20.0 and a quality by depth score <2.0 were discarded. Finally, we cross-checked the filtered call set with Sanger sequencing and calculated the false- and true-positive rates. We found that increasing the cut-off beyond the default value (MAPQ30) set by Nanopolish did not improve variant calling accuracy (Supplementary Table S3). We listed the variants identified by Nanopolish and Sanger sequencing in Table 4. We found that Nanopolish missed several deletions or insertions in a homopolymeric cytosine stretch (C-stretch) between nucleotides 303-309 within the D-loop, also known as the D310 mononucleotide repeat. Nevertheless, we confirmed the presence of one of the mutations in the D310 region by inspecting the sequence alignment in Integrative Genome Viewer and Geneious. We speculate that the other variants were missed by MinION sequencing data owing to either a low depth of coverage of (A73G substitution) or sequencing errors (C4107T substitution, T10873C substitution, and 16184–16193 C-stretch mutation).

Methylation-calling pipeline

The ionic signals from MinION were first base-called using Albacore. The reads were then aligned to the human reference genome assembly GRCh38 using BWA-MEM in the ont2d mode, followed by sorting and indexing of the mapped sequencing reads using SAMTools. Nanopolish uses a variable-order hidden Markov model to distinguish the ionic signals produced by methylated and unmethylated CpG sites. We limited the methylation calling to the mitochondrial region. The pipeline was initiated when Nanopolish examined a sequence alignment to extract the positions of the potential CpG sites within a single read. We then checked whether the entire mitochondrial genome was adequately covered by inspecting the sequence alignment in Geneious. Nanopolish then calculated the probability of a CpG site (where the Markov model considered adjacent CpG sites within 10 bp of each other as a group) being methylated or unmethylated by comparing its ionic signal with those of known unmethylated sites[2,3]. A log-likelihood-methylated value and a log-likelihood-unmethylated value were computed, and a positive difference between them indicated methylation. It then proceeded to process the other reads that covered the CpG site. The reads with a log-likelihood difference of <2.5 were discarded, as they were deemed ambiguous. After all the reads were processed, Nanopolish computed a methylation frequency for the CpG site. Subsequently, we used MOABS (Model-based Analysis of Bisulphite Sequencing Data) to identify the CpG sites that were differentially methylated between the samples. The model takes into account both sampling and biological variations and thus suits the assessment of our samples with variable sequencing depths. It uses a beta-binomial hierarchical model to compute a 'credible methylation difference' based on the sequencing depth and the sample reproducibility for both biological significance and statistical power[4]. Therefore, the algorithm circumvents the need for deep sequencing to obtain adequate coverage. Further, it gauges the biological significance rather than the statistical significance of a methylation difference by measuring its magnitude based on the sequencing depth. A CpG site is considered differentially methylated between two samples (both should be adequately covered with a minimum read depth of 3) when the credible methylation difference exceeds 0.2. The CpG methylation differences within the mitochondrial genomes of SAS and H103 are presented in Table 5.

References

1. Tyler, A. D. *et al.* Evaluation of Oxford Nanopore ' s MinION Sequencing Device for Microbial Whole Genome Sequencing Applications. *Sci. Rep.* 1–12 (2018). doi:10.1038/s41598-018-29334-5
2. Simpson, J. T. *et al.* Detecting DNA cytosine methylation using nanopore sequencing. *Nat. Methods* 1–7 (2017). doi:10.1038/nmeth.4184
3. Simpson, J. T. *et al.* Detecting DNA methylation using the oxford nanopore technologies MinION sequencer. *bioRxiv* 047142 (2016).
4. Sun, D. *et al.* MOABS: model based analysis of bisulfite sequencing data. *Genome Biol.* **15,** 1–12 (2014).

Supplementary figure and table legends

Supplementary Figure S1. Representative output image of electrophoresed nested-PCR products on 2% agarose gel for detecting mtDNA deletion. Cisplatin-resistant cells derived from OSCC SAS and H103 cell lines were generated by repeated treatments with low dose of cisplatin (10% of maximal inhibitory concentration, IC_{10}) followed by a stepwise dose escalation for over 4 months. The cells were maintained in drug-free medium to allow recovery of cells prior subsequent treatment. The enhanced resistance capacity of the cells were evaluated using CellTiter 96 AQueous Non-Radioactive Cell Proliferation Assay (MTS). The half maximal inhibitory concentration (IC_{50}) of cisplatin for the derived resistant cells were compared to their parental cells after 72 h treatment with varied doses of cisplatin. The IC_{50} finding demonstrated an increase in resistance capacity of the derived cisplatin-resistant SAS (IC_{50} = 7.44 µM) and H103 (IC_{50} = 30.51 µM) with 1.99- and 1.52-fold higher than of their parental cells respectively. The positive control of the assay was the synthesized 595-bp DNA fragment with the designed sequences that flanked on 4977-bp deletion region between two 13-bp repeats in mitochondrial genome (8470-8482 and 13447-13459 bp). The resultant 358-bp product band indicated the presence of mtDNA deletion. Human genomic DNA (Catalog No. G3041; Promega Corporation, USA) isolated from whole blood from multiple anonymous donors was used as negative control.

Supplementary Figure S2. Raw outputs of Oct4 and Sox2 proteins with their respective internal control β-actin protein from Western blotting. n = 3.

Supplementary Table S1. Lists of differentially expressed genes (DEGs) between SAS tumour spheres and SAS with the negative or positive fold change exceeding 10 and the false discovery rate (FDR) p-value was less than 0.05, assessed by microarray assay. Robust multi-array average (RMA) method was used to generate the expression levels, where the intensities were background corrected, quantile normalized and log2 transformed. FDR is defined as the proportion of false positive among the declared DEGs.

Supplementary Table S2. The numbers and fractions of reads with a quality score >5 or >10, as assessed via NanoStat.

Supplementary Table S3. Assessment of variant-calling accuracy using PCR-amplified SAS tumour spheres sequencing data.

Supplementary Table S4. Mitochondrial gene expression profiles of SAS tumour spheres compared to SAS via microarray analysis. RMA method was used to generate the expression levels, where the intensities were background corrected, quantile normalized and log2 transformed. FDR is defined as the proportion of false positive among the declared DEGs.

Supplementary Table S5. Mitochondrial gene expression profiles of H103 compared to SAS via microarray analysis. RMA method was used to generate the expression levels, where the intensities were background corrected, quantile normalized and log2 transformed. FDR is defined as the proportion of false positive among the declared DEGs.

Supplementary Table S6. List of qPCR primer sequences for microarray validation.

Supplementary Table S7. List of mtDNA gene-specific qPCR primer sequences.

Supplementary Table S8. List of mtDNA PCR primer sequences (MinION sequencing).

Supplementary Table S9. PCR primers used to generate overlapping amplicons for Sanger sequencing of the mitochondrial genome.

Supplementary Table S10. List of nested-PCR primers and the 595-bp DNA fragment sequences.

Supplementary figures

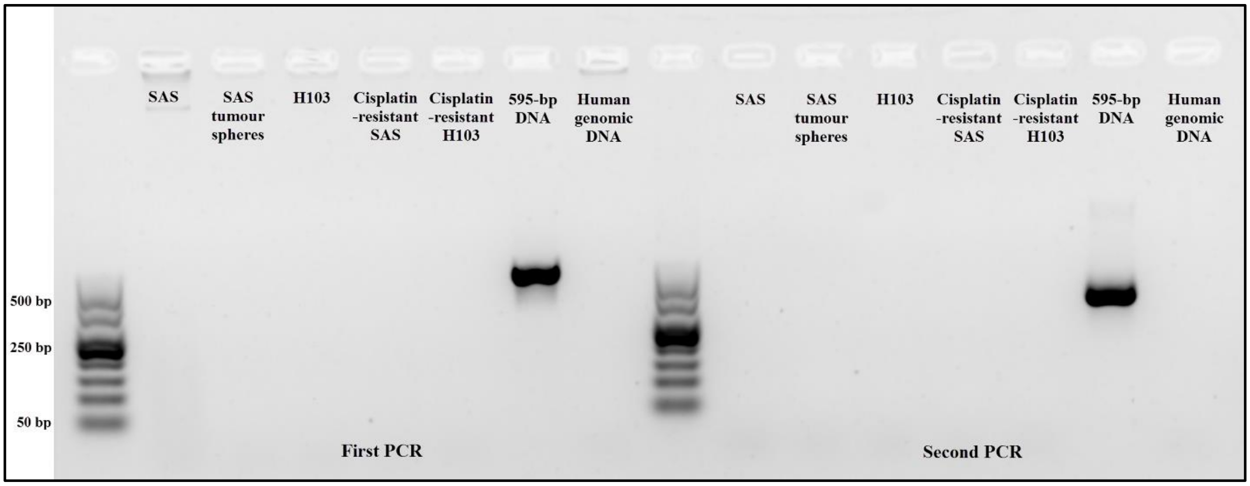

Supplementary Figure S1. Representative output image of electrophoresed nested-PCR products on 2% agarose gel for detecting mtDNA deletion. Cisplatin-resistant cells derived from OSCC SAS and H103 cell lines were generated by repeated treatments with low dose of cisplatin (10% of maximal inhibitory concentration, IC_{10}) followed by a stepwise dose escalation for over 4 months. The cells were maintained in drug-free medium to allow recovery of cells prior subsequent treatment. The enhanced resistance capacity of the cells were evaluated using CellTiter 96 AQueous Non-Radioactive Cell Proliferation Assay (MTS). The half maximal inhibitory concentration (IC_{50}) of cisplatin for the derived resistant cells were compared to their parental cells after 72 h treatment with varied doses of cisplatin. The IC_{50} finding demonstrated an increase in resistance capacity of the derived cisplatin-resistant SAS (IC_{50} = 7.44 µM) and H103 (IC_{50} = 30.51 µM) with 1.99- and 1.52-fold higher than of their parental cells respectively. The positive control of the assay was the synthesized 595-bp DNA fragment with the designed sequences that flanked on 4977-bp deletion region between two 13-bp repeats in mitochondrial genome (8470-8482 and 13447-13459 bp). The resultant 358-bp product band indicated the presence of mtDNA deletion. Human genomic DNA (Catalog No. G3041; Promega Corporation, USA) isolated from whole blood from multiple anonymous donors was used as negative control.

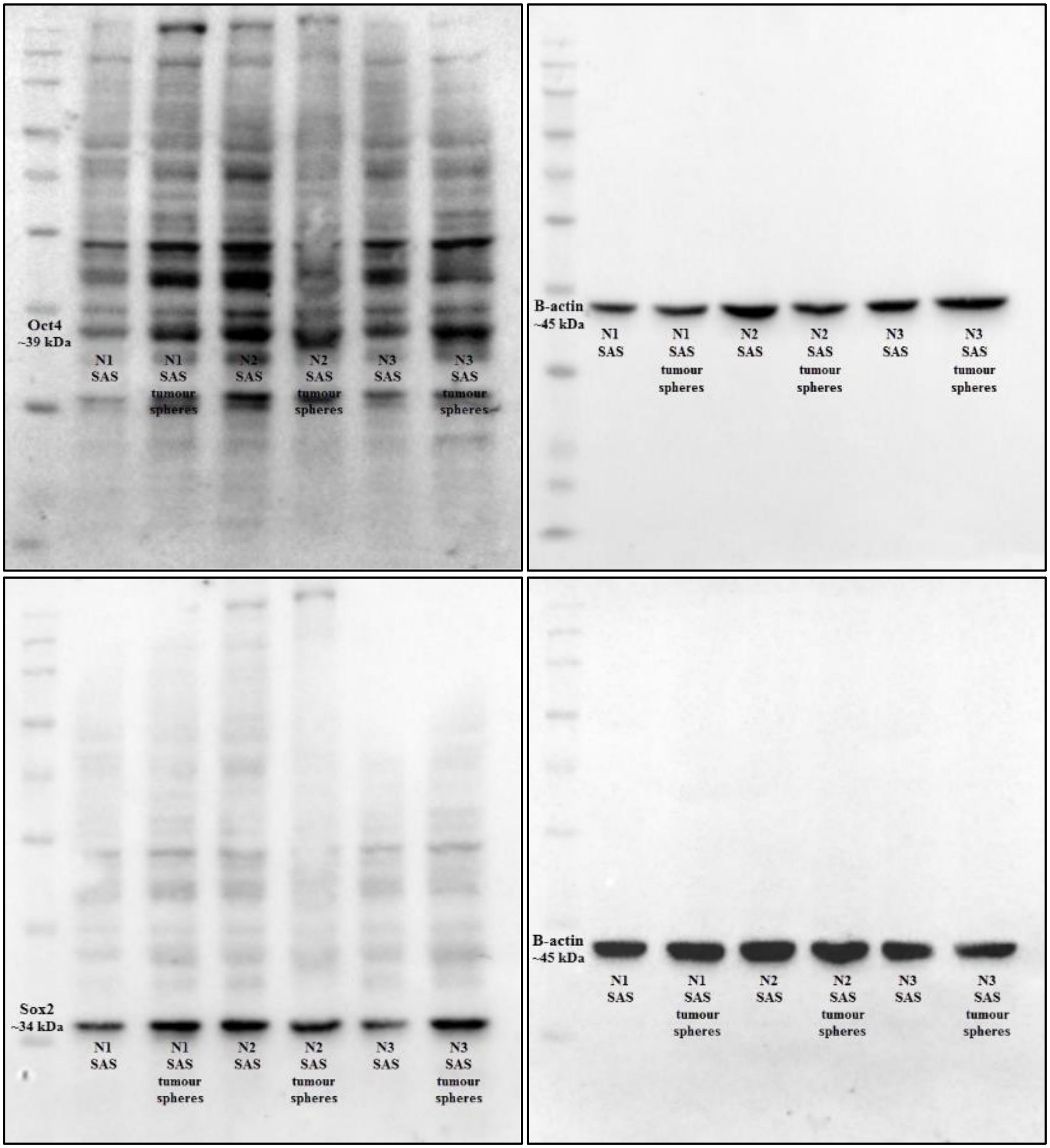

Supplementary Figure S2. Raw outputs of Oct4 and Sox2 proteins with their respective internal control β-actin protein from Western blotting. *n* = 3.

Supplementary Tables

Supplementary Table S1. Lists of differentially expressed genes (DEGs) between SAS tumour spheres and SAS with the negative or positive fold change exceeding 10 and the false discovery rate (FDR) p-value was less than 0.05, assessed by microarray assay. Robust multi-array average (RMA) method was used to generate the expression levels, where the intensities were background corrected, quantile normalized and log2 transformed. FDR is defined as the proportion of false positive among the declared DEGs.

**Probe ID**	**Encoded protein**	**Gene symbol**	**Intensity average (log2)**		**Fold change**	**FDR p-value**
			**SAS tumour spheres**	**SAS**		
TC0300012980.hg.1	Serine palmitoyltransferase, small subunit B	*SPTSSB*	11.2	4.81	83.61	0.0012
TC1200009800.hg.1	Solute carrier family 2 (facilitated glucose transporter), member 3	*SLC2A3*	15.72	9.84	59.16	0.0012
TC2000007202.hg.1	Acyl-CoA synthetase short-chain family member 2	*ACSS2*	12.75	7.1	50.18	0.0002
TC1000008643.hg.1	Stearoyl-CoA desaturase (delta-9-desaturase)	*SCD*	16.98	11.69	38.94	0.0006
TC1600010006.hg.1	Protease, serine, 8	*PRSS8*	13.42	8.2	37.3	0.0005
TC2100007241.hg.1	ATP binding cassette subfamily G member 1	*ABCG1*	10.02	4.88	35.32	0.0006
TC0700010355.hg.1	Anterior gradient 2, protein disulphide isomerase family member	*AGR2*	10.04	5.01	32.75	0.001
TC1200009796.hg.1	Solute carrier family 2 (facilitated glucose transporter), member 14	*SLC2A14*	15.22	10.35	29.15	0.0021
TC0700009807.hg.1	Insulin induced gene 1	*INSIG1*	12.15	7.32	28.54	0.0008
TC0200016501.hg.1	Interleukin 1 receptor-like 1	*IL1RL1*	12.36	7.8	23.54	0.0031
TC0400010125.hg.1	Fibroblast growth factor binding protein 1	*FGFBP1*	13.21	8.83	20.88	0.0004

TC0500008698.hg.1	Jade family PHD finger 2	*JADE2*	14.99	10.67	19.96	0.0002
TC0500011448.hg.1	Arrestin domain containing 3	*ARRDC3*	12.26	8.09	17.99	0.0261
TC0500010635.hg.1	3-hydroxy-3-methylglutaryl-CoA synthase 1 (soluble)	*HMGCS1*	15.49	11.47	16.22	0.001
TC0300013645.hg.1	Apolipoprotein D	*APOD*	10.53	6.55	15.82	0.0012
TC2000008636.hg.1	Forkhead box A2	*FOXA2*	9.23	5.38	14.42	0.0012
TC0100015598.hg.1	Thioredoxin interacting protein	*TXNIP*	13.82	9.97	14.39	0.0245
TC1300006979.hg.1	Regulator of cell cycle	*RGCC*	10.21	6.41	13.96	0.0019
TC0500011702.hg.1	StAR-related lipid transfer domain containing 4	*STARD4*	12.62	8.84	13.77	0.0015
TC0300010350.hg.1	Wingless-type MMTV integration site family, member 7A	*WNT7A*	10.35	6.6	13.48	0.0006
TC0200008663.hg.1	Interleukin 1 receptor, type II	*IL1R2*	10.25	6.52	13.3	0.0038
TC2000006444.hg.1	Tribbles pseudokinase 3	*TRIB3*	11.7	8.02	12.75	0.0004
TC1300009980.hg.1	LIM domain 7	*LMO7*	13.51	9.95	11.85	0.0012
TC0200016403.hg.1	Lipin 1; MicroRNA 548s	*LPIN1; MIR548S*	11.39	7.89	11.29	0.0006
TC0100008145.hg.1	Tetraspanin 1	*TSPAN1*	11.56	8.06	11.26	0.0006
TC0600008655.hg.1	Trophoblast glycoprotein	*TPBG*	12.29	8.8	11.23	0.0007
TC0300012323.hg.1	Monoglyceride lipase	*MGLL*	12.06	8.58	11.15	0.0006
TC1700012353.hg.1	Period circadian clock 1; MicroRNA 6883	*PER1; MIR6883*	8.67	5.2	11.11	0.0051
TC1500009641.hg.1	RAR-related orphan receptor A	*RORA*	8.85	5.39	10.98	0.0228
TC0400009221.hg.1	Methylsterol monooxygenase 1	*MSMO1*	17.49	14.12	10.32	0.0005
TC1000007990.hg.1	DNA damage inducible transcript 4	*DDIT4*	14.17	10.82	10.25	0.0012

TC0600007060.hg.1	Myosin regulatory light chain interacting protein; MicroRNA 4639	*MYLIP; MIR4639*	10.42	7.08	10.11	0.0036
TC0100012921.hg.1	Dehydrogenase/reductase (SDR family) member 3; MicroRNA 6730	*DHRS3; MIR6730*	8.05	11.38	-10.06	0.0066
TC1900009443.hg.1	Complement component 3	*C3*	10.11	13.46	-10.22	0.0127
TC1900011236.hg.1	Kallikrein related peptidase 13	*KLK13*	4.12	7.49	-10.33	0.0036
TC1200009892.hg.1	Oxidized low density lipoprotein (lectin-like) receptor 1	*OLR1*	11.3	14.71	-10.63	0.0006
TC1900011228.hg.1	Kallikrein related peptidase 5	*KLK5*	9.26	12.68	-10.73	0.0011
TC0700013397.hg.1	Cyclin-dependent kinase 14	*CDK14*	5.21	8.7	-11.28	0.0003
TC0100012734.hg.1	Tumor necrosis factor receptor superfamily, member 9	*TNFRSF9*	5.83	9.35	-11.49	0.0017
TC1200006786.hg.1	Transmembrane protein 52B	*TMEM52B*	7.53	11.09	-11.74	0.0006
TC0200006627.hg.1	Inhibitor of DNA binding 2, dominant negative helix-loop-helix protein	*ID2*	5.88	9.49	-12.23	0.0038
TC0500013384.hg.1	Protein phosphatase 2, regulatory subunit B, beta	*PPP2R2B*	5.87	9.49	-12.32	0.0004
TC0400007840.hg.1	Chemokine (C-X-C motif) ligand 1 (melanoma growth stimulating activity, alpha)	*CXCL1*	8.47	12.1	-12.37	0.0073
TC2100008545.hg.1	Nuclear receptor interacting protein 1	*NRIP1*	7.06	10.77	-13.06	0.0008
TC1900011229.hg.1	Kallikrein related peptidase 6	*KLK6*	9.86	13.66	-13.94	0.0031
TC0200011219.hg.1	Atypical chemokine receptor 3	*ACKR3*	5.04	8.9	-14.48	0.0015
TC0400012644.hg.1	Sorbin and SH3 domain containing 2	*SORBS2*	6.74	10.67	-15.23	0.0011
TC1200011460.hg.1	KIT ligand	*KITLG*	4.39	8.48	-17.11	0.0024
TC2000009522.hg.1	Cytochrome P450, family 24, subfamily A, polypeptide 1	*CYP24A1*	8.84	13.83	-31.78	0.0036

TC2000007083.hg.1	Inhibitor of DNA binding 1, dominant negative helix-loop-helix protein	*ID1*	7.4	12.59	-36.73	0.0008
TC1900011233.hg.1	Kallikrein related peptidase 10	*KLK10*	5.86	11.13	-38.53	0.0007
TC1700007557.hg.1	Chemokine (C-C motif) ligand 2	*CCL2*	5.49	12.74	-151.6	0.0006

Supplementary Table S2. The numbers and fractions of reads with a quality score >5 or >10, as assessed via NanoStat.

	**SAS (PCR amplicon)**	**SAS tumour spheres (PCR amplicon)**	**H103 (PCR amplicon)**	**SAS (Native)**	**SAS tumour spheres (Native)**	**H103 (Native)**
Q5	18,909 (74.2)	86,974 (78.9)	1,866 (52.9)	3,819 (52.3)	10,335 (63.4)	2,774 (55.8)
Q10	2,275 (8.9)	34,003 (30.8)	6 (17.0)	1,951 (26.7)	1,110 (6.8)	516 (10.4)

Supplementary Table S3. Assessment of variant-calling accuracy using PCR-amplified SAS tumour spheres sequencing data.

**Mapping quality score**	10	20	30	40	50	60
Raw variants	61	61	62	63	63	60
Low allele depth (<20.0)	3	3	3	3	3	0
Low quality by depth score (<2.0)	11	11	12	13	13	13
False-positive rate (%) [a]	2/47 (4.26)	2/47 (4.26)	2/47 (4.26)	3/47 (4.26)	2/47 (4.26)	3/47 (6.38)
True-positive rate (%) [b]	45/47 (95.74)	45/47 (95.74)	45/47 (95.74)	45/47 (95.74)	45/47 (95.74)	44/47 (93.62)

[a] The false-positive rate was defined as the fraction of the pass-filter variants that were incorrectly called.
[b] The true-positive rate was defined as the fraction of the pass-filter variants that were correctly called.

Supplementary Table S4. Mitochondrial gene expression profiles of SAS tumour spheres compared to SAS via microarray analysis. RMA method was used to generate the expression levels, where the intensities were background corrected, quantile normalized and log2 transformed. FDR is defined as the proportion of false positive among the declared DEGs.

Probe ID	Encoded protein	Gene symbol	Intensity average (log2)		Fold change	FDR p-value
			SAS tumour spheres	SAS		
TC0M00006454.hg.1	NADH dehydrogenase, subunit 6 (complex I)	*MT-ND6*	12.43	12.06	1.29	0.5036
TC0M00006440.hg.1	NADH dehydrogenase, subunit 3 (complex I)	*MT-ND3*	15.41	15.32	1.06	0.2296
TC0M00006442.hg.1	NADH dehydrogenase, subunit 5 (complex I)	*MT-ND5*	17.73	17.75	-1.01	0.4256
TC0M00006441.hg.1	NADH dehydrogenase, subunit 4L (complex I); NADH dehydrogenase, subunit 4 (complex I)	*MT-ND4L; MT-ND4*	17.88	17.91	-1.02	0.321
TC0M00006434.hg.1	NADH dehydrogenase, subunit 2 (complex I)	*MT-ND2*	13.04	13.08	-1.03	0.9692
TC0M00006439.hg.1	ATP synthase F_o subunit 8; ATP synthase F_o subunit 6; cytochrome c oxidase III	*MT-ATP8; MT-ATP6; MT-CO3*	17.73	17.79	-1.04	0.1047
TC0M00006432.hg.1	NADH dehydrogenase, subunit 1 (complex I)	*MT-ND1*	17.68	17.74	-1.04	0.1762
TC0M00006435.hg.1	cytochrome c oxidase subunit I	*MT-CO1*	17.61	17.67	-1.04	0.3327
TC0M00006443.hg.1	cytochrome b	*MT-CYB*	17.26	17.38	-1.09	0.0739
TC0M00006437.hg.1	cytochrome c oxidase subunit II	*MT-CO2*	17.21	17.44	-1.18	0.0323

Supplementary Table S5. Mitochondrial gene expression profiles of H103 compared to SAS via microarray analysis. RMA method was used to generate the expression levels, where the intensities were background corrected, quantile normalized and log2 transformed. FDR is defined as the proportion of false positive among the declared DEGs.

**Probe ID**	**Encoded protein**	**Gene symbol**	**Intensity average (log2)**		**Fold change**	**FDR p-value**
			**H103**	**SAS**		
TC0M00006435.hg.1	cytochrome c oxidase subunit I	*MT-CO1*	17.82	17.67	1.11	0.1445
TC0M00006454.hg.1	NADH dehydrogenase, subunit 6 (complex I)	*MT-ND6*	12.18	12.06	1.09	0.8689
TC0M00006443.hg.1	cytochrome b	*MT-CYB*	17.5	17.38	1.08	0.2306
TC0M00006439.hg.1	ATP synthase F_o subunit 8; ATP synthase F_o subunit 6; cytochrome c oxidase III	*MT-ATP8; MT-ATP6; MT-CO3*	17.87	17.79	1.06	0.3642
TC0M00006432.hg.1	NADH dehydrogenase, subunit 1 (complex I)	*MT-ND1*	17.82	17.74	1.06	0.4244
TC0M00006441.hg.1	NADH dehydrogenase, subunit 4L (complex I); NADH dehydrogenase, subunit 4 (complex I)	*MT-ND4L; MT-ND4*	17.97	17.91	1.04	0.282
TC0M00006442.hg.1	NADH dehydrogenase, subunit 5 (complex I)	*MT-ND5*	17.75	17.75	1.01	0.8643
TC0M00006437.hg.1	cytochrome c oxidase subunit II	*MT-CO2*	17.44	17.44	-1	0.7099
TC0M00006440.hg.1	NADH dehydrogenase, subunit 3 (complex I)	*MT-ND3*	15.2	15.32	-1.08	0.2592
TC0M00006434.hg.1	NADH dehydrogenase, subunit 2 (complex I)	*MT-ND2*	12.39	13.08	-1.62	0.0163

Supplementary Table S6. List of qPCR primer sequences for microarray validation.

Gene	Description	Product length (bp)	Efficiency (%), R^2	Primer sequences (5' to 3')	
MGST1	Microsomal glutathione S-transferase 1	189	113, 0.998	Forward	AGTATTCATGGCTTTTGCATCCT
				Reverse	CTGCGTACACGTTCTACTCTGT
DKK1	Dickkopf WNT signaling pathway inhibitor 1	118	98, 0.999	Forward	GCACCTTGGATGGGTATTCCA
				Reverse	GCACAACACAATCCTGAGGC
SPTSSB	Serine palmitoyltransferase small subunit B	141	114, 0.996	Forward	ACCTAAGCCGCAGGGAGATA
				Reverse	GCAGTAAGTTTGTCCTAAGAAAGT
CCL2	C-C motif chemokine ligand 2	155	109, 0.995	Forward	GATCTCAGTGCAGAGGCTCG
				Reverse	TTTGCTTGTCCAGGTGGTCC
OCT4	POU class 5 homeobox 1	86	98, 0.989	Forward	GTGGAGAGCAACTCCGATG
				Reverse	TGCTCCAGCTTCTCCTTCTC
SOX2	SRY-box transcription factor 2	178	93, 0.987	Forward	CCTCCGGGACATGATCAG
				Reverse	TTCTCCCCCCTCCAGTTC
GAPDH	Glyceraldehyde-3-phosphate dehydrogenase	180	94, 0.997	Forward	CATCATCCCTGCCTCTACTG
				Reverse	GCCTGCTTCACCACCTTC
ACTB	β-actin	104	91, 0.998	Forward	CTTCGCGGGCGACGAT
				Reverse	CCACATAGGAATCCTTCTGACC

Supplementary Table S7. List of mtDNA gene-specific qPCR primer sequences.

Genes	Product length (bp)	Efficiency (%), R^2	Primer sequence (5' to 3')	
MtDNA tRNA$^{Leu(UUR)}$	107	100, 0.982	Forward	CACCCAAGAACAGGGTTTGT
			Reverse	TGGCCATGGGTATGTTGTTA
Nuclear DNA β2-microglobulin	86	99, 0.987	Forward	TGCTGTCTCCATGTTTGATGTATCT
			Reverse	TCTCTGCTCCCCACCTCTAAGT
MtDNA 16S rRNA	97	105, 0.991	Forward	GCCTTCCCCCGTAAATGATA
			Reverse	TTATGCGATTACCGGGCTCT

Supplementary Table S8. List of mtDNA PCR primer sequences (MinION sequencing).

Primer ID	Product length (bp)	Primer sequence (5’ to 3’)	
Mit1	8982	Forward	CGATAGCATTGCGAGACGCTG
		Reverse	GGGTGGCGCTTCCAATTAGGTG
Mit2	9105	Forward	AACCCTCCATAAACCTGGAGTGAC
		Reverse	GTGTTATGGGCCCGGAGCGA
Mit1 with universal sequence*	8982	Forward	TTTCTGTTGGTGCTGATATTGCCGATAGCATTGCGAGACGCTG
		Reverse	ACTTGCCTGTCGCTCTATCTTCGGGTGGCGCTTCCAATTAGGTG
Mit2 with universal sequence*	9105	Forward	TTTCTGTTGGTGCTGATATTGCAACCCTCCATAAACCTGGAGTGAC
		Reverse	ACTTGCCTGTCGCTCTATCTTCGTGTTATGGGCCCGGAGCGA

*Primers include universal tailing sequences (underlined) for barcoding PCR.

Supplementary Table S9. PCR primers used to generate overlapping amplicons for Sanger sequencing of the mitochondrial genome.

Primer ID	Product length (bp)	Primer sequence (5' to 3')	
Mt1	870	Forward	CTAACCCCATACCCCGAACC
		Reverse	TAAGGGCTATCGTAGTTTTCTGGG
Mt2	952	Forward	CGTTAGGTCAAGGTGTAGCCC
		Reverse	TGGTCCAATTGGGTGTGAGG
Mt3	883	Forward	CAAGCTCAACACCCACTACCTAA
		Reverse	GGATTACTCCGGTCTGAACTCA
Mt4	834	Forward	CAGCCGCTATTAAAGGTTCGTT
		Reverse	CCAAGGGTCATGATGGCAGG
Mt5	888	Forward	CCACCCTTATCACAACACAAGA
		Reverse	AGAAGGATTATGGATGCGGTTG
Mt6	858	Forward	GCCATCAAGTATTTCCTCACGC
		Reverse	AGGGGAGATAGGTAGGAGTAGC
Mt7	987	Forward	CATCCCCACCATCATAGCCAC
		Reverse	ACTGTTCAACCTGTTCCTGCT
Mt8	1028	Forward	CCTCTCTCCTACTCCTGCTC
		Reverse	TGTGGTGTATGCATCGGGG
Mt9	767	Forward	ACCTACGCCAAAATCCATTTCA
		Reverse	GTGGCCAATTGATTTGATGGTAAG
Mt10	952	Forward	CCGCCATCATCCTAGTCCTC
		Reverse	TAGTATAAGAGATCAGGTTCGTCCT
Mt11	1017	Forward	CTCATCAACAACCGACTAATCACC
		Reverse	GGTGAGCTCAGGTGATTGATACT
Mt12	777	Forward	CACATCCGTATTACTCGCATCAG
		Reverse	TGTAGTCACTCATAGGCCAGAC
Mt13	827	Forward	CCGCGTCCCTTTCTCCATAAA
		Reverse	AGTGGTTCACTGGATAAGTGGC
Mt14	956	Forward	TTCCCCAACCTTTTCCTCCG
		Reverse	GGGGTAAGGCGAGGTTAGC
Mt15	872	Forward	CTCAAACTACGAACGCACTCAC
		Reverse	ACGAACAATGCTACAGGGATGA
Mt16	860	Forward	TCGAACTGACACTGAGCCAC
		Reverse	AGGTTGTGGATGATGGACCC
Mt17	1055	Forward	CACGCCTTCTTCAAAGCCATAC
		Reverse	CGATGGAGGTAGGATTGGTGC
Mt18a	1079	Forward	CCGAATCAACCCTGACCCC
		Reverse	AGAATAGGAGGTGGAGTGTTGC
Mt18b	883	Forward	GCACCAAATCTCCACCTCCA

		Reverse	GCGTCTGGTGAGTAGTGCAT
Mt18c	775	Forward	CACCAATGACCCCAATACGC
		Reverse	GTATAATTGTCTGGGTCGCCT
Mt19a	976	Forward	CAGTAGACAGTCCCACCCTC
		Reverse	TTGAGGGTTGATTGCTGTACTTG
Mt19b	483	Forward	CTCACCCATCAACAACCGCT
		Reverse	GGGGAACGTGTGGGCTATTT
Mt20a	955	Forward	CCCCTCCCCATGCTTACAAG
		Reverse	GGGTGTCTTTGGGGTTTGGT
Mt20b	389	Forward	CAGCACTTAAACACATCTCTGCC
		Reverse	CTGGAACGGGGATGCTTGC

Supplementary Table S10. List of nested-PCR primers and the 595-bp DNA fragment sequences.

ID		Sequence (5' to 3')
1st PCR primer	Forward	AACCACAGTTTCATGCCCATC
	Reverse	GCTTCCCCACCCTTACTAACA
2nd PCR primer	Forward	ACCCTATAGCACCCCCTCTAC
	Reverse	CATCGCTACCTCCCTGACAAG
595-bp DNA fragment		CTACACGACCGGGGGTATACTACGGTCAATGCTCTGAAATCTG TGGAGCAAACCACAGTTTCATGCCCATCGTCCTAGAATTAATT CCCCTAAAAATCTTTGAAATAGGGCCCGTATTTACCCTATAGC ACCCCCTCTACCCCCTCTAGAGCCCACTGTAAAGCTAACTTAG CATTAACCTTTTAAGTTAAAGATTAAGAGAACCAACACCTCTT TACAGTGAAATGCCCCAACTAAATACTACCGTATGGCCCACCA TAATTACCCCCATACTCCTTACACTATTCCTCATCACCCAACTA AAAATATTAAACACAAACTACCACCTACCTCCCTCACCATTGG CAGCCTAGCATTAGCAGGAATACCTTTCCTCACAGGTTTCTAC TCCAAAGACCACATCATCGAAACCGCAAACATATCATACACA AACGCCTGAGCCCTATCTATTACTCTCATCGCTACCTCCCTGAC AAGCGCCTATAGCACTCGAATAATTCTTCTCACCCTAACAGGT CAACCTCGCTTCCCCACCCTTACTAACATTAACGAAAATAACC CCACCCTACTAAACCCCATTAAACGCCTGGCAGCC
