# Supplementary material for: Mitochondrial DNA alterations may influence the cisplatin responsiveness of oral squamous cell carcinoma
Source: Sci Rep. 2020 May 12;10:7885. doi: 10.1038/s41598-020-64664-3 (PMC7217862; doi:10.1038/s41598-020-64664-3)
Supplement: Supplementary file 9 — Dataset S8. [file 41598_2020_64664_MOESM9_ESM.zip › Supplementary Dataset S8/MULTI-COLOR FLOW CYTOMETRY CD338 & CD117 SURFACE MARKERS ANALYSIS/PARENTAL SAS/EXP1 PARENTAL SAS CD338 CD117.pdf]

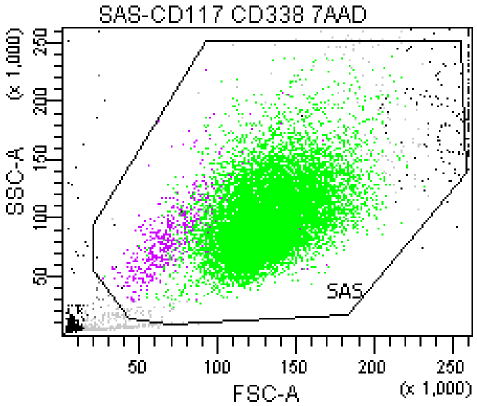

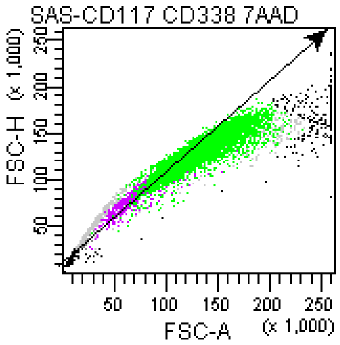

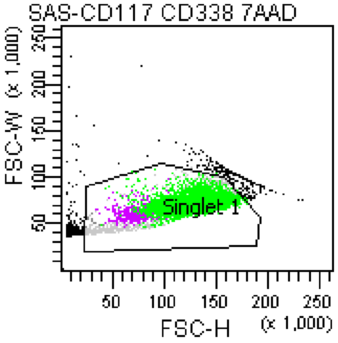

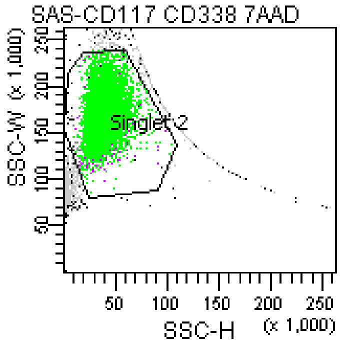

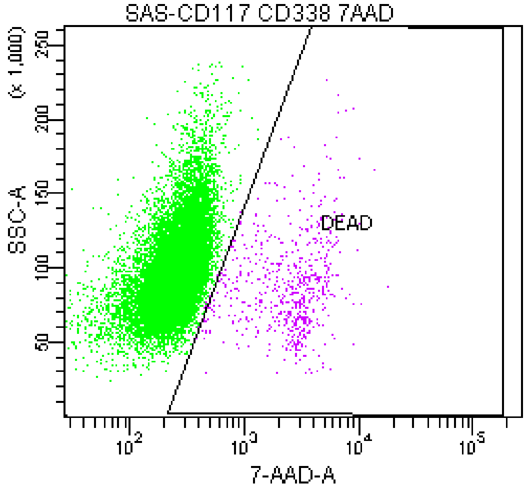

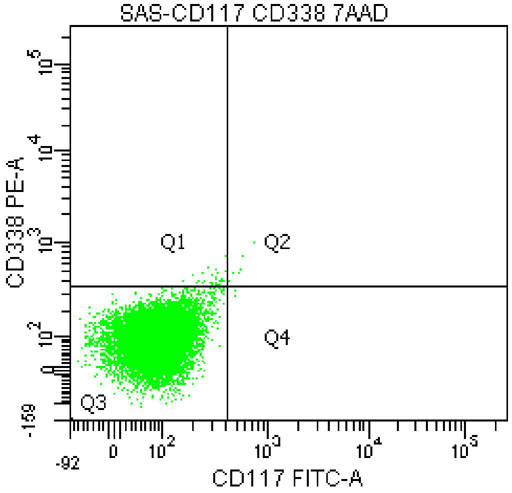

Tube: CD117 CD338 7AAD

Population	#Events	%Parent
All Events	16,312	####
Singlet 1	15,575	95.5
Singlet 2	15,015	96.4
SAS	15,000	99.9
DEAD	655	4.4
LIVE	14,345	95.6
Q1	23	0.2
Q2	12	0.1
Q3	14,307	99.7
Q4	3	0.0

Experiment Name: 11082016 SAS 3C
Specimen Name: SAS
Tube Name: CD117 CD338 7AAD
Record Date: Aug 11, 2016 11:19:38 AM
$OP: ToxicologyLab

Population	#Events	%Parent	CD117 F... Mean	CD338 P... Mean
All Events	16,312	####	98	96
Singlet 1	15,575	95.5	96	94
Singlet 2	15,015	96.4	95	93
SAS	15,000	99.9	95	93
DEAD	655	4.4	158	156
LIVE	14,345	95.6	92	90
Q1	23	0.2	301	427
Q2	12	0.1	481	529
Q3	14,307	99.7	91	89
Q4	3	0.0	461	266
