# Supplementary material for: Mitochondrial DNA alterations may influence the cisplatin responsiveness of oral squamous cell carcinoma
Source: Sci Rep. 2020 May 12;10:7885. doi: 10.1038/s41598-020-64664-3 (PMC7217862; doi:10.1038/s41598-020-64664-3)
Supplement: Supplementary file 9 — Dataset S8. [file 41598_2020_64664_MOESM9_ESM.zip › Supplementary Dataset S8/MULTI-COLOR FLOW CYTOMETRY CD338 & CD117 SURFACE MARKERS ANALYSIS/PARENTAL SAS/EXP1 PARENTAL SAS CONTROL.pdf]

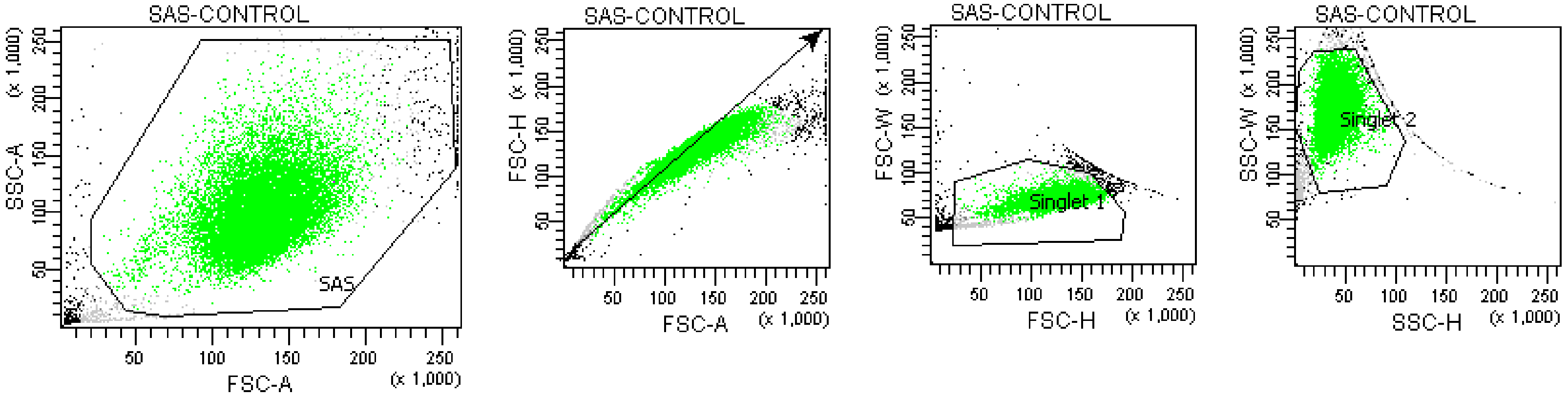

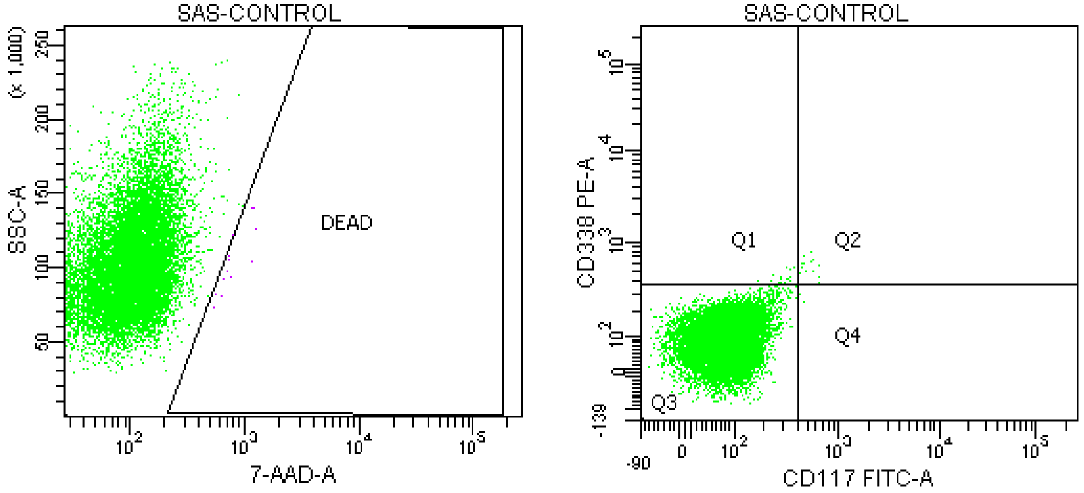

Tube: CONTROL

Population	#Events	%Parent
All Events	16,692	####
Singlet 1	15,998	95.8
Singlet 2	15,496	96.9
SAS	15,478	99.9
DEAD	14	0.1
LIVE	15,464	99.9
Q1	17	0.1
Q2	14	0.1
Q3	15,424	99.7
Q4	9	0.1

Experiment Name: 11082016 SAS 3C
Specimen Name: SAS
Tube Name: CONTROL
Record Date: Aug 11, 2016 11:16:30 AM
$OP: ToxicologyLab

Population	#Events	%Parent	CD117 F... Mean	CD338 P... Mean
All Events	16,692	####	92	92
Singlet 1	15,998	95.8	89	89
Singlet 2	15,496	96.9	87	86
SAS	15,478	99.9	86	86
DEAD	14	0.1	454	541
LIVE	15,464	99.9	86	86
Q1	17	0.1	309	423
Q2	14	0.1	510	547
Q3	15,424	99.7	85	85
Q4	9	0.1	464	304
