# Supplementary material for: Mitochondrial DNA alterations may influence the cisplatin responsiveness of oral squamous cell carcinoma
Source: Sci Rep. 2020 May 12;10:7885. doi: 10.1038/s41598-020-64664-3 (PMC7217862; doi:10.1038/s41598-020-64664-3)
Supplement: Supplementary file 9 — Dataset S8. [file 41598_2020_64664_MOESM9_ESM.zip › Supplementary Dataset S8/MULTI-COLOR FLOW CYTOMETRY CD338 & CD117 SURFACE MARKERS ANALYSIS/PARENTAL SAS/EXP2 PARENTAL SAS CD338 CD117.pdf]

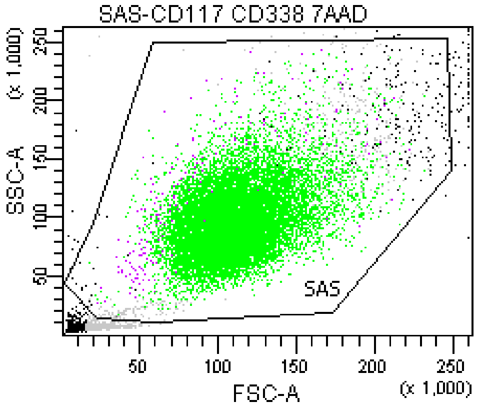

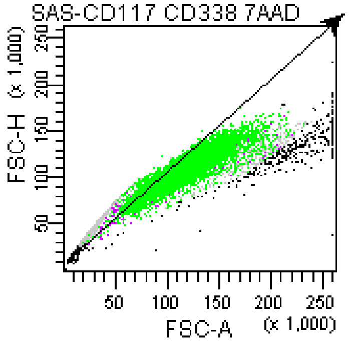

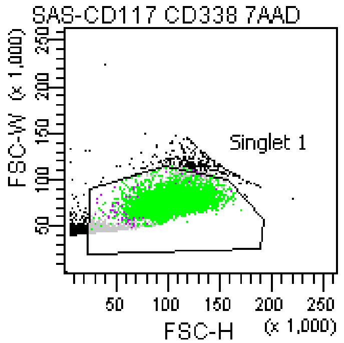

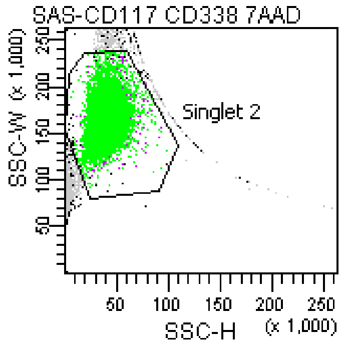

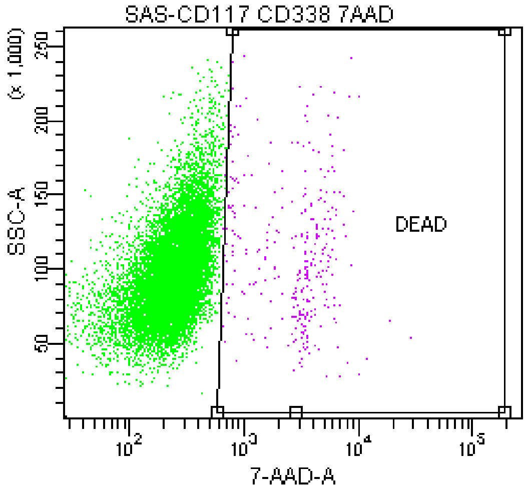

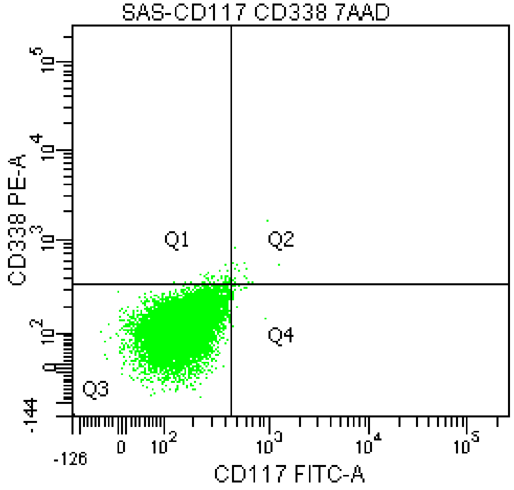

Tube: CD117 CD338 7AAD

Population	#Events	%Parent
All Events	17,840	####
Singlet 1	16,062	90.0
Singlet 2	15,001	93.4
SAS	15,000	100.0
DEAD	323	2.2
LIVE	14,677	97.8
Q1	26	0.2
Q2	13	0.1
Q3	14,601	99.5
Q4	37	0.3

Experiment Name: 09082017 SAS 3C
Specimen Name: SAS
Tube Name: CD117 CD338 7AAD
Record Date: Aug 9, 2017 11:42:34 AM
$OP: ToxicologyLab

Population	#Events	%Parent	CD117 FIT... Mean	CD338 PE-A Mean
All Events	17,840	####	169	118
Singlet 1	16,062	90.0	175	121
Singlet 2	15,001	93.4	178	120
SAS	15,000	100.0	178	120
DEAD	323	2.2	414	290
LIVE	14,677	97.8	173	116
Q1	26	0.2	340	394
Q2	13	0.1	619	563
Q3	14,601	99.5	171	115
Q4	37	0.3	496	267
