# Supplementary material for: Mitochondrial DNA alterations may influence the cisplatin responsiveness of oral squamous cell carcinoma
Source: Sci Rep. 2020 May 12;10:7885. doi: 10.1038/s41598-020-64664-3 (PMC7217862; doi:10.1038/s41598-020-64664-3)
Supplement: Supplementary file 9 — Dataset S8. [file 41598_2020_64664_MOESM9_ESM.zip › Supplementary Dataset S8/MULTI-COLOR FLOW CYTOMETRY CD338 & CD117 SURFACE MARKERS ANALYSIS/PARENTAL SAS/EXP2 PARENTAL SAS CONTROL.pdf]

FACSDiva Version 6.1.3

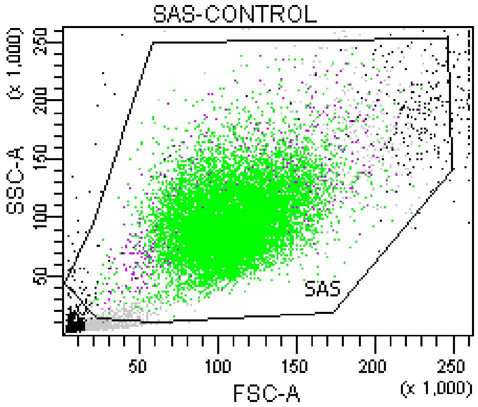

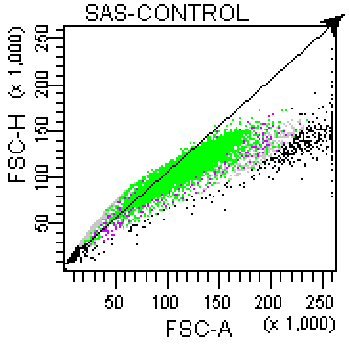

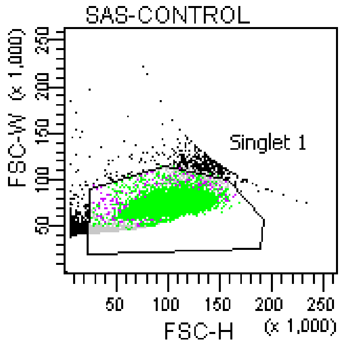

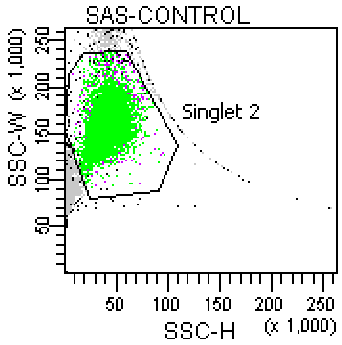

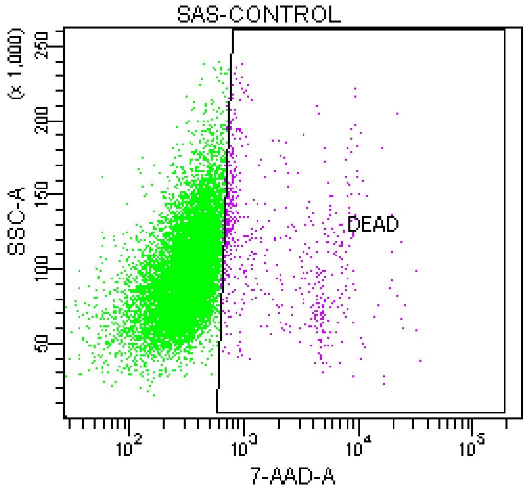

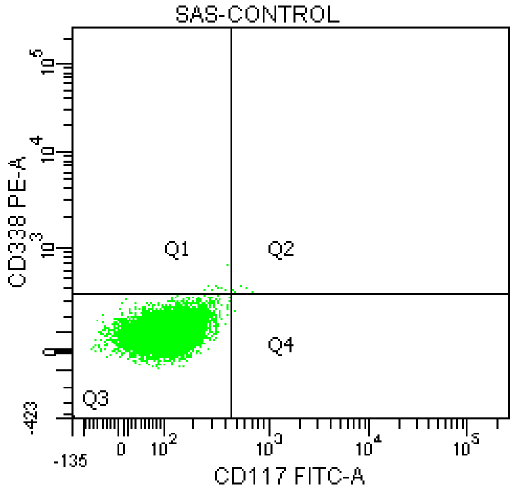

Tube: CONTROL

Population	#Events	%Parent
All Events	19,150	####
Singlet 1	16,453	85.9
Singlet 2	15,002	91.2
SAS	15,000	100.0
DEAD	715	4.8
LIVE	14,285	95.2
Q1	9	0.1
Q2	3	0.0
Q3	14,267	99.9
Q4	6	0.0

Experiment Name:	09082017 SAS 3C
Specimen Name:	SAS
Tube Name:	CONTROL
Record Date:	Aug 9, 2017 11:40:00 AM
$OP:	ToxicologyLab

			CD117 FIT...	CD338 PE-A
Population	#Events	%Parent	Mean	Mean
All Events	19,150	####	103	90
Singlet 1	16,453	85.9	111	95
Singlet 2	15,002	91.2	115	98
SAS	15,000	100.0	115	98
DEAD	715	4.8	194	177
LIVE	14,285	95.2	111	94
Q1	9	0.1	340	416
Q2	3	0.0	544	388
Q3	14,267	99.9	110	93
Q4	6	0.0	505	274
