# Supplementary material for: Mitochondrial DNA alterations may influence the cisplatin responsiveness of oral squamous cell carcinoma
Source: Sci Rep. 2020 May 12;10:7885. doi: 10.1038/s41598-020-64664-3 (PMC7217862; doi:10.1038/s41598-020-64664-3)
Supplement: Supplementary file 9 — Dataset S8. [file 41598_2020_64664_MOESM9_ESM.zip › Supplementary Dataset S8/MULTI-COLOR FLOW CYTOMETRY CD338 & CD117 SURFACE MARKERS ANALYSIS/PARENTAL SAS/EXP3 PARENTAL SAS CD338 CD117.pdf]

FACSDiva Version 6.1.3

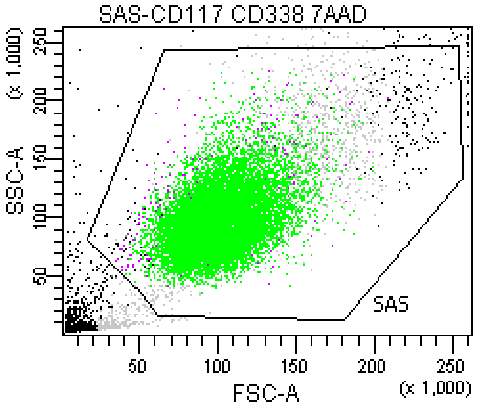

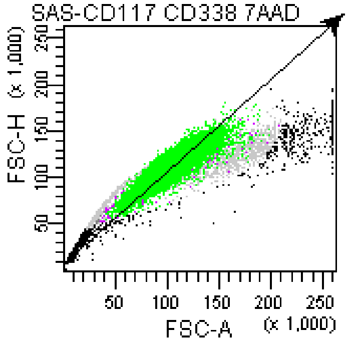

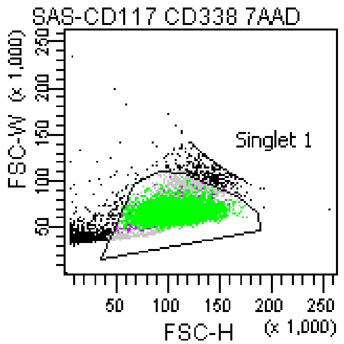

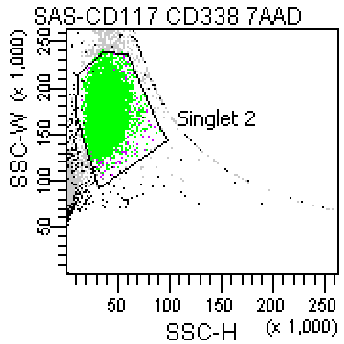

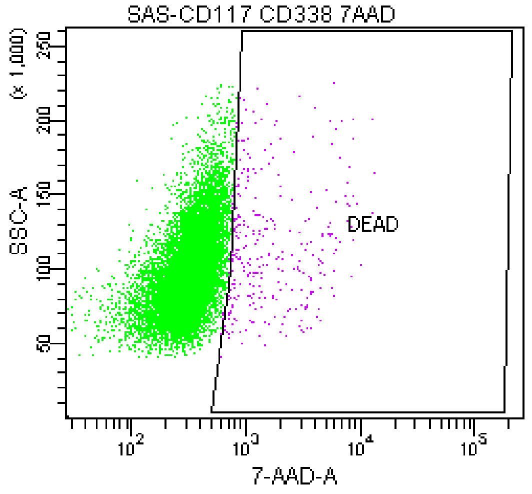

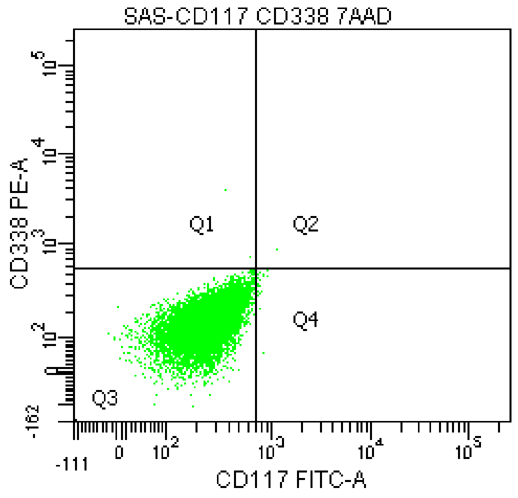

Tube: CD117 CD338 7AAD

Population	#Events	%Parent
All Events	18,168	####
Singlet 1	16,296	89.7
Singlet 2	15,037	92.3
SAS	15,029	99.9
DEAD	341	2.3
LIVE	14,688	97.7
Q1	2	0.0
Q2	1	0.0
Q3	14,642	99.7
Q4	43	0.3

Experiment Name: 11082017 SAS 3C
Specimen Name: SAS
Tube Name: CD117 CD338 7AAD
Record Date: Aug 11, 2017 11:36:09 AM
$OP: ToxicologyLab

Population	#Events	%Parent	CD117 FITC-A Mean	CD338 PE-A Mean
All Events	18,168	####	294	170
Singlet 1	16,296	89.7	298	171
Singlet 2	15,037	92.3	285	164
SAS	15,029	99.9	285	164
DEAD	341	2.3	514	375
LIVE	14,688	97.7	280	159
Q1	2	0.0	497	2,231
Q2	1	0.0	1,151	828
Q3	14,642	99.7	278	158
Q4	43	0.3	749	366
