# Supplementary material for: Mitochondrial DNA alterations may influence the cisplatin responsiveness of oral squamous cell carcinoma
Source: Sci Rep. 2020 May 12;10:7885. doi: 10.1038/s41598-020-64664-3 (PMC7217862; doi:10.1038/s41598-020-64664-3)
Supplement: Supplementary file 9 — Dataset S8. [file 41598_2020_64664_MOESM9_ESM.zip › Supplementary Dataset S8/MULTI-COLOR FLOW CYTOMETRY CD338 & CD117 SURFACE MARKERS ANALYSIS/PARENTAL SAS/EXP3 PARENTAL SAS CONTROL.pdf]

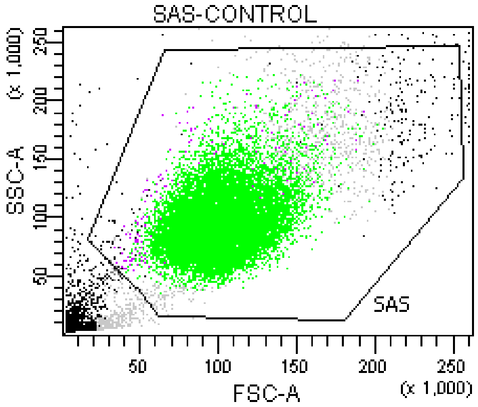

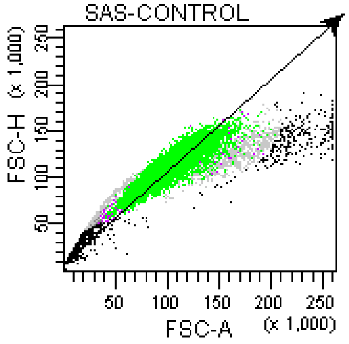

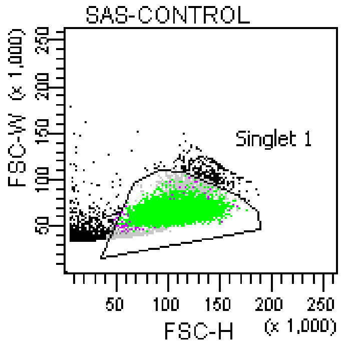

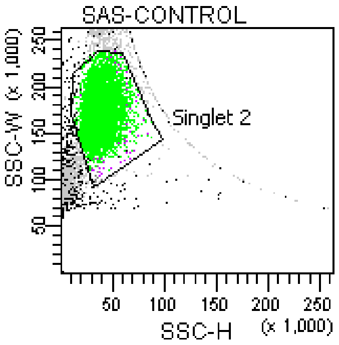

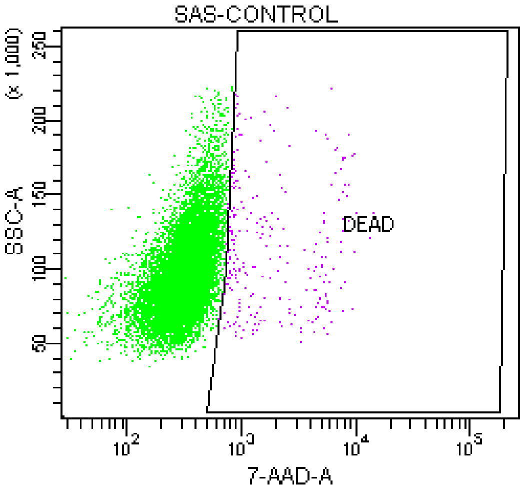

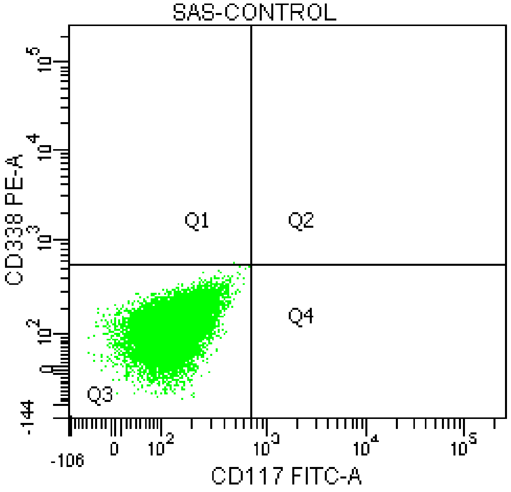

Tube: CONTROL

Population	#Events	%Parent
All Events	18,198	####
Singlet 1	16,268	89.4
Singlet 2	15,080	92.7
SAS	15,063	99.9
DEAD	280	1.9
LIVE	14,783	98.1
Q1	1	0.0
Q2	0	0.0
Q3	14,781	100.0
Q4	1	0.0

Experiment Name: 11082017 SAS 3C
Specimen Name: SAS
Tube Name: CONTROL
Record Date: Aug 11, 2017 11:31:40 AM
$OP: ToxicologyLab

Population	#Events	%Parent	CD117 FITC-A Mean	CD338 PE-A Mean
All Events	18,198	####	148	123
Singlet 1	16,268	89.4	153	127
Singlet 2	15,080	92.7	147	122
SAS	15,063	99.9	147	122
DEAD	280	1.9	326	322
LIVE	14,783	98.1	144	118
Q1	1	0.0	504	561
Q2	0	0.0	####	####
Q3	14,781	100.0	144	118
Q4	1	0.0	704	503
