# Supplementary material for: Mitochondrial DNA alterations may influence the cisplatin responsiveness of oral squamous cell carcinoma
Source: Sci Rep. 2020 May 12;10:7885. doi: 10.1038/s41598-020-64664-3 (PMC7217862; doi:10.1038/s41598-020-64664-3)
Supplement: Supplementary file 9 — Dataset S8. [file 41598_2020_64664_MOESM9_ESM.zip › Supplementary Dataset S8/MULTI-COLOR FLOW CYTOMETRY CD338 & CD117 SURFACE MARKERS ANALYSIS/TUMOR SPHERE/EXP1 TUMOR SPHERE CD338 CD117.pdf]

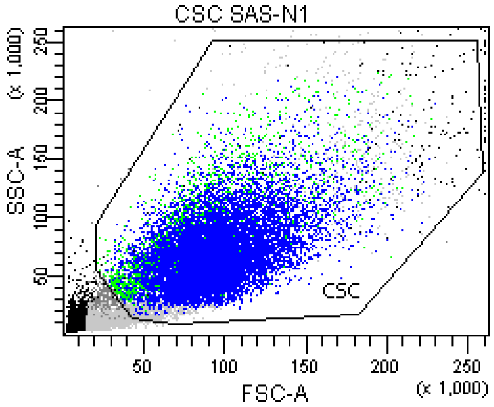

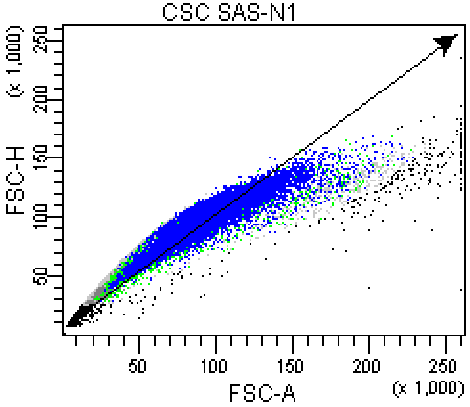

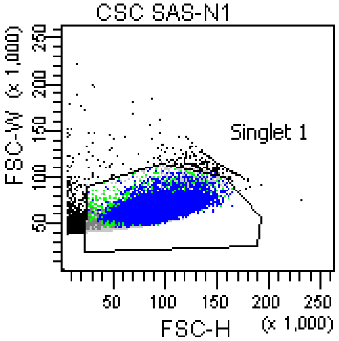

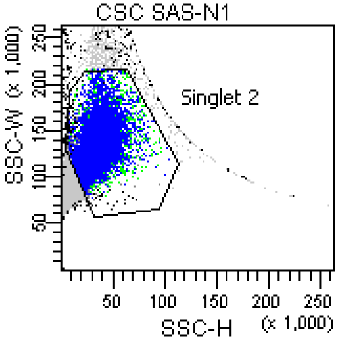

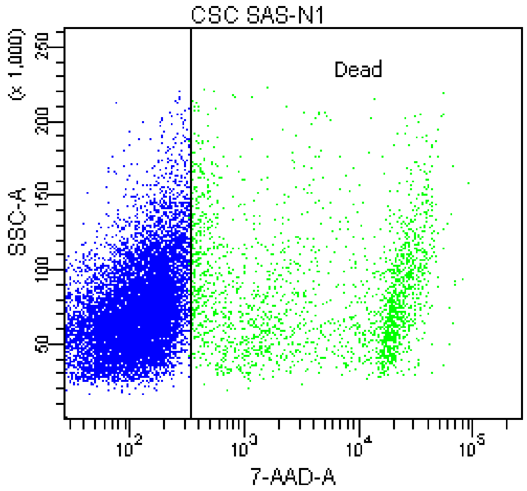

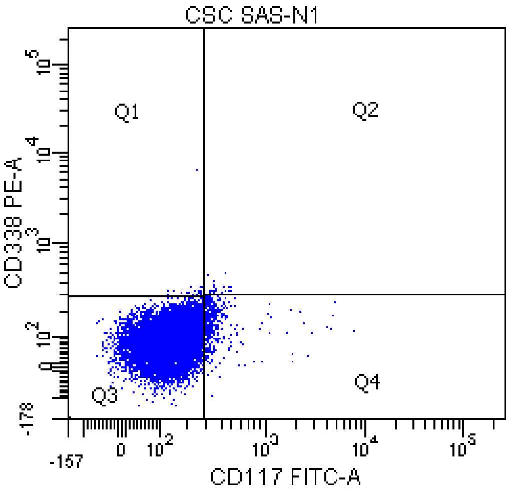

Experiment Name: 28122016 3C CSC Characterization
Specimen Name: CSC SAS
Tube Name: N1
Record Date: Dec 28, 2016 3:34:22 PM
$OP: ToxicologyLab

Population	#Events	%Parent	CD117 FITC-A Mean	CD338 PE-A Mean
All Events	24,396	####	144	349
Singlet 1	19,496	79.9	162	403
Singlet 2	15,154	77.7	182	459
CSC	14,904	98.4	183	455
Dead	2,297	15.4	393	2,520
Live	12,607	84.6	145	79
Q1	17	0.1	228	654
Q2	19	0.2	387	340
Q3	12,037	95.5	132	76
Q4	534	4.2	415	137

Tube: N1

Population	#Events	%Parent
All Events	24,396	####
Singlet 1	19,496	79.9
Singlet 2	15,154	77.7
CSC	14,904	98.4
Dead	2,297	15.4
Live	12,607	84.6
Q1	17	0.1
Q2	19	0.2
Q3	12,037	95.5
Q4	534	4.2
