# Supplementary material for: Mitochondrial DNA alterations may influence the cisplatin responsiveness of oral squamous cell carcinoma
Source: Sci Rep. 2020 May 12;10:7885. doi: 10.1038/s41598-020-64664-3 (PMC7217862; doi:10.1038/s41598-020-64664-3)
Supplement: Supplementary file 9 — Dataset S8. [file 41598_2020_64664_MOESM9_ESM.zip › Supplementary Dataset S8/MULTI-COLOR FLOW CYTOMETRY CD338 & CD117 SURFACE MARKERS ANALYSIS/TUMOR SPHERE/EXP2 TUMOR SPHERE CD338 CD117.pdf]

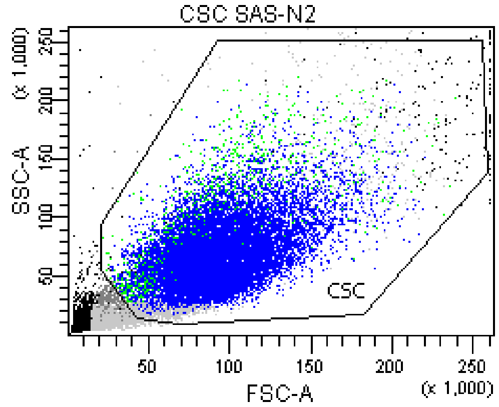

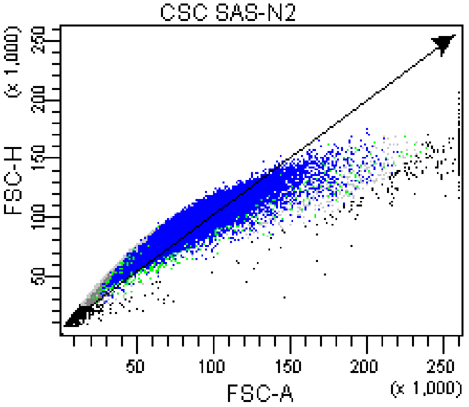

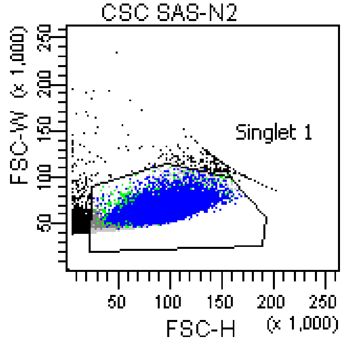

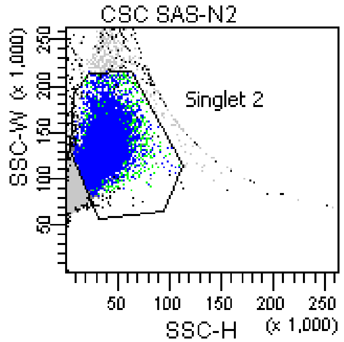

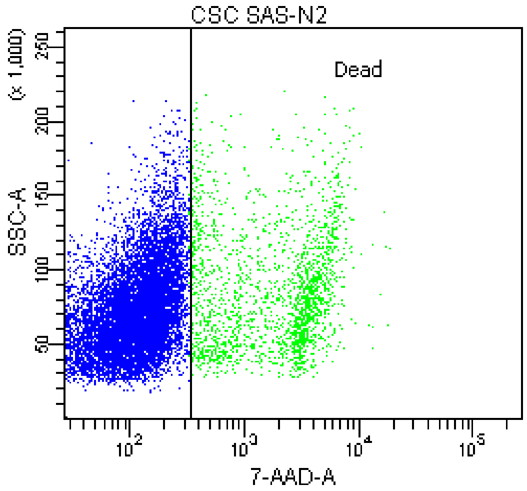

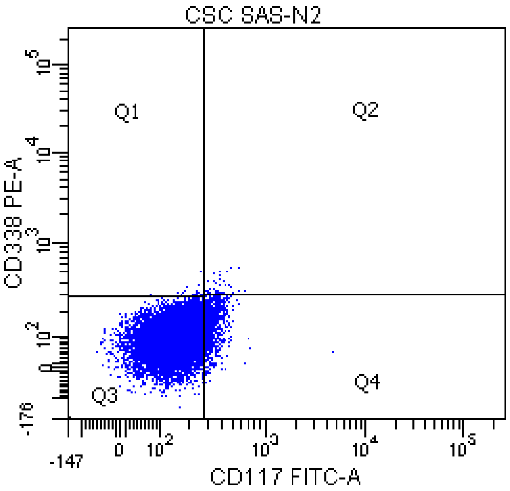

Experiment Name: 28122016 3C CSC Characterization
Specimen Name: CSC SAS
Tube Name: N2
Record Date: Dec 28, 2016 3:37:22 PM
$OP: ToxicologyLab

Population	#Events	%Parent	CD117 FITC-A Mean	CD338 PE-A Mean
All Events	23,683	####	153	139
Singlet 1	18,973	80.1	170	142
Singlet 2	15,145	79.8	190	153
CSC	14,899	98.4	191	151
Dead	2,111	14.2	331	521
Live	12,788	85.8	167	89
Q1	17	0.1	220	316
Q2	30	0.2	408	370
Q3	11,627	90.9	151	84
Q4	1,114	8.7	335	140

Tube: N2

Population	#Events	%Parent
All Events	23,683	####
Singlet 1	18,973	80.1
Singlet 2	15,145	79.8
CSC	14,899	98.4
Dead	2,111	14.2
Live	12,788	85.8
Q1	17	0.1
Q2	30	0.2
Q3	11,627	90.9
Q4	1,114	8.7
