# Supplementary material for: Mitochondrial DNA alterations may influence the cisplatin responsiveness of oral squamous cell carcinoma
Source: Sci Rep. 2020 May 12;10:7885. doi: 10.1038/s41598-020-64664-3 (PMC7217862; doi:10.1038/s41598-020-64664-3)
Supplement: Supplementary file 9 — Dataset S8. [file 41598_2020_64664_MOESM9_ESM.zip › Supplementary Dataset S8/MULTI-COLOR FLOW CYTOMETRY CD338 & CD117 SURFACE MARKERS ANALYSIS/TUMOR SPHERE/EXP3 TUMOR SPHERE CD338 CD117.pdf]

# FACSDiva Version 6.1.3

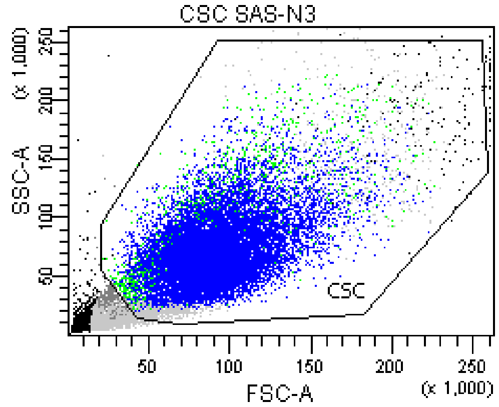


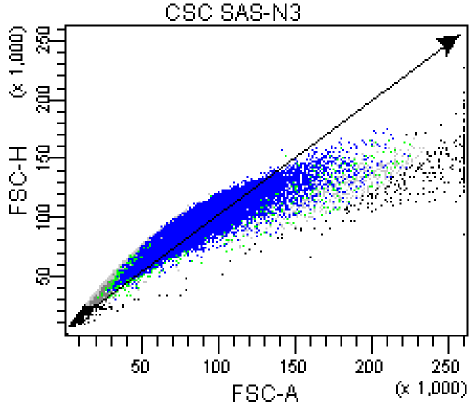


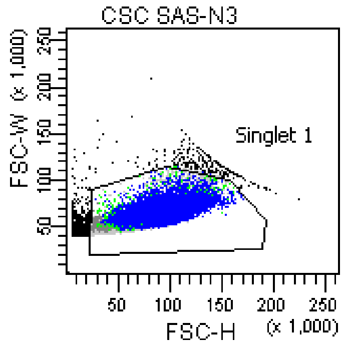


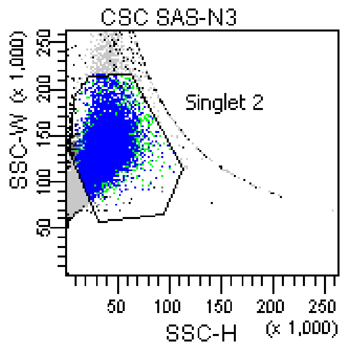


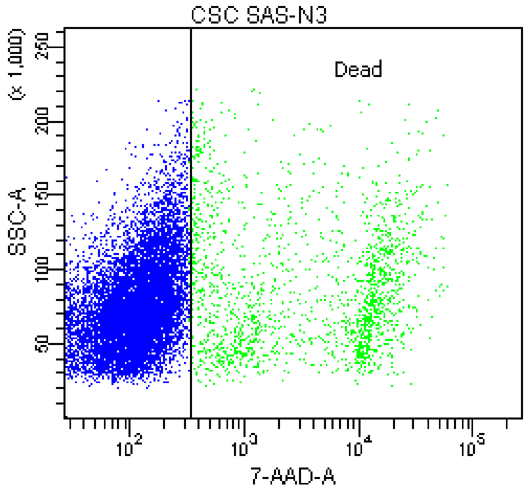


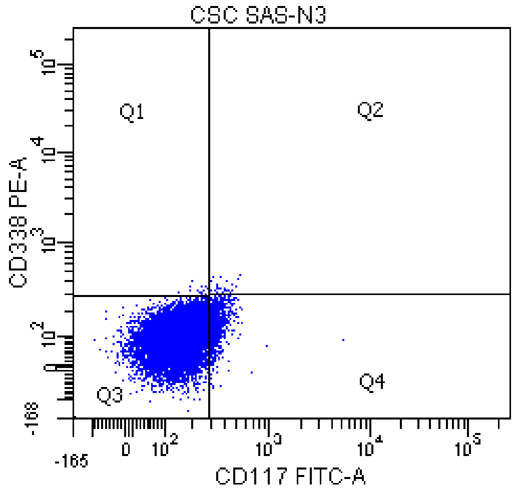


Experiment Name: 28122016 3C CSC Characterization
Specimen Name: CSC SAS
Tube Name: N3
Record Date: Dec 28, 2016 3:31:06 PM
$OP: ToxicologyLab

| Population | #Events | %Parent | CD117 FITC-A Mean | CD338 PE-A Mean |
|---|---|---|---|---|
| All Events | 24,034 | #### | 143 | 245 |
| Singlet 1 | 19,440 | 80.9 | 159 | 265 |
| Singlet 2 | 15,101 | 77.7 | 180 | 298 |
| CSC | 14,894 | 98.6 | 180 | 293 |
| Dead | 1,928 | 12.9 | 301 | 1,702 |
| Live | 12,966 | 87.1 | 162 | 84 |
| Q1 | 14 | 0.1 | 231 | 314 |
| Q2 | 26 | 0.2 | 388 | 342 |
| Q3 | 11,935 | 92.0 | 147 | 78 |
| Q4 | 991 | 7.6 | 337 | 135 |

Tube: N3

| Population | #Events | %Parent |
|---|---|---|
| All Events | 24,034 | #### |
| Singlet 1 | 19,440 | 80.9 |
| Singlet 2 | 15,101 | 77.7 |
| CSC | 14,894 | 98.6 |
| Dead | 1,928 | 12.9 |
| Live | 12,966 | 87.1 |
| Q1 | 14 | 0.1 |
| Q2 | 26 | 0.2 |
| Q3 | 11,935 | 92.0 |
| Q4 | 991 | 7.6 |
