# Supplementary material for: Mitochondrial DNA alterations may influence the cisplatin responsiveness of oral squamous cell carcinoma
Source: Sci Rep. 2020 May 12;10:7885. doi: 10.1038/s41598-020-64664-3 (PMC7217862; doi:10.1038/s41598-020-64664-3)
Supplement: Supplementary file 9 — Dataset S8. [file 41598_2020_64664_MOESM9_ESM.zip › Supplementary Dataset S8/SINGLE COLOR FLOW CYTOMETRY CD44 SURFACE MARKER ANALYSIS/PARENTAL SAS/EXP1 PARENTAL SAS CD44.pdf]

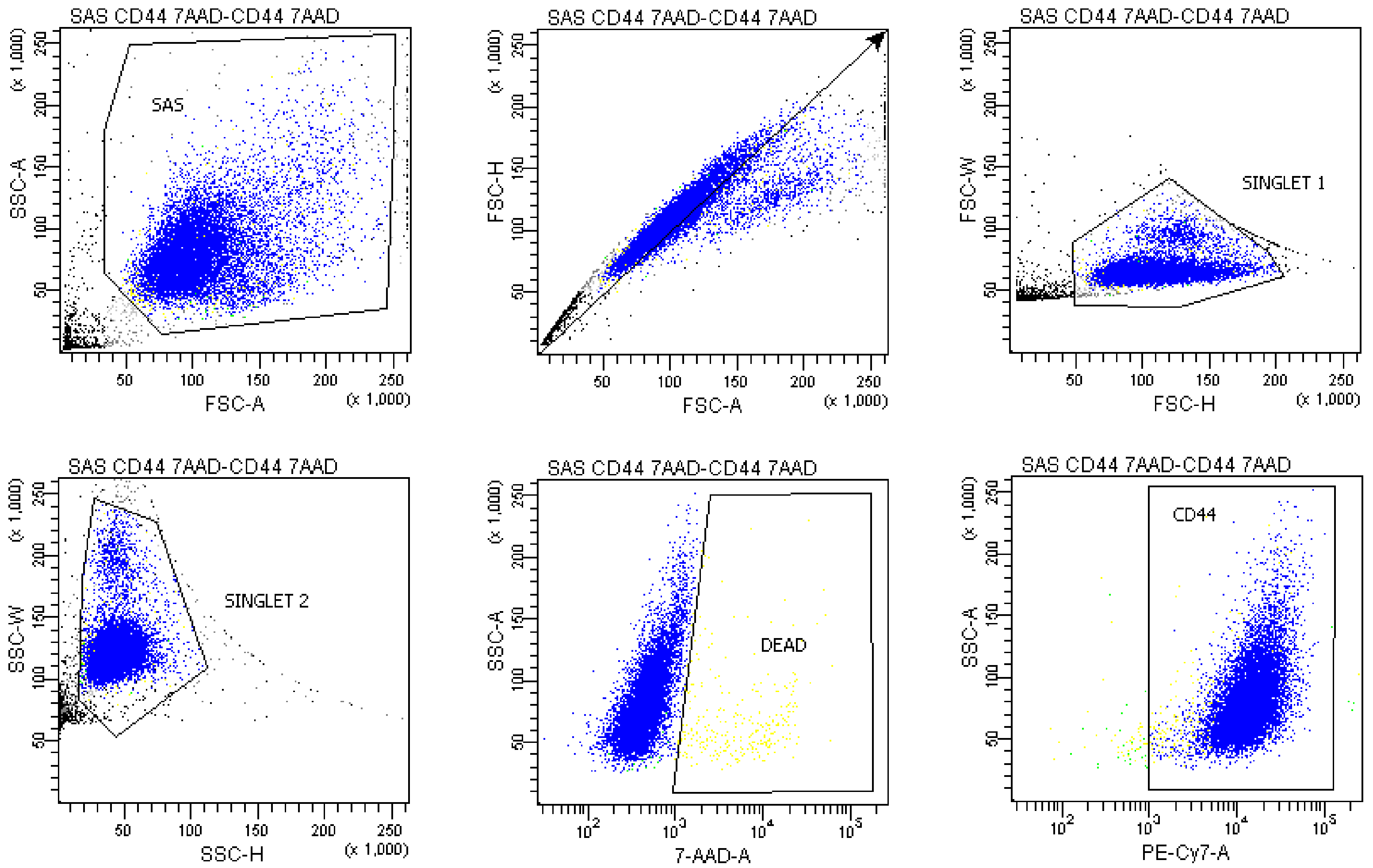


Tube: CD44 7AAD

| Population | #Events | %Parent |
|---|---|---|
| All Events | 11,964 | #### |
| SINGLET 1 | 10,985 | 91.8 |
| SINGLET 2 | 10,793 | 98.3 |
| SAS | 10,712 | 99.2 |
| DEAD | 304 | 2.8 |
| LIVE | 10,408 | 97.2 |
| CD44 | 10,378 | 99.7 |

Experiment Name: 27102017 CD44 7AAD_RUN1
Specimen Name: SAS CD44 7AAD
Tube Name: CD44 7AAD
Record Date: Oct 27, 2017 11:54:48 AM
$OP: ToxicologyLab

| Population | #Events | %Parent | FSC-H Mean | SSC-A Mean |
|---|---|---|---|---|
| All Events | 11,964 | #### | 103,014 | 77,705 |
| SINGLET 1 | 10,985 | 91.8 | 109,465 | 81,904 |
| SINGLET 2 | 10,793 | 98.3 | 109,541 | 81,159 |
| SAS | 10,712 | 99.2 | 109,684 | 81,302 |
| DEAD | 304 | 2.8 | 104,029 | 69,331 |
| LIVE | 10,408 | 97.2 | 109,849 | 81,652 |
| CD44 | 10,378 | 99.7 | 109,811 | 81,731 |
