# Supplementary material for: Mitochondrial DNA alterations may influence the cisplatin responsiveness of oral squamous cell carcinoma
Source: Sci Rep. 2020 May 12;10:7885. doi: 10.1038/s41598-020-64664-3 (PMC7217862; doi:10.1038/s41598-020-64664-3)
Supplement: Supplementary file 9 — Dataset S8. [file 41598_2020_64664_MOESM9_ESM.zip › Supplementary Dataset S8/SINGLE COLOR FLOW CYTOMETRY CD44 SURFACE MARKER ANALYSIS/PARENTAL SAS/EXP1 PARENTAL SAS CONTROL.pdf]

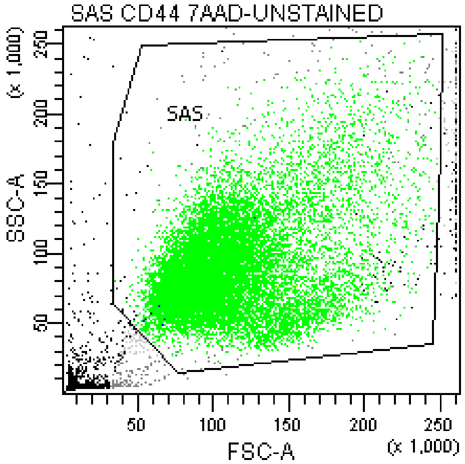

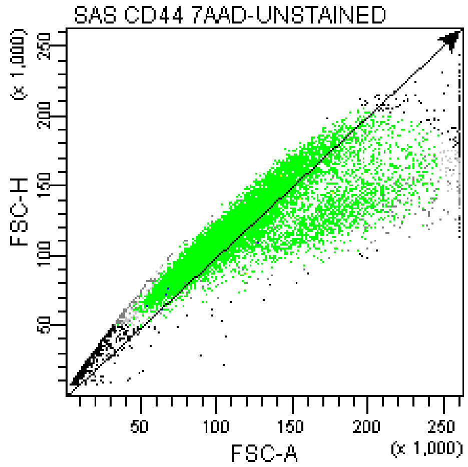

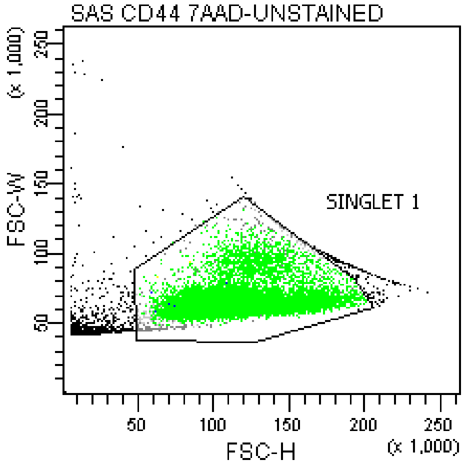

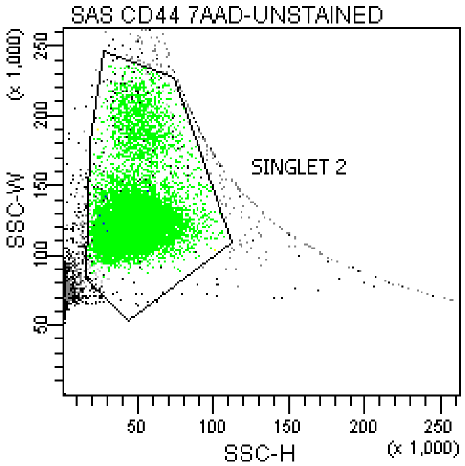

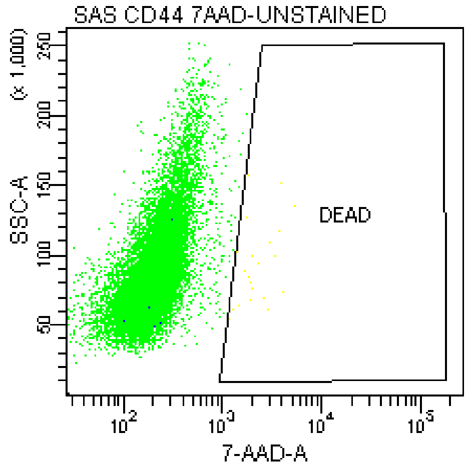

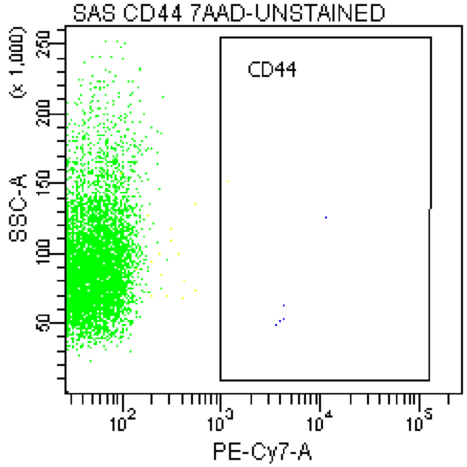

Tube: UNSTAINED

Population	#Events	%Parent
All Events	18,513	####
SINGLET 1	17,077	92.2
SINGLET 2	16,698	97.8
SAS	16,566	99.2
DEAD	25	0.2
LIVE	16,541	99.8
CD44	5	0.0

Experiment Name: 27102017 CD44 7AAD_RUN1
Specimen Name: SAS CD44 7AAD
Tube Name: UNSTAINED
Record Date: Oct 27, 2017 11:46:54 AM
$OP: ToxicologyLab

Population	#Events	%Parent	FSC-H Mean	SSC-A Mean
All Events	18,513	####	107,509	85,763
SINGLET 1	17,077	92.2	112,907	90,001
SINGLET 2	16,698	97.8	112,940	88,435
SAS	16,566	99.2	113,024	88,385
DEAD	25	0.2	112,359	89,893
LIVE	16,541	99.8	113,025	88,382
CD44	5	0.0	74,826	66,608
