# Supplementary material for: Mitochondrial DNA alterations may influence the cisplatin responsiveness of oral squamous cell carcinoma
Source: Sci Rep. 2020 May 12;10:7885. doi: 10.1038/s41598-020-64664-3 (PMC7217862; doi:10.1038/s41598-020-64664-3)
Supplement: Supplementary file 9 — Dataset S8. [file 41598_2020_64664_MOESM9_ESM.zip › Supplementary Dataset S8/SINGLE COLOR FLOW CYTOMETRY CD44 SURFACE MARKER ANALYSIS/PARENTAL SAS/EXP2 PARENTAL SAS CD44.pdf]

FACSDiva Version 6.1.3

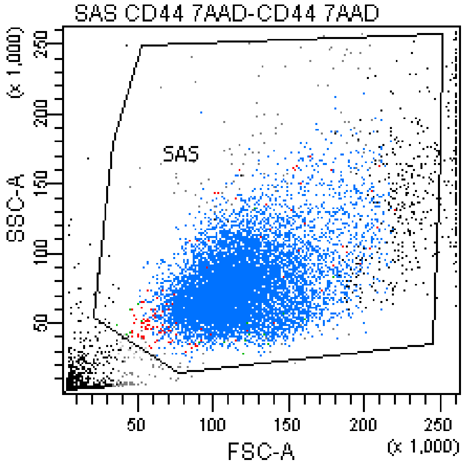

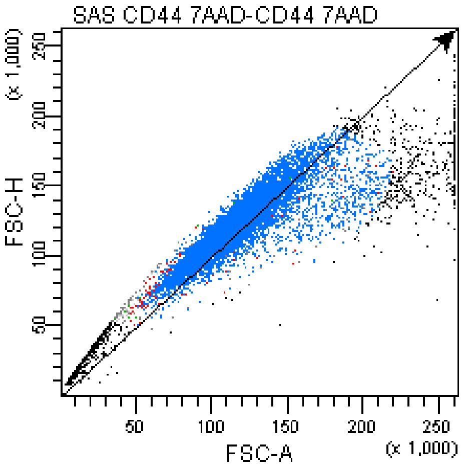

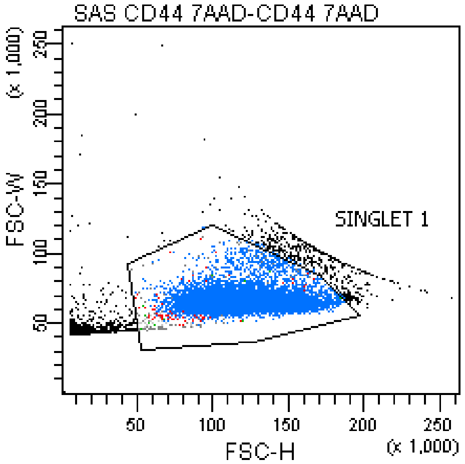

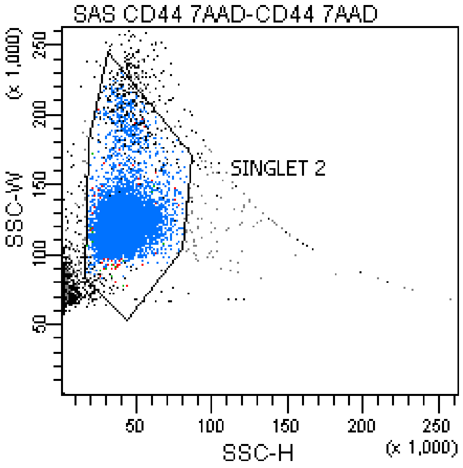

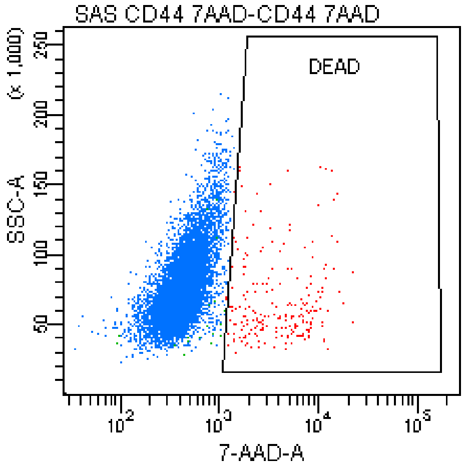

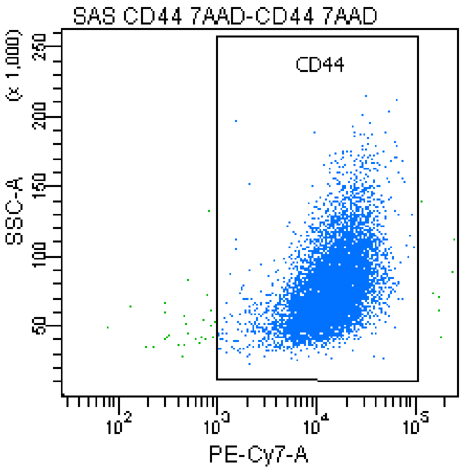

Experiment Name:	27102017 CD44 7AAD_RUN2
Specimen Name:	SAS CD44 7AAD
Tube Name:	CD44 7AAD
Record Date:	Oct 27, 2017 11:31:47 AM
$OP:	ToxicologyLab

Population	#Events	%Parent	FSC-H Mean	SSC-A Mean
All Events	12,080	####	112,636	73,940
SINGLET 1	10,646	88.1	118,472	75,794
SINGLET 2	10,441	98.1	118,696	74,896
SAS	10,421	99.8	118,800	74,984
DEAD	225	2.2	96,870	67,317
LIVE	10,196	97.8	119,284	75,153
CD44	10,159	99.6	119,276	75,220

Tube: CD44 7AAD

Population	#Events	%Parent
All Events	12,080	####
SINGLET 1	10,646	88.1
SINGLET 2	10,441	98.1
SAS	10,421	99.8
DEAD	225	2.2
LIVE	10,196	97.8
CD44	10,159	99.6
