# Supplementary material for: Mitochondrial DNA alterations may influence the cisplatin responsiveness of oral squamous cell carcinoma
Source: Sci Rep. 2020 May 12;10:7885. doi: 10.1038/s41598-020-64664-3 (PMC7217862; doi:10.1038/s41598-020-64664-3)
Supplement: Supplementary file 9 — Dataset S8. [file 41598_2020_64664_MOESM9_ESM.zip › Supplementary Dataset S8/SINGLE COLOR FLOW CYTOMETRY CD44 SURFACE MARKER ANALYSIS/PARENTAL SAS/EXP2 PARENTAL SAS CONTROL.pdf]

FACSDiva Version 6.1.3

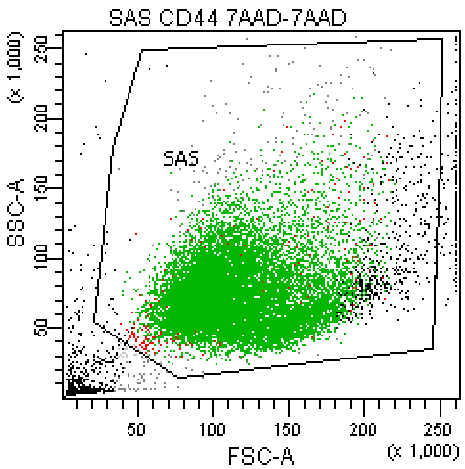

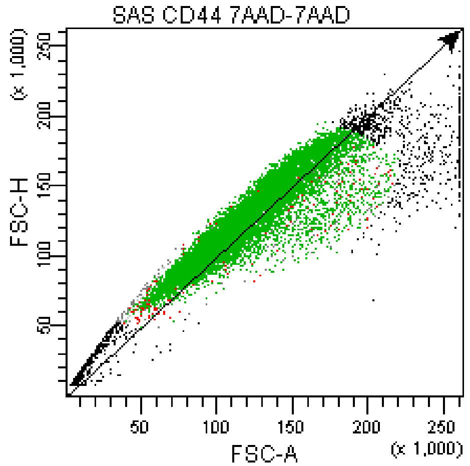

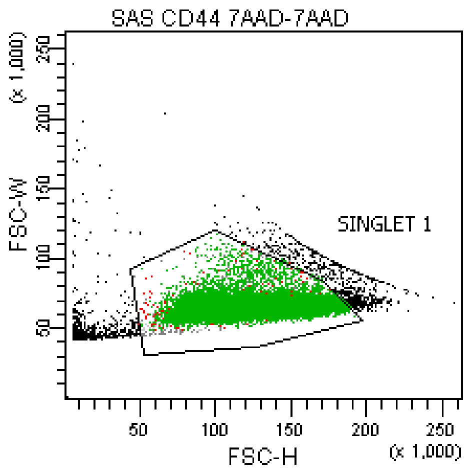

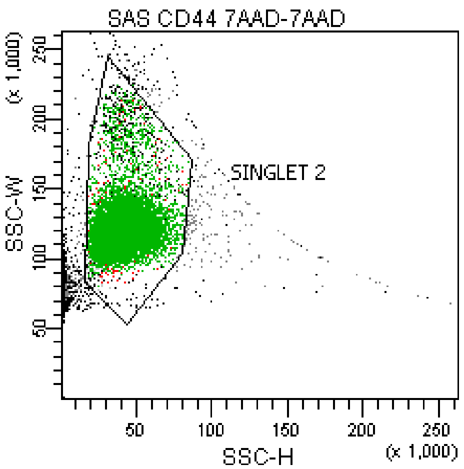

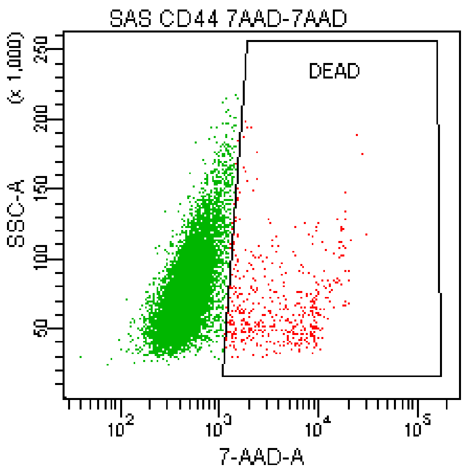

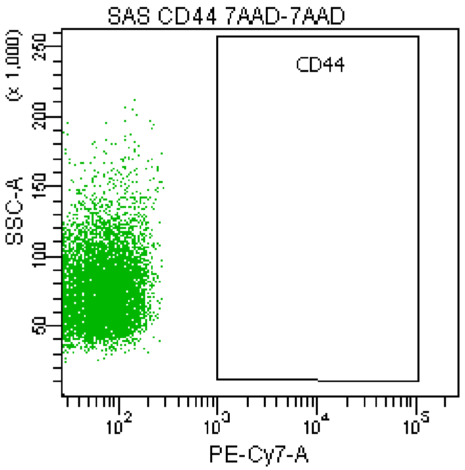

Experiment Name:	27102017 CD44 7AAD_RUN2
Specimen Name:	SAS CD44 7AAD
Tube Name:	7AAD
Record Date:	Oct 27, 2017 11:27:50 AM
$OP:	ToxicologyLab

Population	#Events	%Parent	FSC-H Mean	SSC-A Mean
All Events	17,805	####	116,791	73,657
SINGLET 1	16,200	91.0	119,365	74,887
SINGLET 2	15,927	98.3	119,518	73,782
SAS	15,899	99.8	119,620	73,858
DEAD	514	3.2	103,755	70,945
LIVE	15,385	96.8	120,150	73,955
CD44	0	0.0	####	####

Tube: 7AAD

Population	#Events	%Parent
All Events	17,805	####
SINGLET 1	16,200	91.0
SINGLET 2	15,927	98.3
SAS	15,899	99.8
DEAD	514	3.2
LIVE	15,385	96.8
CD44	0	0.0
