# Supplementary material for: Mitochondrial DNA alterations may influence the cisplatin responsiveness of oral squamous cell carcinoma
Source: Sci Rep. 2020 May 12;10:7885. doi: 10.1038/s41598-020-64664-3 (PMC7217862; doi:10.1038/s41598-020-64664-3)
Supplement: Supplementary file 9 — Dataset S8. [file 41598_2020_64664_MOESM9_ESM.zip › Supplementary Dataset S8/SINGLE COLOR FLOW CYTOMETRY CD44 SURFACE MARKER ANALYSIS/PARENTAL SAS/EXP3 PARENTAL SAS CD44.pdf]

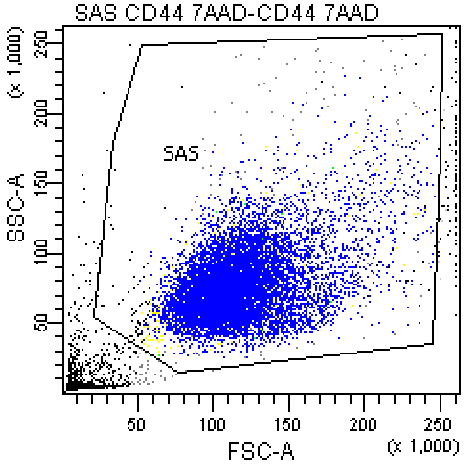

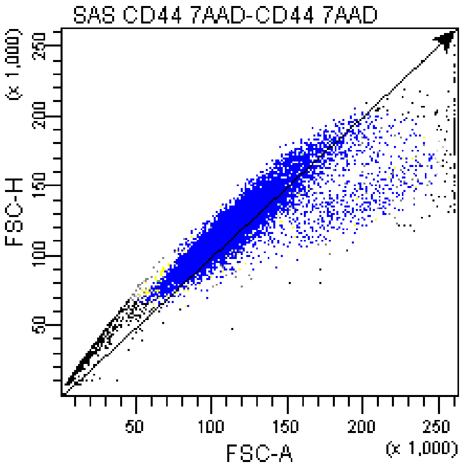

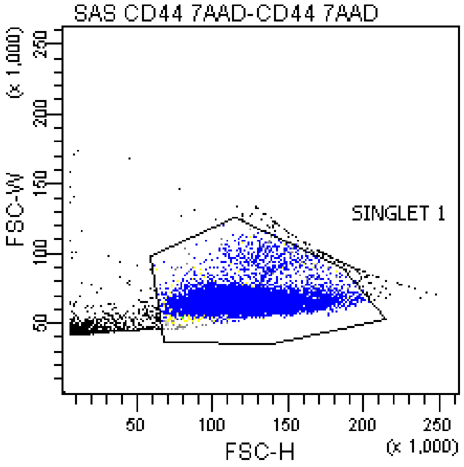

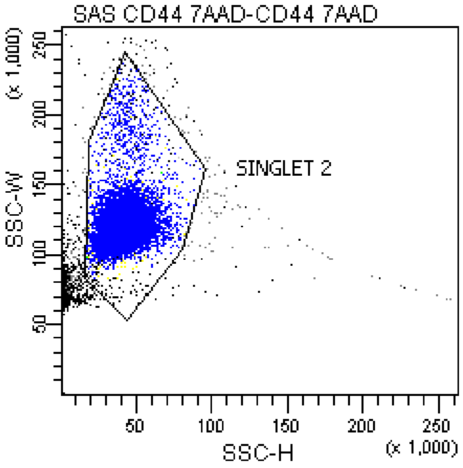

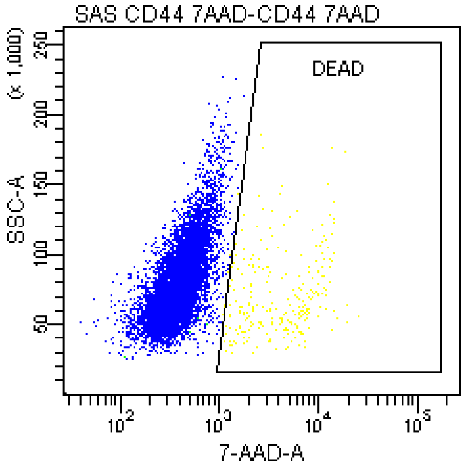

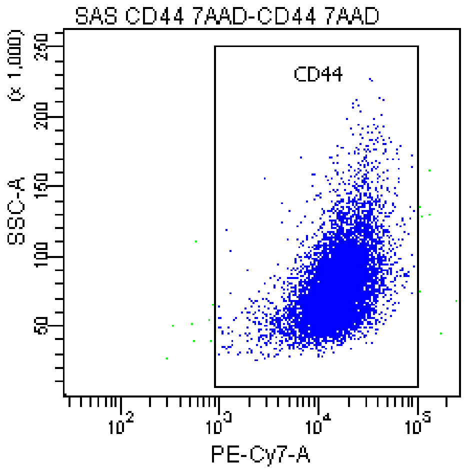

Tube: CD44 7AAD

Population	#Events	%Parent
All Events	11,993	####
SINGLET 1	10,739	89.5
SINGLET 2	10,580	98.5
SAS	10,567	99.9
DEAD	262	2.5
LIVE	10,305	97.5
CD44	10,290	99.9

Experiment Name:	27102017 CD44 7AAD_RUN3
Specimen Name:	SAS CD44 7AAD
Tube Name:	CD44 7AAD
Record Date:	Oct 27, 2017 11:42:41 AM
$OP:	ToxicologyLab

Population	#Events	%Parent	FSC-H Mean	SSC-A Mean
All Events	11,993	####	111,586	72,757
SINGLET 1	10,739	89.5	120,059	77,512
SINGLET 2	10,580	98.5	120,051	76,571
SAS	10,567	99.9	120,036	76,554
DEAD	262	2.5	99,806	70,556
LIVE	10,305	97.5	120,550	76,706
CD44	10,290	99.9	120,530	76,706
