# Supplementary material for: Mitochondrial DNA alterations may influence the cisplatin responsiveness of oral squamous cell carcinoma
Source: Sci Rep. 2020 May 12;10:7885. doi: 10.1038/s41598-020-64664-3 (PMC7217862; doi:10.1038/s41598-020-64664-3)
Supplement: Supplementary file 9 — Dataset S8. [file 41598_2020_64664_MOESM9_ESM.zip › Supplementary Dataset S8/SINGLE COLOR FLOW CYTOMETRY CD44 SURFACE MARKER ANALYSIS/PARENTAL SAS/EXP3 PARENTAL SAS CONTROL.pdf]

FACSDiva Version 6.1.3

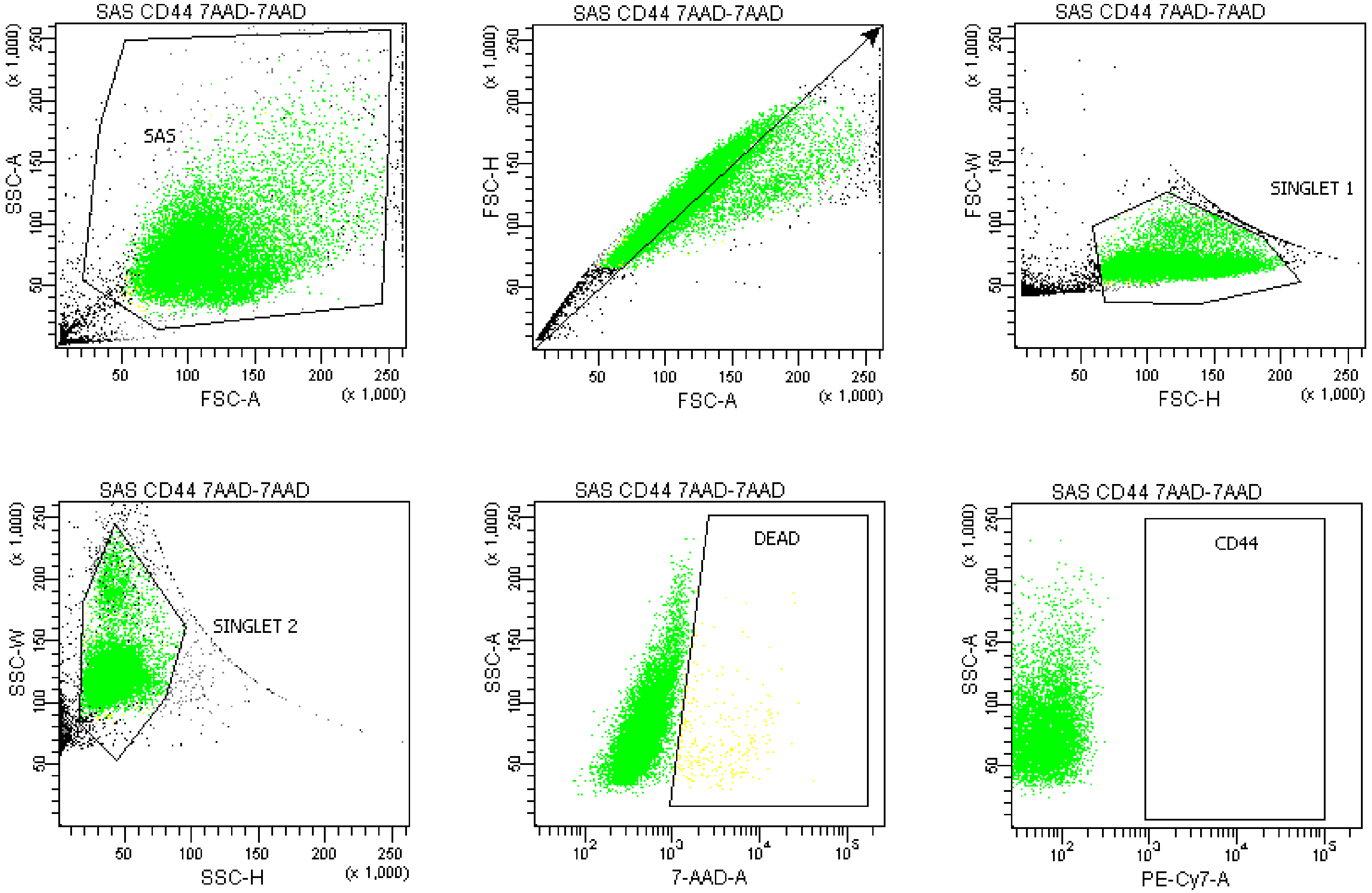

Tube: 7AAD

Population	#Events	%Parent
All Events	18,029	####
SINGLET 1	16,441	91.2
SINGLET 2	16,135	98.1
SAS	16,120	99.9
DEAD	287	1.8
LIVE	15,833	98.2
CD44	0	0.0

Experiment Name: 27102017 CD44 7AAD_RUN3
Specimen Name: SAS CD44 7AAD
Tube Name: 7AAD
Record Date: Oct 27, 2017 11:39:50 AM
$OP: ToxicologyLab

Population	#Events	%Parent	FSC-H Mean	SSC-A Mean
All Events	18,029	####	111,357	74,675
SINGLET 1	16,441	91.2	116,917	77,140
SINGLET 2	16,135	98.1	116,844	75,724
SAS	16,120	99.9	116,801	75,694
DEAD	287	1.8	103,714	72,942
LIVE	15,833	98.2	117,038	75,744
CD44	0	0.0	####	####
