# Supplementary material for: Mitochondrial DNA alterations may influence the cisplatin responsiveness of oral squamous cell carcinoma
Source: Sci Rep. 2020 May 12;10:7885. doi: 10.1038/s41598-020-64664-3 (PMC7217862; doi:10.1038/s41598-020-64664-3)
Supplement: Supplementary file 9 — Dataset S8. [file 41598_2020_64664_MOESM9_ESM.zip › Supplementary Dataset S8/SINGLE COLOR FLOW CYTOMETRY CD44 SURFACE MARKER ANALYSIS/TUMOR SPHERE/EXP1 TUMOR SPHERE CD44.pdf]

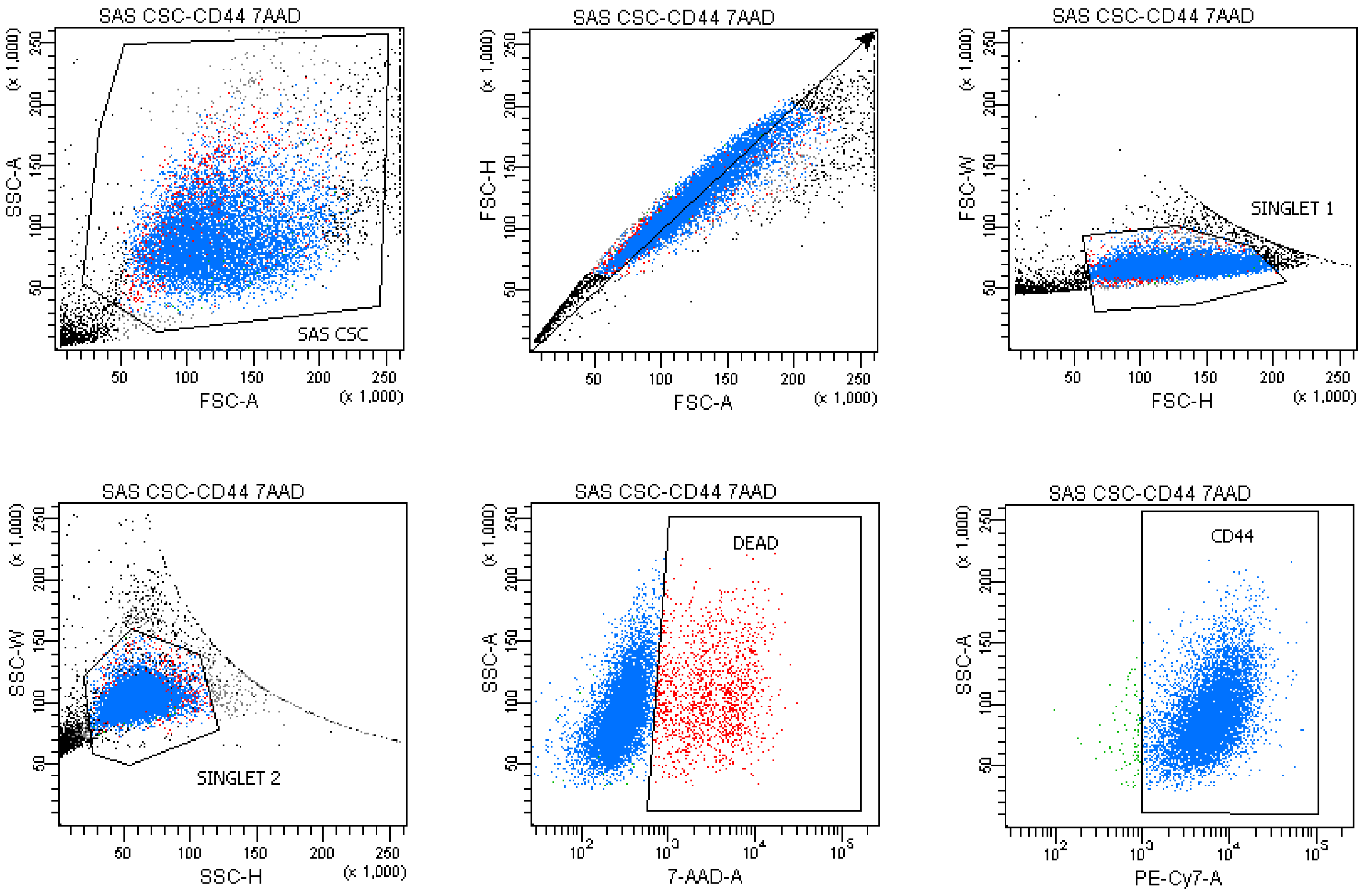
SAS CSC-CD44 7AAD
SSC-A (x 1,000)
FSC-A (x 1,000)
SAS CSC
SAS CSC-CD44 7AAD
FSC-H (x 1,000)
FSC-A (x 1,000)
SAS CSC-CD44 7AAD
FSC-W (x 1,000)
FSC-H (x 1,000)
SINGLET 1
SAS CSC-CD44 7AAD
SSC-W (x 1,000)
SSC-H (x 1,000)
SINGLET 2
SAS CSC-CD44 7AAD
SSC-A (x 1,000)
7-AAD-A
DEAD
SAS CSC-CD44 7AAD
SSC-A (x 1,000)
PE-Cy7-A
CD44

Experiment Name: 02112017 SAS CSC CD44 7AAD_RUN1
Specimen Name: SAS CSC
Tube Name: CD44 7AAD
Record Date: Nov 2, 2017 10:24:53 AM
$OP: ToxicologyLab

Population	#Events	%Parent	FSC-H Mean	SSC-A Mean
All Events	12,431	####	114,705	92,268
SINGLET 1	10,573	85.1	120,706	94,879
SINGLET 2	10,202	96.5	120,470	91,825
SAS CSC	10,194	99.9	120,511	91,874
DEAD	1,452	14.2	110,437	106,829
LIVE	8,742	85.8	122,184	89,390
CD44	8,667	99.1	122,042	89,516

Tube: CD44 7AAD

Population	#Events	%Parent
All Events	12,431	####
SINGLET 1	10,573	85.1
SINGLET 2	10,202	96.5
SAS CSC	10,194	99.9
DEAD	1,452	14.2
LIVE	8,742	85.8
CD44	8,667	99.1
