# Supplementary material for: Mitochondrial DNA alterations may influence the cisplatin responsiveness of oral squamous cell carcinoma
Source: Sci Rep. 2020 May 12;10:7885. doi: 10.1038/s41598-020-64664-3 (PMC7217862; doi:10.1038/s41598-020-64664-3)
Supplement: Supplementary file 9 — Dataset S8. [file 41598_2020_64664_MOESM9_ESM.zip › Supplementary Dataset S8/SINGLE COLOR FLOW CYTOMETRY CD44 SURFACE MARKER ANALYSIS/TUMOR SPHERE/EXP1 TUMOR SPHERE CONTROL.pdf]

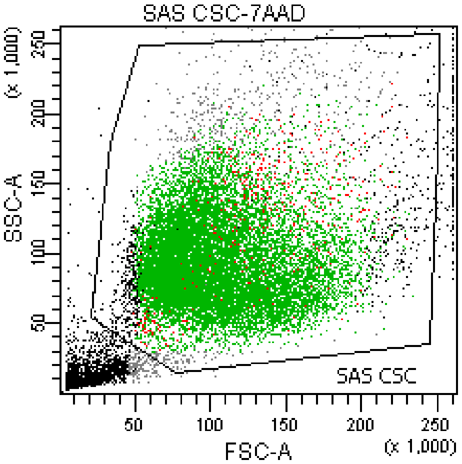
SAS CSC-7AAD
SAS CSC
SSC-A
(x 1,000)
FSC-A
(x 1,000)
50 100 150 200 250

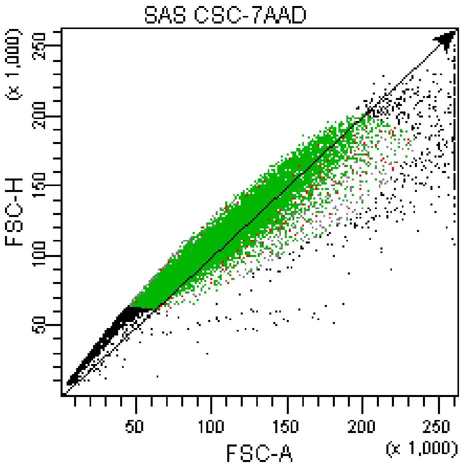
SAS CSC-7AAD
FSC-H
(x 1,000)
FSC-A
(x 1,000)
50 100 150 200 250

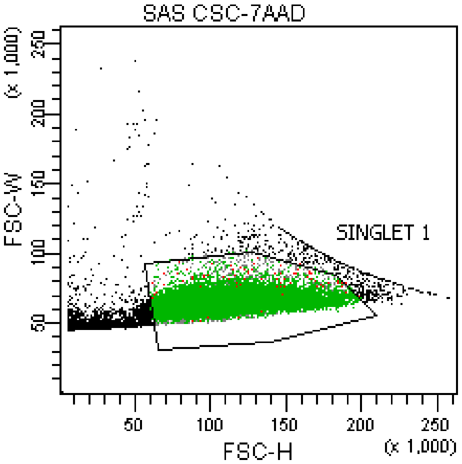
SAS CSC-7AAD
SINGLET 1
FSC-W
(x 1,000)
FSC-H
(x 1,000)
50 100 150 200 250

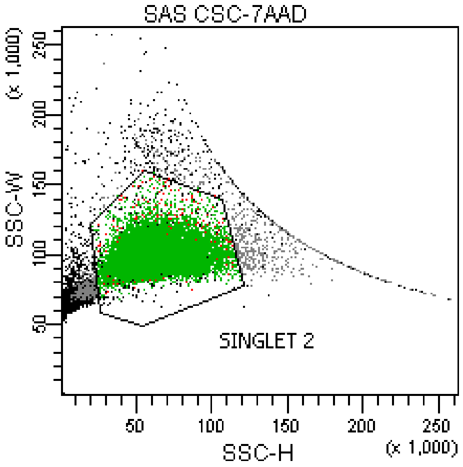
SAS CSC-7AAD
SINGLET 2
SSC-W
(x 1,000)
SSC-H
(x 1,000)
50 100 150 200 250

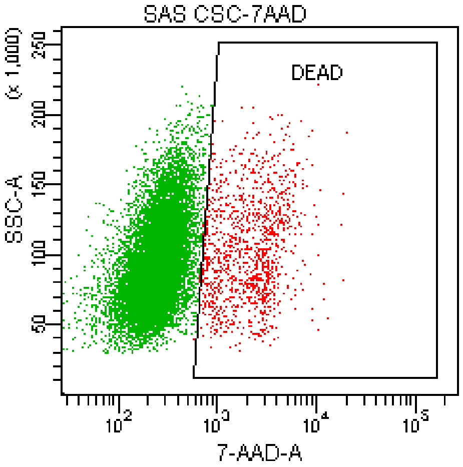
SAS CSC-7AAD
DEAD
SSC-A
(x 1,000)
7-AAD-A

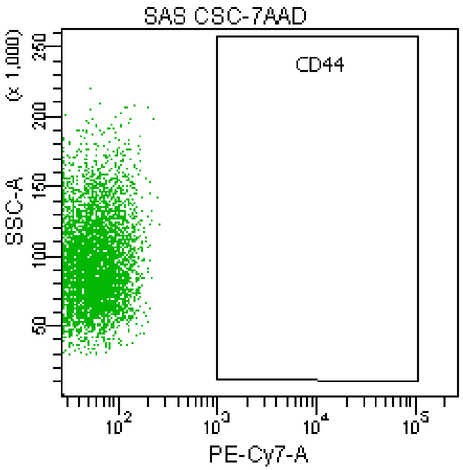
SAS CSC-7AAD
CD44
SSC-A
(x 1,000)
PE-Cy7-A

Experiment Name: 02112017 SAS CSC CD44 7AAD_RUN1
Specimen Name: SAS CSC
Tube Name: 7AAD
Record Date: Nov 2, 2017 10:23:04 AM
$OP: ToxicologyLab

Population	#Events	%Parent	FSC-H Mean	SSC-A Mean
All Events	19,354	####	98,905	87,919
SINGLET 1	15,761	81.4	109,414	96,908
SINGLET 2	14,920	94.7	109,115	93,420
SAS CSC	14,908	99.9	109,146	93,472
DEAD	1,231	8.3	109,988	103,446
LIVE	13,677	91.7	109,070	92,574
CD44	0	0.0	####	####

Tube: 7AAD

Population	#Events	%Parent
All Events	19,354	####
SINGLET 1	15,761	81.4
SINGLET 2	14,920	94.7
SAS CSC	14,908	99.9
DEAD	1,231	8.3
LIVE	13,677	91.7
CD44	0	0.0
