# Supplementary material for: Mitochondrial DNA alterations may influence the cisplatin responsiveness of oral squamous cell carcinoma
Source: Sci Rep. 2020 May 12;10:7885. doi: 10.1038/s41598-020-64664-3 (PMC7217862; doi:10.1038/s41598-020-64664-3)
Supplement: Supplementary file 9 — Dataset S8. [file 41598_2020_64664_MOESM9_ESM.zip › Supplementary Dataset S8/SINGLE COLOR FLOW CYTOMETRY CD44 SURFACE MARKER ANALYSIS/TUMOR SPHERE/EXP2 TUMOR SPHERE CD44.pdf]

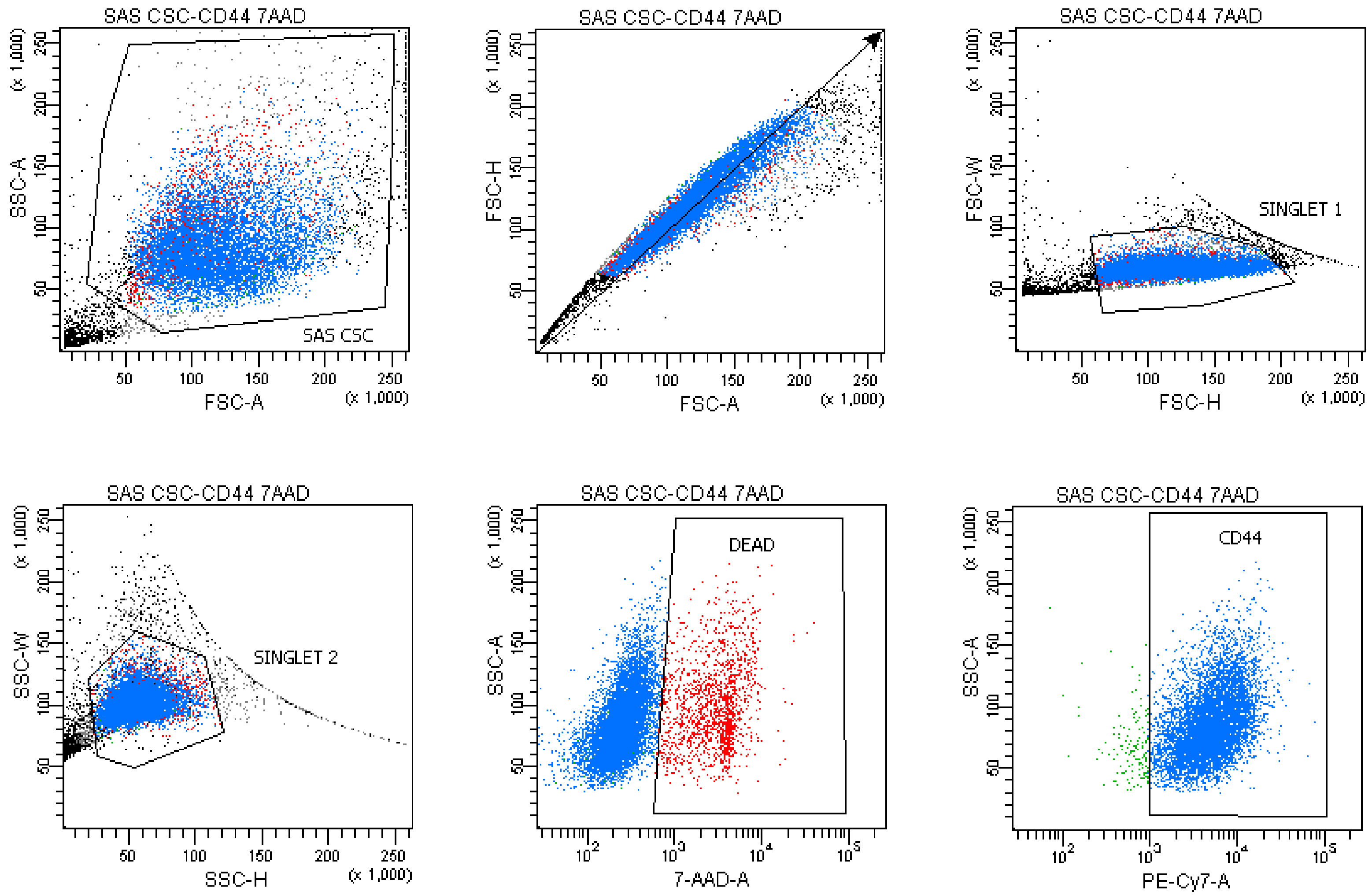
SAS CSC-CD44 7AAD
SSC-A (x 1,000)
FSC-A (x 1,000)
SAS CSC
SAS CSC-CD44 7AAD
FSC-H (x 1,000)
FSC-A (x 1,000)
SAS CSC-CD44 7AAD
FSC-W (x 1,000)
FSC-H (x 1,000)
SINGLET 1
SAS CSC-CD44 7AAD
SSC-W (x 1,000)
SSC-H (x 1,000)
SINGLET 2
SAS CSC-CD44 7AAD
SSC-A (x 1,000)
7-AAD-A
DEAD
SAS CSC-CD44 7AAD
SSC-A (x 1,000)
PE-Cy7-A
CD44

Experiment Name: 02112017 SAS CSC CD44 7AAD_RUN2
Specimen Name: SAS CSC
Tube Name: CD44 7AAD
Record Date: Nov 2, 2017 10:31:50 AM
$OP: ToxicologyLab

Population	#Events	%Parent	FSC-H Mean	SSC-A Mean
All Events	11,980	####	112,378	86,375
SINGLET 1	10,396	86.8	118,018	88,542
SINGLET 2	10,011	96.3	117,857	86,075
SAS CSC	10,000	99.9	117,911	86,139
DEAD	1,628	16.3	105,386	98,733
LIVE	8,372	83.7	120,347	83,690
CD44	8,238	98.4	120,110	83,968

Tube: CD44 7AAD

Population	#Events	%Parent
All Events	11,980	####
SINGLET 1	10,396	86.8
SINGLET 2	10,011	96.3
SAS CSC	10,000	99.9
DEAD	1,628	16.3
LIVE	8,372	83.7
CD44	8,238	98.4
