# Supplementary material for: Mitochondrial DNA alterations may influence the cisplatin responsiveness of oral squamous cell carcinoma
Source: Sci Rep. 2020 May 12;10:7885. doi: 10.1038/s41598-020-64664-3 (PMC7217862; doi:10.1038/s41598-020-64664-3)
Supplement: Supplementary file 9 — Dataset S8. [file 41598_2020_64664_MOESM9_ESM.zip › Supplementary Dataset S8/SINGLE COLOR FLOW CYTOMETRY CD44 SURFACE MARKER ANALYSIS/TUMOR SPHERE/EXP2 TUMOR SPHERE CONTROL.pdf]

FACSDiva Version 6.1.3

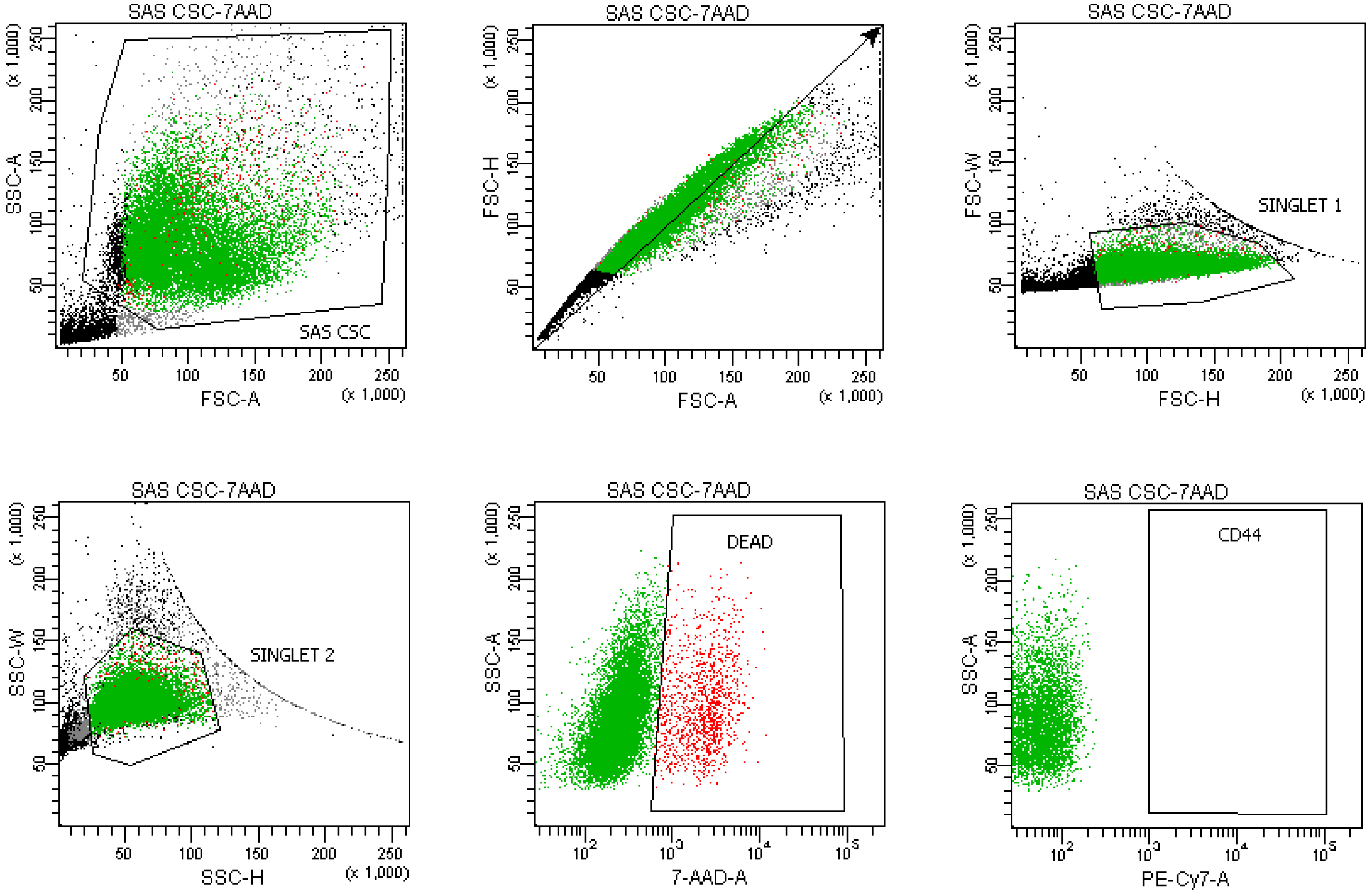

Experiment Name: 02112017 SAS CSC CD44 7AAD_RUN2
Specimen Name: SAS CSC
Tube Name: 7AAD
Record Date: Nov 2, 2017 10:30:14 AM
$OP: ToxicologyLab

Population	#Events	%Parent	FSC-H Mean	SSC-A Mean
All Events	20,916	####	88,735	77,586
SINGLET 1	15,813	75.6	103,130	87,995
SINGLET 2	15,011	94.9	102,766	85,395
SAS CSC	15,000	99.9	102,792	85,435
DEAD	1,299	8.7	98,423	97,190
LIVE	13,701	91.3	103,206	84,321
CD44	0	0.0	####	####

Tube: 7AAD

Population	#Events	%Parent
All Events	20,916	####
SINGLET 1	15,813	75.6
SINGLET 2	15,011	94.9
SAS CSC	15,000	99.9
DEAD	1,299	8.7
LIVE	13,701	91.3
CD44	0	0.0
