# Supplementary material for: Mitochondrial DNA alterations may influence the cisplatin responsiveness of oral squamous cell carcinoma
Source: Sci Rep. 2020 May 12;10:7885. doi: 10.1038/s41598-020-64664-3 (PMC7217862; doi:10.1038/s41598-020-64664-3)
Supplement: Supplementary file 9 — Dataset S8. [file 41598_2020_64664_MOESM9_ESM.zip › Supplementary Dataset S8/SINGLE COLOR FLOW CYTOMETRY CD44 SURFACE MARKER ANALYSIS/TUMOR SPHERE/EXP3 TUMOR SPHERE CD44.pdf]

FACSDiva Version 6.1.3

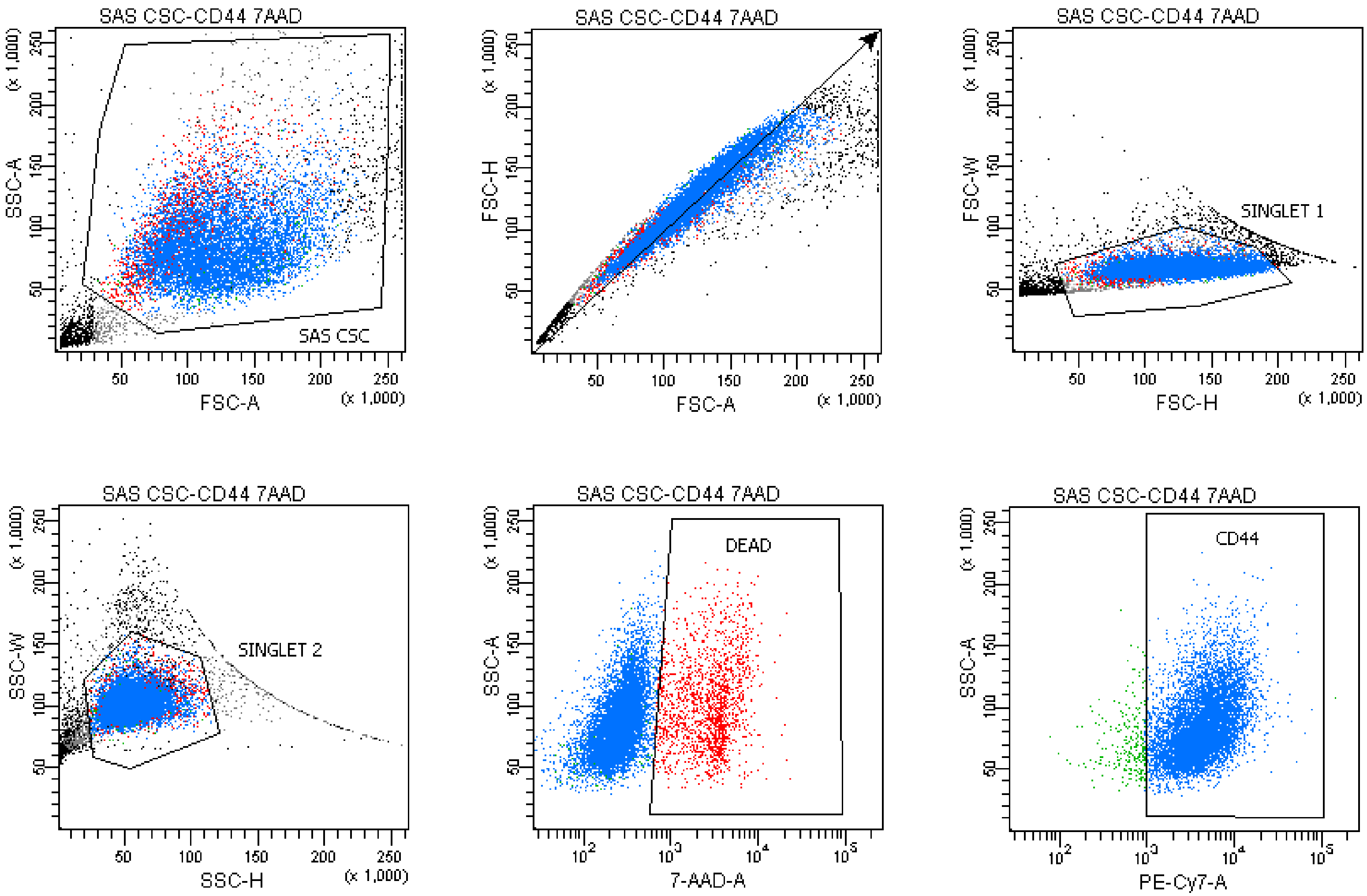

Experiment Name: 02112017 SAS CSC CD44 7AAD_RUN3
Specimen Name: SAS CSC
Tube Name: CD44 7AAD
Record Date: Nov 2, 2017 10:37:57 AM
$OP: ToxicologyLab

Population	#Events	%Parent	FSC-H Mean	SSC-A Mean
All Events	12,152	####	114,100	87,327
SINGLET 1	10,667	87.8	118,697	88,171
SINGLET 2	10,170	95.3	119,421	86,325
SAS CSC	10,123	99.5	119,735	86,570
DEAD	1,729	17.1	99,890	99,988
LIVE	8,394	82.9	123,822	83,806
CD44	8,142	97.0	123,342	84,241

Tube: CD44 7AAD

Population	#Events	%Parent
All Events	12,152	####
SINGLET 1	10,667	87.8
SINGLET 2	10,170	95.3
SAS CSC	10,123	99.5
DEAD	1,729	17.1
LIVE	8,394	82.9
CD44	8,142	97.0
