# Supplementary material for: Mitochondrial DNA alterations may influence the cisplatin responsiveness of oral squamous cell carcinoma
Source: Sci Rep. 2020 May 12;10:7885. doi: 10.1038/s41598-020-64664-3 (PMC7217862; doi:10.1038/s41598-020-64664-3)
Supplement: Supplementary file 9 — Dataset S8. [file 41598_2020_64664_MOESM9_ESM.zip › Supplementary Dataset S8/SINGLE COLOR FLOW CYTOMETRY CD44 SURFACE MARKER ANALYSIS/TUMOR SPHERE/EXP3 TUMOR SPHERE CONTROL.pdf]

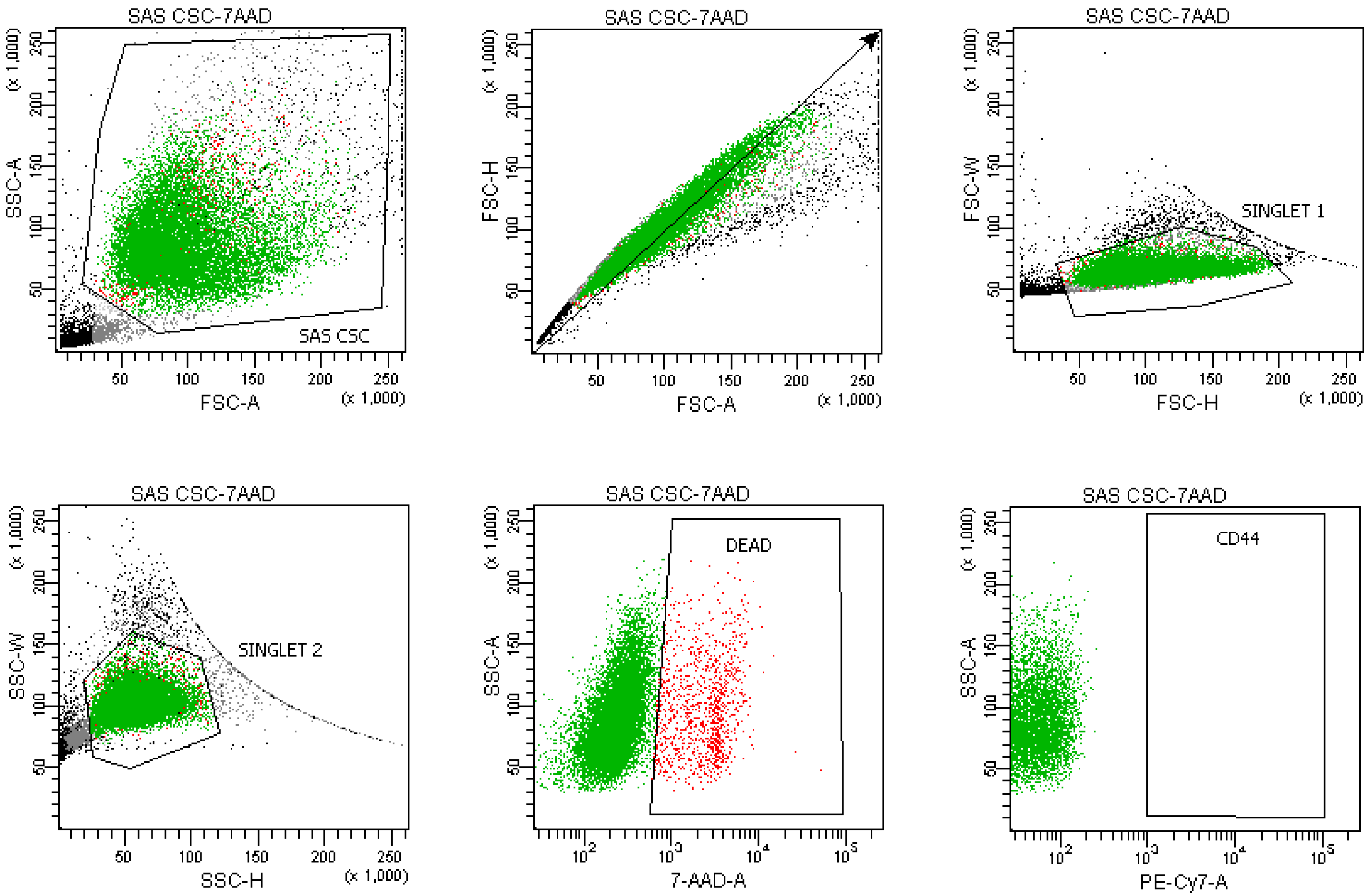
SAS CSC-7AAD
SSC-A (x 1,000)
FSC-A (x 1,000)
SAS CSC
SAS CSC-7AAD
FSC-H (x 1,000)
FSC-A (x 1,000)
SAS CSC-7AAD
FSC-W (x 1,000)
FSC-H (x 1,000)
SINGLET 1
SAS CSC-7AAD
SSC-W (x 1,000)
SSC-H (x 1,000)
SINGLET 2
SAS CSC-7AAD
SSC-A (x 1,000)
7-AAD-A
DEAD
SAS CSC-7AAD
SSC-A (x 1,000)
PE-Cy7-A
CD44

Experiment Name: 02112017 SAS CSC CD44 7AAD_RUN3
Specimen Name: SAS CSC
Tube Name: 7AAD
Record Date: Nov 2, 2017 10:36:24 AM
$OP: ToxicologyLab

Population	#Events	%Parent	FSC-H Mean	SSC-A Mean
All Events	20,141	####	88,618	81,086
SINGLET 1	16,879	83.8	97,739	88,260
SINGLET 2	15,812	93.7	98,374	87,628
SAS CSC	15,750	99.6	98,556	87,842
DEAD	1,151	7.3	95,387	97,191
LIVE	14,599	92.7	98,806	87,105
CD44	0	0.0	####	####

Tube: 7AAD

Population	#Events	%Parent
All Events	20,141	####
SINGLET 1	16,879	83.8
SINGLET 2	15,812	93.7
SAS CSC	15,750	99.6
DEAD	1,151	7.3
LIVE	14,599	92.7
CD44	0	0.0
